# Nucleophilic Fluoride Anion Delivery from Triazacyclononane‐Supported Molecular Ca–F Complexes

**DOI:** 10.1002/anie.202414790

**Published:** 2024-11-09

**Authors:** Omar Apolinar, Job J. C. Struijs, Debotra Sarkar, Véronique Gouverneur, Simon Aldridge

**Affiliations:** ^1^ Chemistry Research Laboratory Department of Chemistry University of Oxford South Parks Road Oxford OX1 3QR UK

**Keywords:** calcium, fluorination, TACN, coordination chemistry

## Abstract

The major source of fluoride, namely calcium difluoride (CaF_2_), is derived from the mineral fluorspar. Recent advances in the activation of CaF_2_ via mechanochemical methods have inspired investigation of the fundamental properties of Ca–F bonds in molecular complexes. However, the paucity of well‐defined molecular Ca–F‐containing complexes undermines systematic understanding of the factors governing nucleophilic fluoride delivery. Here we report the use of a multidentate bis(phenoxide) ligand based on a 1,4,7‐triazacyclononane (TACN) scaffold for the formation of well‐defined Ca–F complexes and demonstrate their capabilities in fluoride delivery. Key to this synthesis is a desymmetrization step carried out on the TACN ligand within the Ca coordination sphere. A series of stable anionic dinuclear calcium fluoride complexes has then been accessed and characterized by NMR spectroscopy (critically by ^19^F NMR) and X‐ray crystallography. Nucleophilic fluoride delivery can be used to generate C−F, Si–F and S−F bonds, with a notable advance over amide‐derived ligand scaffolds being the reduced extent of side reactions derived from competing attack on the electrophile by the less nucleophilic O‐donor anionic ligand set.

## Graphical abstract

A bis(phenoxide) ligand based on a 1,4,7‐triazacyclononane scaffold is employed for the synthesis of dinuclear calcium fluoride complexes. A bridging Ca‐F−Ca motif is confirmed crystallographically and nucleophilic fluoride delivery demonstrated in the formation of a range of E−F bonds. The less nucleophilic anionic O‐donor set reduces the extent of ligand‐centered side reactions compared to related amide‐derived systems.

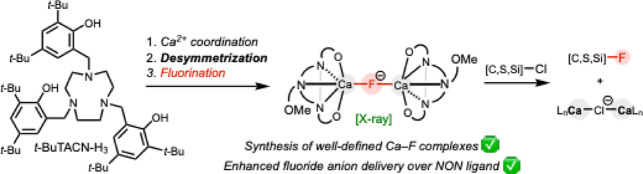

Organofluorine compounds are ubiquitous in our modern world, offering widespread benefits in sectors as diverse as materials, agrochemicals, and pharmaceuticals.[^1^, ^2^] For over two centuries, the industrial process of harnessing fluoride from fluorspar has been reliant on the use of H_2_SO_4_ to generate large quantities of extremely corrosive and dangerous HF.[^3^, ^4^, ^5^, ^6^, ^7^] Recently, mechanochemical methods have been reported which represent an alternative approach for activating CaF_2_, using K_2_HPO_4_ to generate ‘Fluoromix’ in the solid state that can then be used to synthesize fluorochemicals directly.^[8]^ This novel process bypasses HF, and offers an alternative to the current paradigm for fluoride utilization. Notwithstanding such work, there still exists little fundamental understanding of the nucleophilic reactivity of Ca–F entities at the molecular level (particularly in solution), primarily due to CaF_2_ having a high lattice energy (▵*U*_L_=2640 kJ mol^−1^) and poor solubility in organic solvents.[^9^, ^10^, ^11^, ^12^, ^13^] Such investigations could assist in developing methodologies which exploit the nucleophilicity of molecular fluoride‐containing complexes.

Well‐defined Ca–F‐containing molecules remain scarce in the chemical literature, with the few existing reports ranging from inorganic clusters to main group complexes.[^14^, ^15^, ^16^, ^17^, ^18^, ^19^, ^20^, ^21^, ^22^, ^23^, ^24^] Although structurally diverse, such cluster complexes typically display bridging Ca–F bond motifs as part of extended cages. In contrast, the β‐diketiminate systems independently reported by Roesky and by Barrett and Hill offer solubility in hydrocarbon solvents and represented the first well‐defined dimeric Ca–F complexes (Figure 1). However, neither system exemplifies any nucleophilic fluoride anion delivery. Recently, our group reported the synthesis and reactivity of Ca–F‐containing complexes stabilized by the 4,5‐bis(2,6‐diisopropylanilido)‐2,7‐di‐tertbutyl‐9,9‐dimethylxanthene (NON) ligand.^[25]^ The reported complexes are unique in being able to deliver fluoride to electrophilic substrates, to an extent which increases as the Ca–F moiety becomes less sterically congested.

**Figure 1:**
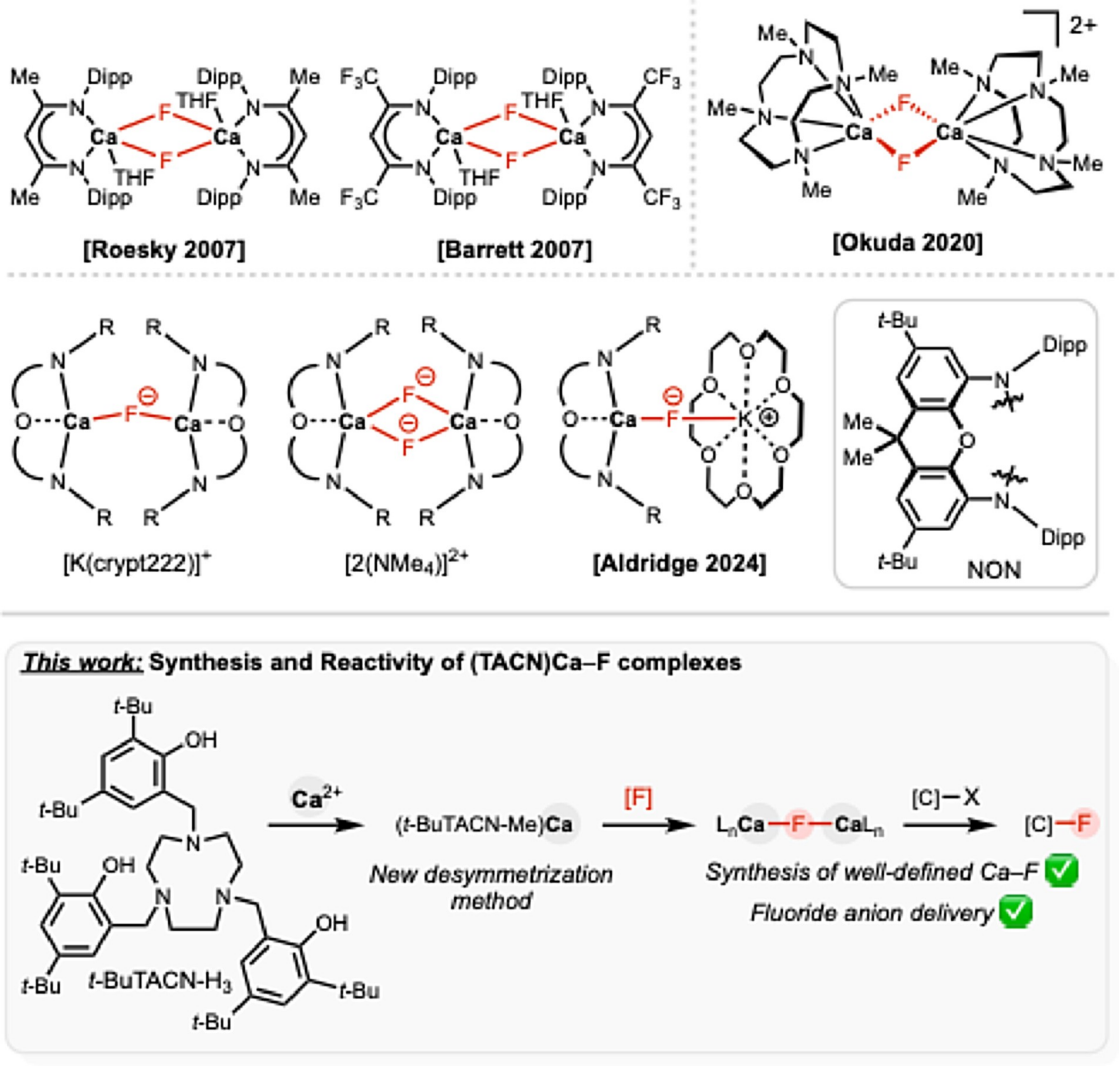
Background and synopsis of the current study.

We wanted to pursue a complementary strategy for the synthesis of well‐defined Ca–F complexes based on O‐donor ligand scaffolds, and to investigate their capabilities for fluoride anion delivery. In contrast to the diamido NON ligand, which typically adopts a meridional geometry when tridentate, we focused on ligand design which would offer anionic O‐ligation, targeting enhanced binding of oxophilic calcium and thus reducing side reactions that involve the ligand as a competing target for electrophilic substrates. Additionally, a greater degree of ligand three‐dimensionality and increased denticity were targeted in order to saturate the Ca^2+^ centre, and reduce the extent of aggregation. With this in mind, we were attracted to macrocyclic ligand scaffolds, notably 1,4,7‐triazacyclononane (TACN) derived ligands. Such donors have been shown to encapsulate metal cations such as Al^3+^ and Ga^3+^, and allow for fluorination and onward applications which extend to ^18^F imaging.[^26^, ^27^] Other closely related macrocyclic ligands such as crowns, azacrowns, or DOTA (CH_2_CH_2_NCH_2_CO_2_H)_4_ are less suitable for the intended application since they do not provide adequate X‐type coordination modes even though they have been shown to encapsulate Ca^2+^ cations.^[28]^ To our knowledge there is only one structurally characterized (TACN)Ca complex,^[29]^ and none containing a Ca–F bond. Herein, we report on synthetic approaches for the generation of anionic (TACN)Ca fluoride complexes, and on the fluoride delivery chemistry thereof.

We initiated our investigation by first synthesizing neutral and anionic (TACN)Ca complexes using a bulky *C*_3_‐symmetric tris(phenol)‐functionalized *t*‐BuTACN‐H_3_ ligand and exploring the fluoride binding capabilities of such systems (Scheme 1). We introduced Ca^2+^ in the form of Ca(HMDS)_2_(THF)_2_ (where HMDS=N(SiMe_3_)_2_), via its reaction with the bulky *C*_3_‐symmetric 1,4,7‐tris(3,5‐tert‐butyl‐2‐hydroxybenzyl)‐1,4,7‐triazacyclononane (*t*‐BuTACN‐H_3_), protio‐ligand **1**. In the presence of an additional equivalent of K[HMDS] this combination generates the anionic complex [(*t*‐BuTACN)Ca]^−^ (**2**, as the [K(η^6^‐benzene)]^+^ salt) in moderate yield (Scheme 1). The hexacoordinate anionic (TACN)Ca component characterized by X‐ray crystallography features non‐crystallographic *C*_3_ symmetry, and sets of three nearly identical Ca–O and Ca–N bonds (ranges: 2.236(1)‐2.264(1) and 2.468(1)‐2.487(1) Å, respectively). The [K(η^6^‐benzene)]^+^ counter‐ion symmetrically caps the [CaO_3_] unit with three K⋅⋅⋅O distances in the range 2.735(1)–2.761(1) Å, and a K⋅⋅⋅centroid separation of 3.257 Å. In addition, we also synthesized the neutral complex (*t*‐BuTACN−H)Ca (**3**), albeit in lower yield, by reacting Ca(HMDS)_2_(THF)_2_ and *t*‐BuTACN‐H_3_ in a 1 : 1 ratio in the absence of exogenous base. This neutral hexacoordinate complex possesses a longer Ca–O(H) bond (2.381(1) Å) between the calcium centre and the pendent protonated phenolic arm (cf. 2.186(1) and 2.271(1) Å for the other Ca–O distances).

**Scheme 1:**
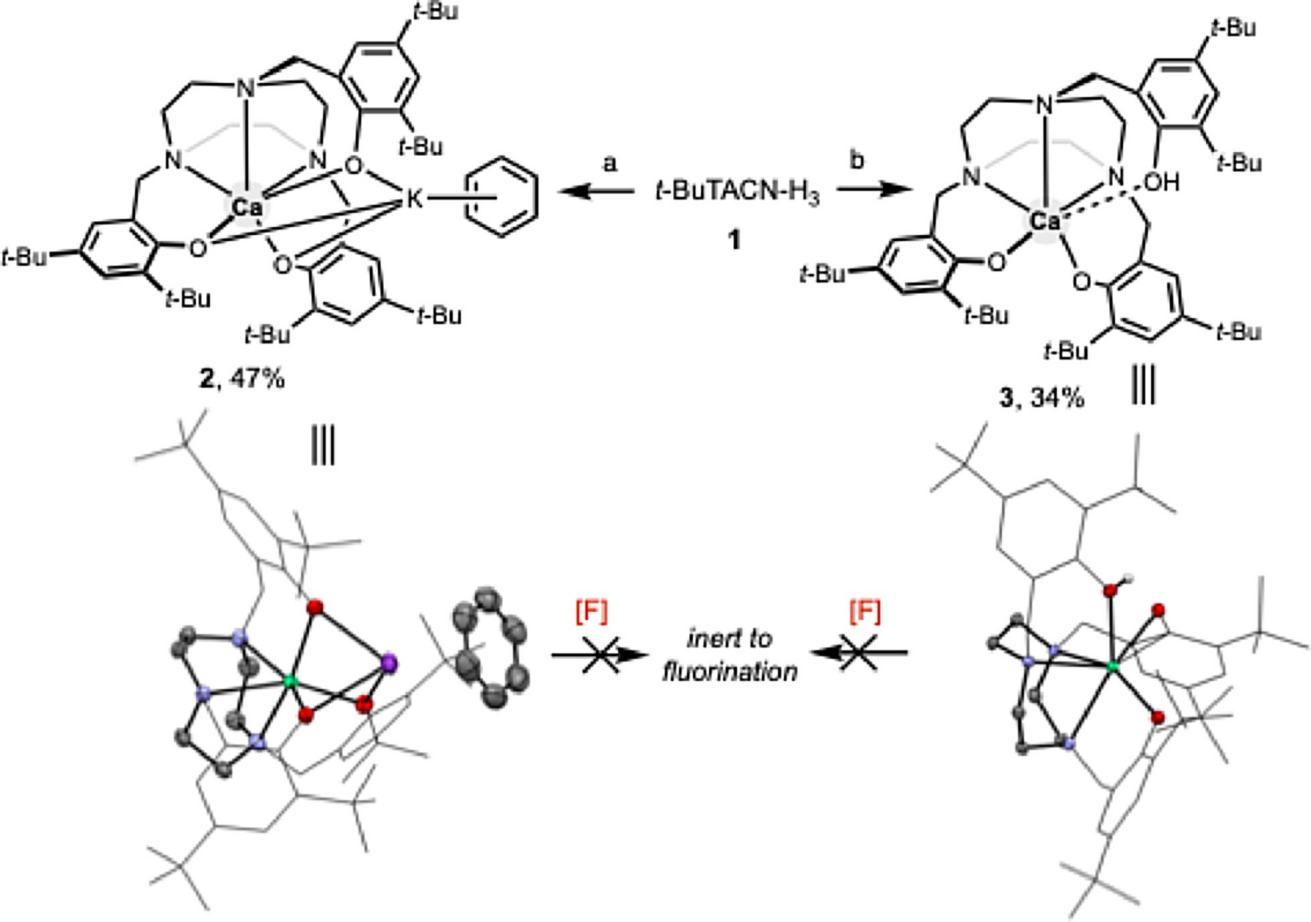
Synthesis and X‐ray structures of anionic and neutral calcium complexes **2** and **3** (ellipsoids set at 50 % probability level; H atoms omitted and arenes shown in wireframe format for clarity). (a) Ca(HMDS)_2_(THF)_2_, KHMDS, and C_6_D_6_. (b) Ca(HMDS)_2_(THF)_2_ and C_6_D_6_.

Compounds **2** and **3** were found to be inert to various fluorination protocols, including the use of KF/18‐crown‐6, trimethyltin fluoride (Me_3_SnF) and tetramethylammonium fluoride (TMAF), each of which has been previously shown to offer the possibility for introducing fluoride into the coordination sphere of calcium.[^18^, ^25^] Presumably, the net anionic charge borne by the (anionic) calcium‐containing component of **2** renders the corresponding dianionic Ca–F‐containing species inaccessible. Neutral complex **3**, on the other hand, yields HF and protio‐ligand under similar conditions (see Figures S32 and S33 for details).

To circumvent these complications, we turned our attention to desymmetrizing the *t*‐BuTACN‐H_3_ ligand, with the aim of generating an L_3_L′X_2_ framework, which on introduction of the Ca^2+^ centre would yield a neutral (TACN)Ca complex without an acidic OH bond. Examination of the precedent for modulation of *C*_3_‐symmetric phenolic TACN ligands, reveals a scarcity of methods for selective mono‐functionalization, presumably due to the selectivity issues associated with stochastic approaches. Noteworthily, synthetic routes have been developed from mono‐*N*‐alkylated TACN derivatives yielding pentadentate *N*,*N*,*N*,*O*,*O* donor ligands.^[30]^ We envisaged a conceptually simple alternative approach in which [K(η^6^‐benzene)][(*t*‐BuTACN)Ca] (**2**) is O‐alkylated in situ by functionalization of one phenoxide arms (Scheme 2). We opted for a methylation strategy given its limited steric requirements and prevalence in organic synthesis.^[31]^ Accordingly, the reaction of **2** with excess iodomethane affords the neutral complex (*t*‐BuTACN−Me)Ca (**4**) in excellent yield (see Figure S34 for details of other functionalization attempts). NMR spectroscopy supports the formation of the target complex, with the two aromatic signals of *C*_3_‐symmetric **2** being split into six resonances, consistent with desymmetrization of the ligand scaffold in **4**. Single crystal X‐ray diffraction confirmed the solid‐state structure, with the single methylated O‐donor giving rise to the longest Ca–O bond of the series of complexes **2–4** (2.407(4) Å, cf. 2.189(4) and 2.214(5) Å for the anionic phenoxide donors).

**Scheme 2:**
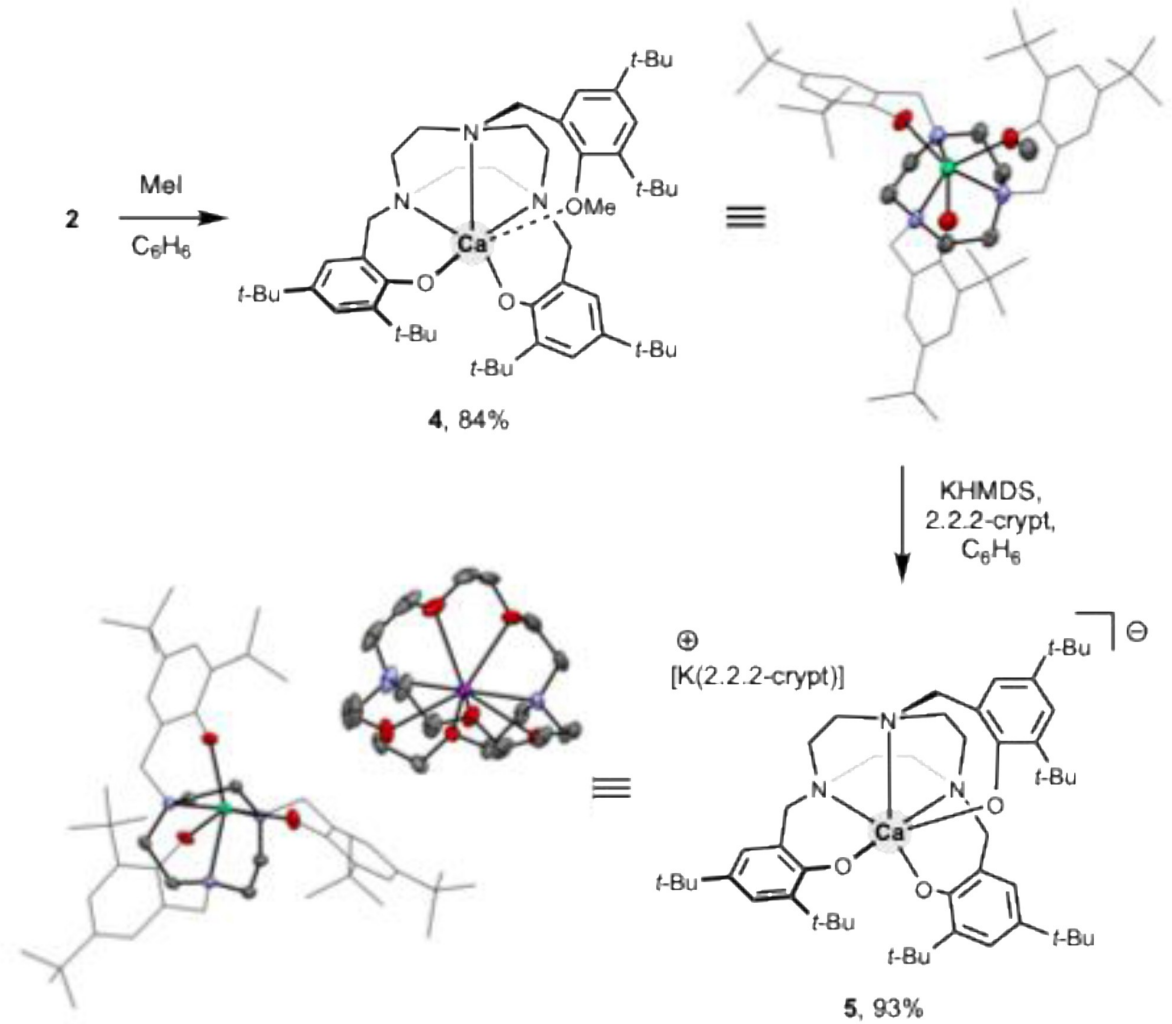
Synthesis and X‐ray structures of Ca complexes **4** and **5** (ellipsoids set at 50 % probability level; H atoms omitted and arenes shown in wireframe format for clarity).

With neutral complex **4** in hand, we evaluated its capabilities for fluoride uptake, on the basis of its likely higher Lewis acidity compared to anionic precursor **2**. In our previous work, we obtained [L_n_(X)_2_CaF]^−^ systems using a method initially described by Roesky et al., in which a calcium amido complex (either isolated or generated in situ*)* undergoes metathesis with Me_3_SnF to generate a Ca–F bond.^[18]^ In the case of **4**, no reaction is observed with K[HMDS]. Although the combination of K[HMDS]/2.2.2‐cryptand in benzene leads to the formation of a single new species, NMR spectroscopy shows the new complex to possess *C*_3_‐symmetry on the NMR timescale and single crystal X‐ray diffraction revealed it simply to be [K(2.2.2‐crypt)][(*t*‐BuTACN)Ca] (**5**) formed by nucleophilic demethylation.

To avoid complications arising from dealkylation, milder fluorination conditions were employed (Scheme 3A). Sonicating KF/2.2.2‐cryptand with **4** at 50 °C for 1 h led to a new major fluorine‐containing species with a ^19^F resonance at −89.4 ppm (THF‐*d*_8_). This shift is closely comparable to that measured for the previously reported dinuclear calcium monofluoride species [{(NON)Ca(THF)}_2_(μ‐F)]^−^ (−87.3 ppm in THF‐*d*_8_; Figure 1).^[25]^ Single crystals of the product were obtained by layering a benzene solution with hexane, albeit obtained in low yield (ca. 30 %; Scheme 3B). X‐ray crystallography indeed revealed the fluorine‐containing species to be a monofluoride bridged complex [{(*t*‐BuTACN−Me)Ca}_2_(μ‐F)]^−^ (**6**, as the [K(2.2.2‐crypt)]^+^ salt). The solid state structure of **6** shows a close‐to‐linear Ca–F−Ca unit (165.87(4)°) and statistically equal Ca–F bond lengths (2.176(9), 2.192(8) Å), being similar to those measured for [K(2.2.2‐crypt)][{(NON)Ca(THF)}_2_(μ‐F)] (166.9(1)° and 2.182(1)/2.187(1) Å).

**Scheme 3:**
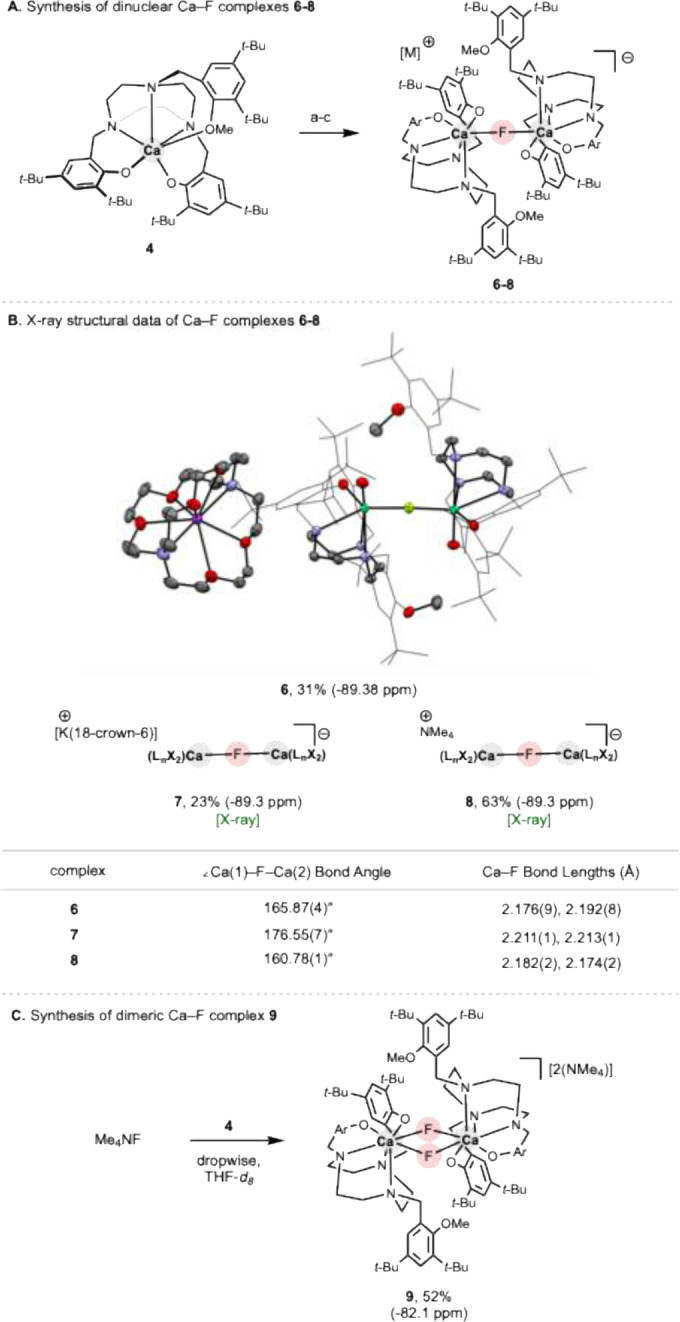
(A–C) Syntheses and X‐ray crystal structures of Ca–F complexes **6–8** (ellipsoids set at 50 % probability level; H atoms omitted and arenes shown in wireframe format for clarity). (a) KF, 2.2.2‐cryptand, and C_6_D_6_. (b) KF, 18‐crown‐6, and C_6_D_6_. (c) TMAF and C_6_D_6_/THF 4 : 1.

To improve the yield of isolable calcium‐fluoride complexes, **4** was also treated, in two separate reactions, with KF/18‐crown‐6 and TMAF. The structurally similar anionic dinuclear calcium monofluoride complexes [K(18‐crown‐6)][{(*t*‐BuTACN−Me)Ca}_2_(μ‐F)] (**7**) and [TMA][{(*t*‐BuTACN−Me)Ca}_2_(μ‐F)] (**8**) were obtained in yields of ca. 20 and 60 %, respectively. These complexes exhibit identical ^19^F NMR signals in THF‐*d*_8_ (δ_F_=‐89.3 ppm for each), and single crystals of both complexes suitable for X‐ray crystallography were obtained from toluene or toluene/THF (see Figures S39 and S40). The solid state structure of **8** resembles closely that of **6** (∠Ca–F–Ca=160.78(1)°, *d*(Ca–F)=2.182(2), 2.174(2) Å), while the anionic component of **7** features a more linear structural motif (176.6(1)°) and longer Ca–F bonds (2.211(1), 2.213(1) Å), presumably reflecting a relatively shallow potential energy surface for deformation about the (highly ionic) Ca–F−Ca unit.

In more general terms, the formation of bridged complexes **6–8** presumably reflects the fact that fluoride uptake at calcium is accompanied by immediate ‘capping’ by a second equivalent of neutral complex **4**, thereby leading to the formation of a product with a 2 : 1 Ca : F ratio. We attribute this to the low local concentration of the fluoride anion, due to the poor solubility of KF (in particular) in benzene compared to complex **4**.

With this in mind, we sought to increase the relative concentration of fluoride (with respect to Ca^2+^) by employing TMAF in THF instead of benzene. Therefore, a solution of **4** in THF was added dropwise to a pre‐sonicated and rapidly stirred THF‐solution of TMAF (2.0 equiv.). Under these conditions a single (different) fluorine‐containing species was observed, giving rise to a signal at δ_F_=−82.1 ppm. The downfield shift in the ^19^F signal (compared to those measured for compounds **6–8**) is of the same sense as that observed between single and doubly fluoride‐bridged NON ligated calcium fluoride complexes (e.g. δ_F_=−87.3 and −70.4 for [K(2.2.2‐crypt)][{(NON)Ca(THF)}_2_(μ‐F)] and [TMA]_2_[{(NON)Ca}_2_(μ‐F)_2_], respectively; Figure 1). The product, formulated as [TMA]_2_[{(*t*‐BuTACN−Me)Ca}_2_(μ‐F)_2_] (**9**) was isolated in ca. 50 % yield as a white solid, although X‐ray quality single crystals could not be obtained to allow for unequivocal structure determination by X‐ray crystallography (Scheme 3C). The ^1^H NMR spectrum of **9**, in line with the measured ^19^F chemical shift, is consistent with the presence of a 1 : 1 Ca : F ratio, on the basis of an equimolar ratio of the intensities of the [NMe_4_]^+^ and *t*‐BuTACN signals. We also carried out diffusion‐ordered spectroscopy (DOSY) ^19^F NMR measurements on complexes **7** and **9** in THF‐*d*_8_. The hydrodynamic radius estimated for complex **7** using the Stokes–Einstein equation (10.7 Å) is comparable to that of complex **9** (9.3 Å) suggesting that **9** is dimeric in the solution state, and that a monomeric species [TMA][{(*t*‐BuTACN−Me)Ca}(F)] is unlikely. Such a species is also viewed as being unlikely on the basis of the ^19^F shift measured for **9**, since terminal or close‐to‐terminal calcium fluoride complexes have been shown to give rise to significantly higher field signals (e.g. δ_F_=−97.8 ppm for [K(NON)Ca(THF)(F)]_2_). As such, while other isomeric forms of complex **9** are plausible, the available data and structural precedent points to a dimeric species containing a central Ca(μ‐F)_2_Ca unit.[^25^, ^32^]

The fluoride anion delivery capabilities of the newly synthesized [{(*t*‐BuTACN−Me)Ca}_2_(μ‐F)]^−^ systems have been evaluated under very mild conditions (room temperature, benzene solution; Scheme 4). These studies focused on crypt‐stabilized system [K(2.2.2‐crypt)][{(*t*‐BuTACN−Me)Ca}_2_(μ‐F)] (**6**) because of its facile synthesis and isolation compared to **7–9**. In particular, we wished to compare the capabilities of **6** in nucleophilic fluoride delivery to recently reported NON‐stabilized systems. The reaction with 1‐adamantanecarbonyl chloride in C_6_D_6_ at room temperature affords 1‐adamantane‐carbonyl fluoride (δ_F_=23.2 ppm) in the same yield as for [K(2.2.2‐crypt)][{(NON)Ca(THF)}_2_(μ‐F)] (both 58 %). In a similar yield, triphenylcarbenium tetrakis(pentafluorophenyl)borate ([Ph_3_C][B(C_6_F_5_)_4_]) also reacts with **6** in C_6_D_6_ at room temperature, in this case to generate a quaternary C−F centre (Ph_3_CF: δ_F_ = −125.6 ppm).

**Scheme 4:**
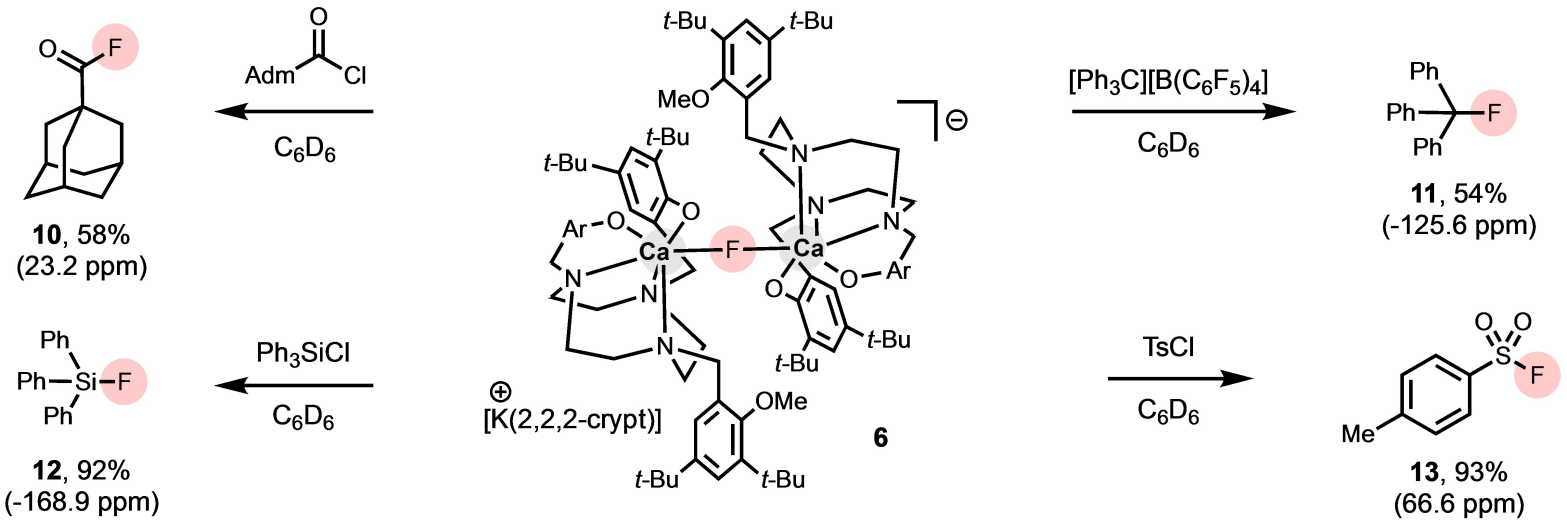
Reactivity of Ca–F complex **6** with organic electrophiles. Percentage yield by ^19^F NMR using fluorobenzene as internal standard.

With a view to probing if this reactivity could be expanded to construct other fluorine‐heteroatom bonds, silicon‐ and sulfur‐based electrophiles were also trialed. Treatment of complex **6** with chlorotriphenylsilane in C_6_D_6_ at room temperature generates fluorotriphenylsilane (δ_F_ = −168.9 ppm) in excellent (92 %) yield after 72 h, in contrast to [K(2.2.2‐crypt)][{(NON)Ca(THF)}_2_(μ‐F)], which plateaus at a more moderate yield (62 %) after ~15 min. Finally, we exposed complex **6** to tosyl chloride in C_6_D_6_ at room temperature, generating tosyl fluoride (δ_F_ = 66.6 ppm) in good/excellent yield after 30 min/72 h (68 %/93 %). The corresponding reaction with [K(2.2.2‐crypt)][{(NON)Ca(THF)}_2_(μ‐F)] gives only 17 % conversion to tosyl fluoride after 15 min, and does not improve with longer reaction times. It is worth noting that even the most effective NON‐derived system ([(NON)Ca(THF)(μ‐F)K(18‐crown‐6)]) affords only a 35 % yield of the S−F bonded product under comparable conditions (benzene, room temperature, 24 h).

For all of the NON‐systems, conversion of TsCl is rapid (with the same yields being obtained after 15 min and longer reaction times), and the low yield of the product is attributed to unwanted parallel tosylation reactivity involving the amide arms of the NON ligand. In the case of **6**, the aryloxide X‐donors of the TACN‐supported ligand are markedly less nucleophilic, thereby suppressing side reactivity and enhancing fluoride delivery. Consistently, all of the reactions of **6** carried out with electrophiles containing a chloride leaving group generate the same Ca‐containing product, identified as [{(*t*‐BuTACN−Me)Ca}_2_(μ‐Cl)]^−^. The ^1^H NMR spectra obtained in situ for all of these reactions match that measured for a sample of [K(2.2.2‐crypt)][{(*t*‐BuTACN−Me)Ca}_2_(μ‐Cl)], which was independently synthesized by reacting complex **4** with excess KCl and 2.2.2‐cryptand at 80 °C (see Figure S31 in Supporting Information for details).

In conclusion, we have synthesized and structurally characterized the first neutral and anionic TACN‐supported calcium complexes **2–3**, and developed a novel desymmetrization method using iodomethane as a methylating reagent to access the neutral L_4_X_2_‐supported complex (*t*‐BuTACN−Me)Ca, (**4**). This complex possesses sufficient fluorophilicity to take up fluoride in benzene or THF solution, generating a series of dinuclear anionic calcium monofluoride complexes **6–8**. The doubly bridged Ca_2_(μ‐F)_2_ complex **9** could also be synthesized by adding a dilute solution of complex **4** to a concentrated solution of TMAF in THF, and was characterized in solution by a range of NMR methods. Crucially, we show enhanced nucleophilic fluoride delivery for **6** over the NON‐derived counterparts with respect to Si‐ and S‐based electrophiles. O‐ligation appears to offer higher yields for these electrophiles, on the basis of less competitive side reactions, due to the less nucleophilic nature of the aryloxide X‐donors. As such, NMR spectroscopic evidence demonstrates that the fluoride anion from the Ca–F−Ca unit is replaced by the chloride anion from the electrophile in high yield. We believe that these findings will inform future work in designing soluble molecular Ca–F complexes, and thus further aid our fundamental understanding of nucleophilicity of the Ca–F bond.

## Conflict of Interests

The authors declare no conflict of interest.

## Supporting information

As a service to our authors and readers, this journal provides supporting information supplied by the authors. Such materials are peer reviewed and may be re‐organized for online delivery, but are not copy‐edited or typeset. Technical support issues arising from supporting information (other than missing files) should be addressed to the authors.

Supporting Information

## Data Availability

The data that support the findings of this study are available in the supplementary material of this article.
