# Supplementary material for: Nucleophilic Fluoride Anion Delivery from Triazacyclononane‐Supported Molecular Ca–F Complexes
Source: Angew Chem Int Ed Engl. 2024 Nov 9;64(2):e202414790. doi: 10.1002/anie.202414790 (PMC11720380; doi:10.1002/anie.202414790)
Supplement: Supplementary file 1 — Supporting Information [file ANIE-64-e202414790-s001.pdf]

Angewandte Chemie
Eine Zeitschrift der Gesellschaft Deutscher Chemiker

Supporting Information

Nucleophilic Fluoride Anion Delivery from Triazacyclononane-Supported Molecular Ca–F Complexes

O. Apolinar, J. J. C. Struijs, D. Sarkar, V. Gouverneur, S. Aldridge**

Supporting Information

©Wiley-VCH 2024

69451 Weinheim, Germany

Nucleophilic Fluoride Anion Delivery from Triazacyclononane-Supported Molecular Ca–F Complexes

Omar Apolinar, [a] Job J.C. Struijs, [a] Debotra Sarkar,[a] Véronique Gouverneur,*[a] and Simon Aldridge*[a]

DOI: 10.1002/anie.2024XXXXX

Table of Contents

Experimental Procedures 2
Preparation of *t*-BuTACN-H_3 ligand **1** 2
Preparation of [K(η^6-benzene)][(*t*-BuTACN)Ca] **2**....................7
Preparation of (*t*-BuTACN-H)Ca **3**....................10
Preparation of (*t*-BuTACN-Me)Ca **4** 13
Preparation of [K(2.2.2-cryptand)][(*t*-BuTACN)Ca] **5** 16
Preparation of [M][{(*t*-BuTACN-Me)Ca}$_2$F] **6-8**....................19
Preparation of [NMe_4][{(*t*-BuTACN-Me)CaF}$_2$] **9**....................30
^{19}F DOSY NMR Spectra of Compound **8** and **9** 34
Reactivity of [K(2.2.2-cryptand)][{(*t*-BuTACN-Me)Ca}$_2$F] **6** with electrophiles 36
Fluorination Outcomes of Complexes **2** and **3** 42
Unsuccessful Functionalizations of Complex **2** 43
Single Crystal X-ray Structure Determination.................... 44
References 52

Experimental Procedures

All manipulations were carried out using standard Schlenk line or dry-box techniques under an atmosphere of argon or dinitrogen. Solvents were degassed by sparging with argon and dried by passing through a column of the appropriate drying agent. NMR spectra were measured in C_6D_6 (which was dried over CaH_2) or THF-d_8 (which was dried over molecular sieves), with the solvent then being distilled under reduced pressure and stored under argon in Teflon valve ampoules. ^{1}H, ^{13}C and ^{19}F NMR spectra were recorded on 400 and 500 MHz Brucker spectrometers at ambient temperature and referenced internally to residual protio-solvent (^{1}H) or solvent (^{13}C) resonances and are reported relative to tetramethylsilane (δ = 0 ppm). Chemical shifts are quoted in δ (ppm) and coupling constants in Hz. Elemental analyses were carried out by London Metropolitan University. Both Me_3SnF[1] and $Ca(HMDS)_2(THF)_2$[2] were prepared by the literature methods.

***t*-BuTACN-H_3 protio-ligand 1**

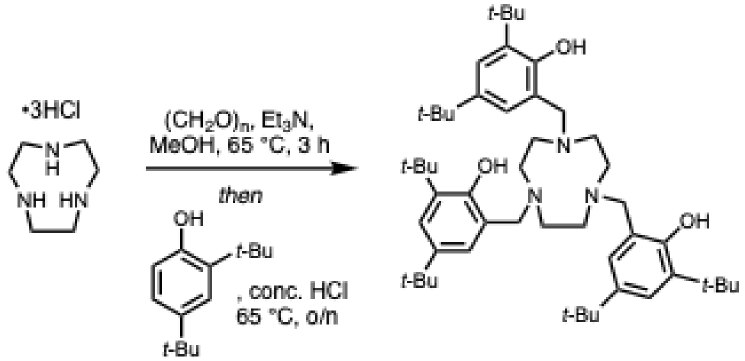

An adapted version of the literature procedure was used to synthesize the *t*-BuTACN-H_3 protio-ligand **1**.[3]

To a flame dried Schlenk flask equipped with a Teflon-coated magnetic stir bar was added anhydrous 1,4,7-triazacyclononane (TACN)·3HCl (1.00 g, 4.19 mmol) and paraformaldehyde (660 mg, 22.0 mmol). MeOH (0.4 M) and Et_3N (1.75 mL) were then added, and the reaction mixture stirred at 65 °C for 3 h. After this time, 2,4-di-*tert*-butylphenol (5.91 g, 25.2 mmol) was added to the orange solution which was allowed to completely dissolve before adding several drops of conc. HCl. The reaction mixture was stirred overnight at 65 °C after which time a white precipitate formed. The mother liquor was removed via filter cannula and the residual solid washed with MeCN (20 mL x 5). After drying under vacuum, the product was obtained as a white solid (2.72 g, 83% yield).

1,4,7-tris(3,5-tert-butyl-2-hydroxybenzyl)-1,4,7-triazacyclononane, (*t*-BuTACN-H_3), 1: ^{1}**H NMR** (400 MHz, $CDCl_3$) δ 10.23 (br s, 3H, PhO*H*), 7.22 (d, *J* = 2.4 Hz, 3H, Ph*H*), 6.77 (d, *J* = 2.3 Hz, 3H, Ph*H*), 3.73 (s, 6H, NC*H*$_2$Ph), 2.84 (s, 12H, NC*H*$_2$C*H*$_2$N), 1.43 (s, 27H, PhC(C*H*$_3$)$_3$), 1.25 (s, 27H, PhC(C*H*$_3$)$_3$); ^{13}**C{**^{1}**H} NMR** (100 MHz, $CDCl_3$) δ 153.9, 141.0, 135.8, 123.9, 123.4, 121.6 (*Ph*), 63.6 (N*C*H$_2$Ph), 55.4 (N*C*H$_2$*C*H$_2$N), 35.0, 34.3 (Ph*C*(CH$_3$)$_3$), 31.8, 29.8 (PhC(*C*H$_3$)$_3$). Characterization data in $CDCl_3$ match those reported in the literature.[3] ^{1}**H NMR** (400 MHz, C_6D_6) δ 10.43 (s, 3H), 7.51 (d, *J* = 2.4 Hz, 3H), 6.80 (d, *J* = 2.4 Hz, 3H), 3.32 (s, 6H), 2.43 (s, 12H), 1.73 (s, 27H), 1.34 (s, 27H). ^{13}**C{**^{1}**H} NMR** (100 MHz, C_6D_6) δ 154.6, 141.1, 136.1, 124.0, 123.5, 122.2 (*Ph*), 63.8 (N*C*H$_2$Ph), 55.7 (N*C*H$_2$*C*H$_2$N), 35.3, 34.4 (Ph*C*(CH$_3$)$_3$), 32.0, 30.0 (PhC(*C*H$_3$)$_3$).

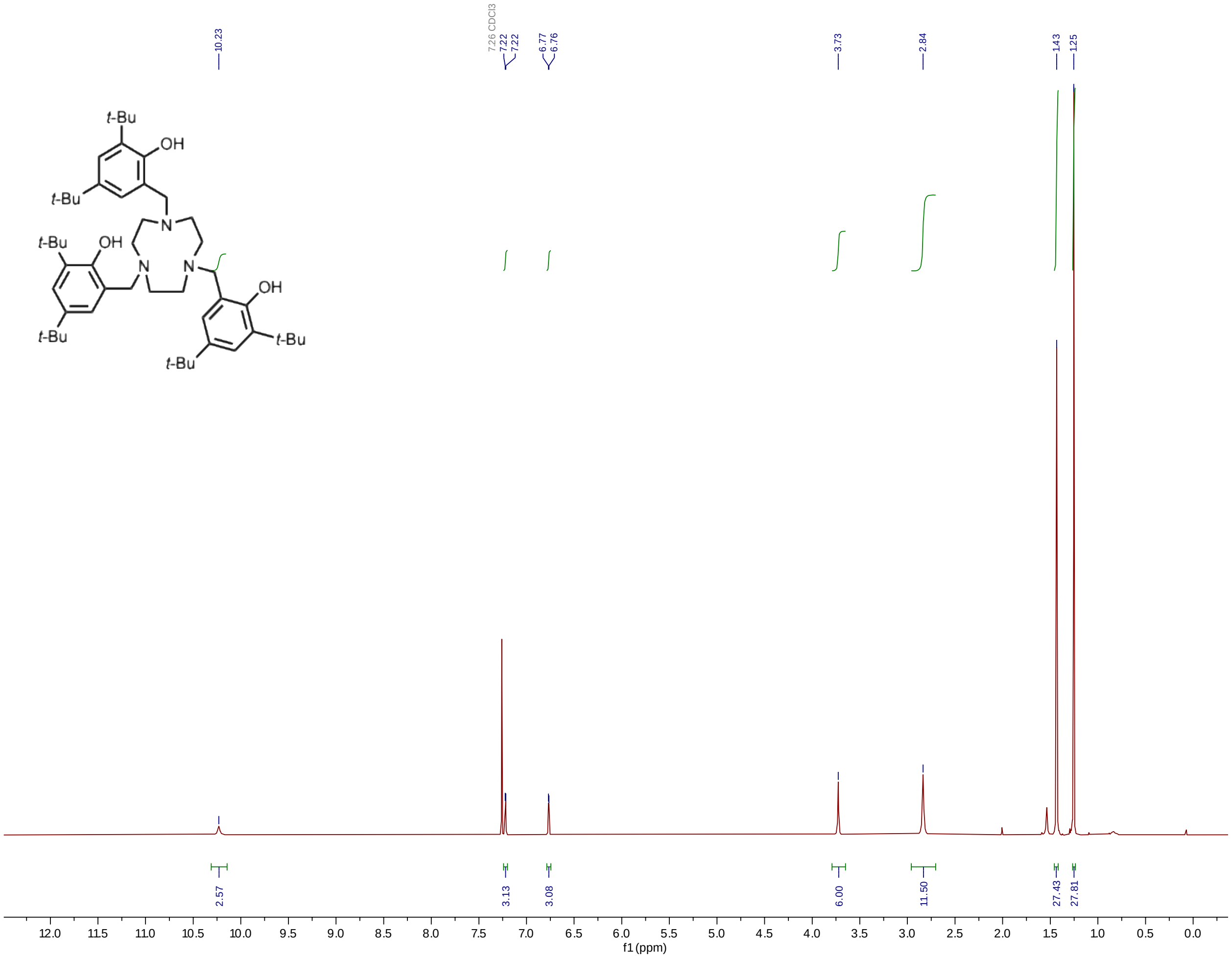

Figure S1. ^{1}H NMR spectrum of compound **1** in $CDCl_3$.

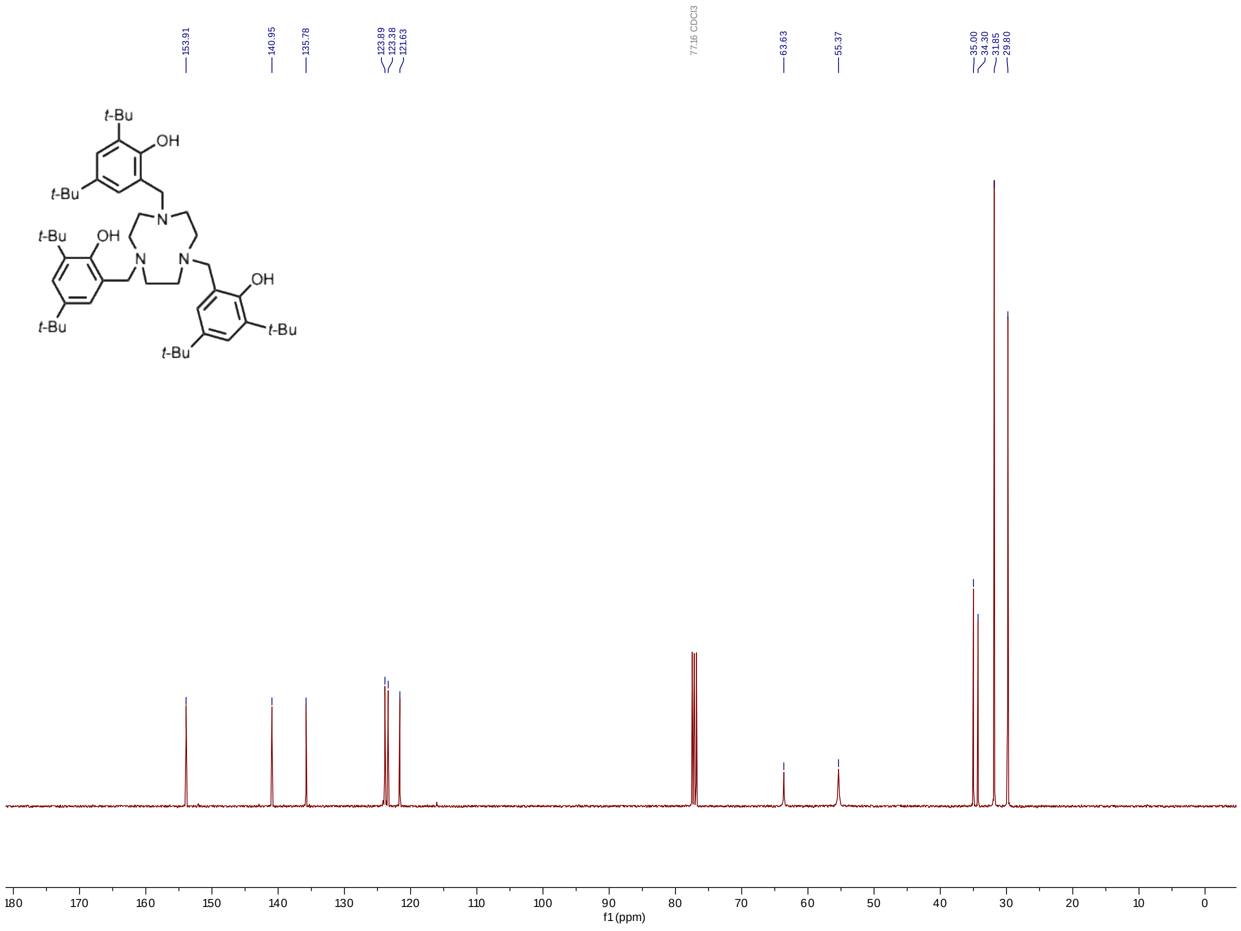

Figure S2. $^{13}C\{^{1}H\}$ NMR spectrum of compound **1** in $CDCl_3$.

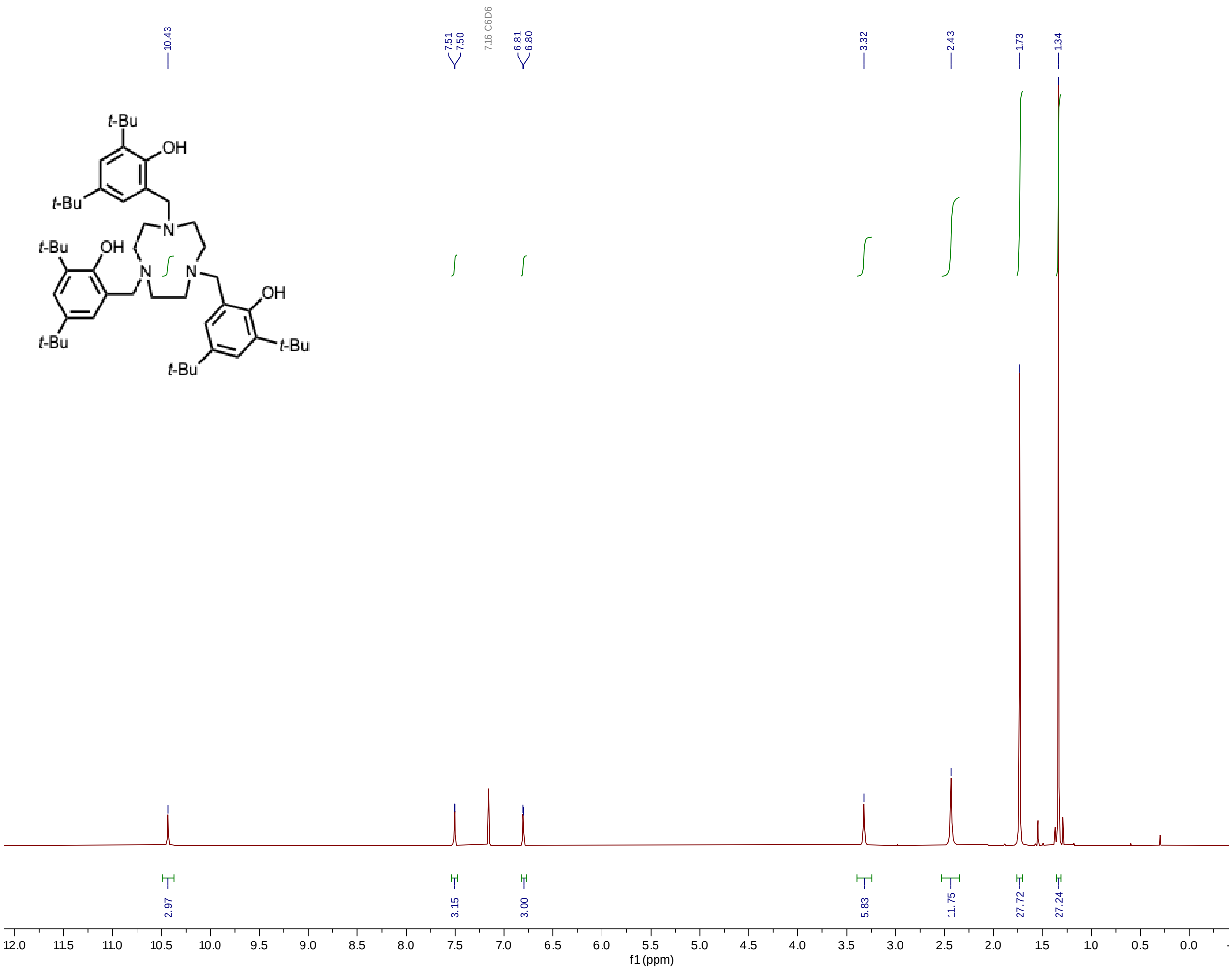

Figure S3. ^{1}H NMR spectrum of compound **1** in C_6D_6.

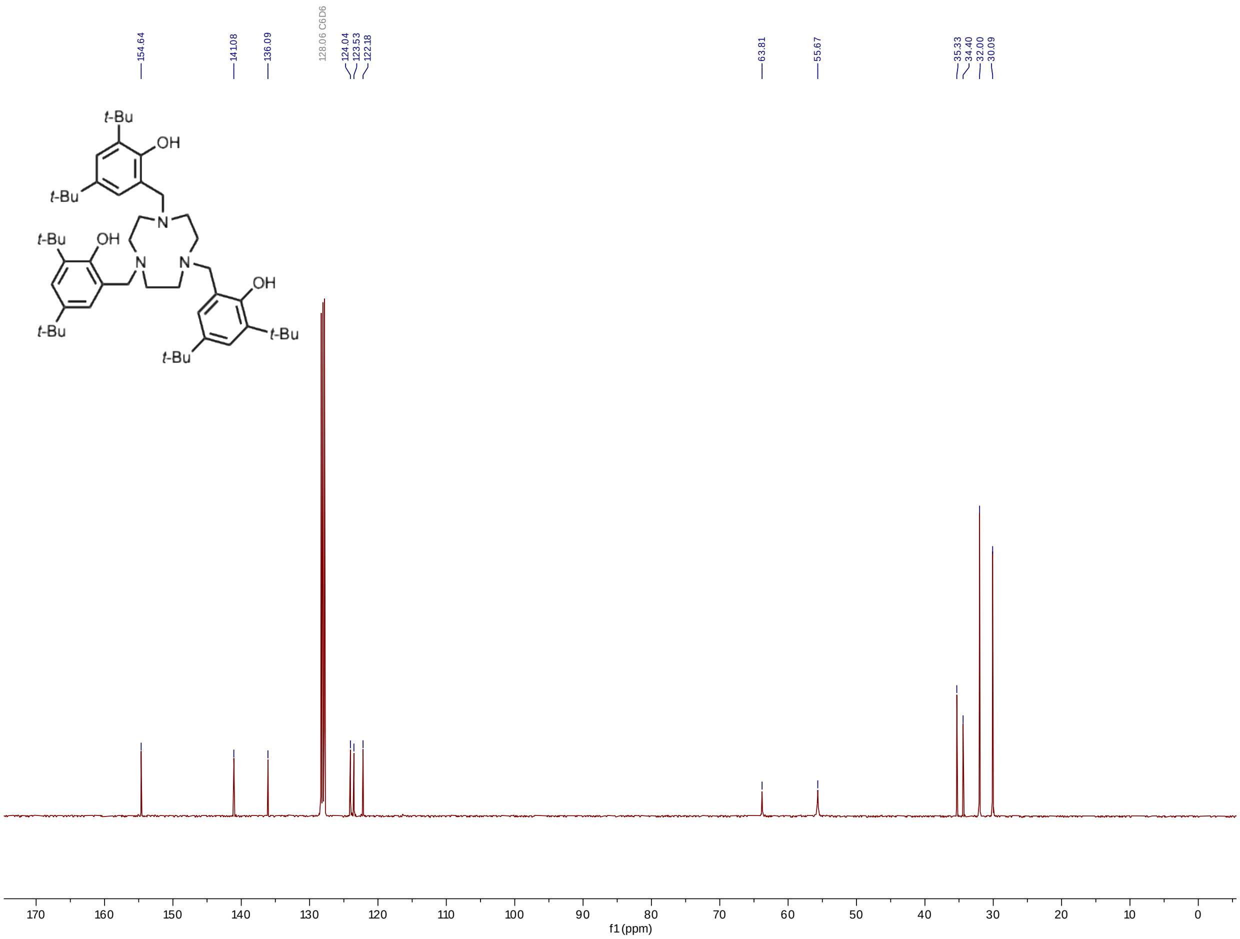

Figure S4. $^{13}C\{^{1}H\}$ NMR spectrum of compound **1** in C_6D_6.

Preparation of [K(η[6]-benzene)][(*t*-BuTACN)Ca], 2

To a flame dried Schlenk flask equipped with a Teflon-coated magnetic stir bar was added **1** (500 mg, 0.64 mmol), Ca(HMDS)$_2$(THF)$_2$ (322 mg, 0.64 mmol), and K[HMDS] (127 mg, 0.64 mmol) in an nitrogen filled glovebox. 12.5 mL of dry benzene was added via cannula and the reaction stirred at 80 °C overnight. After this time, the reaction mixture was transferred to another Schlenk flask by filter cannula and the precipitate further extracted with benzene (10 mL x 2). The combined benzene filtrate was concentrated under reduced pressure to ~10 mL and allowed to stand at room temperature. After 1 d, colorless crystals were observe to form; the mother liquor was removed by decantation to afford pure complex **2** as colorless crystals (260 mg, 47%).

For crystallization: To a J-Young NMR tube was added **1** (20.0 mg, 25.5 µmol), Ca(HMDS)$_2$(THF)$_2$ (12.9 mg, 25.5 µmol), K[HMDS (5.09 mg, 25.5 µmol) and 0.5 mL of C$_6$D$_6$. The reaction was allowed to proceed at room temperature, and after 1 d, colorless crystals of complex **2** were obtained, which were suitable for X-ray crystallography.

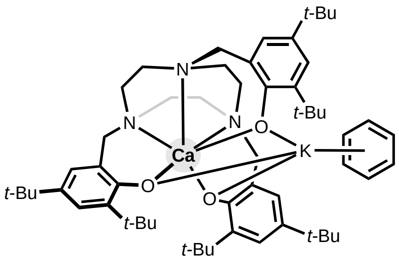

^{1}H NMR (400 MHz, C$_6$D$_6$) δ 7.56 (d, *J* = 2.7 Hz, 3H, Ph*H*), 6.99 (d, *J* = 2.6 Hz, 3H, Ph*H*), 3.96 (d, *J* = 11.1 Hz, 3H, NC*H*$_2$Ph), 2.78 (d, *J* = 11.1 Hz, 3H, NC*H*$_2$Ph), 2.44 (dd, *J* = 14.3, 4.1 Hz, 3H, NC*H*$_2$C*H*$_2$N), 2.19–1.95 (m, 6H, NC*H*$_2$C*H*$_2$N), 1.68 (d, *J* = 2.9 Hz, 3H, NC*H*$_2$C*H*$_2$N), 1.64 (s, 27H, PhC(C*H*$_3$)$_3$), 1.47 (s, 27H, PhC(C*H*$_3$)$_3$); **^{13}C{^{1}H} NMR** (100 MHz, C$_6$D$_6$) δ 163.8, 134.2, 131.9, 126.4, 123.2, 122.8 (*Ph*), 61.3 (N*C*H$_2$Ph), 56.0, 45.9 (N*C*H$_2$*C*H$_2$N), 33.9, 32.9 (Ph*C*(CH$_3$)$_3$), 31.2, 29.2 (PhC(*C*H$_3$)$_3$). Anal. Calcd. [%] for C$_{57}$H$_{84}$CaKN$_3$O$_3$: C, 72.95; H, 9.02; N, 4.48. Found: C, 73.62; H, 9.26; N, 4.36.

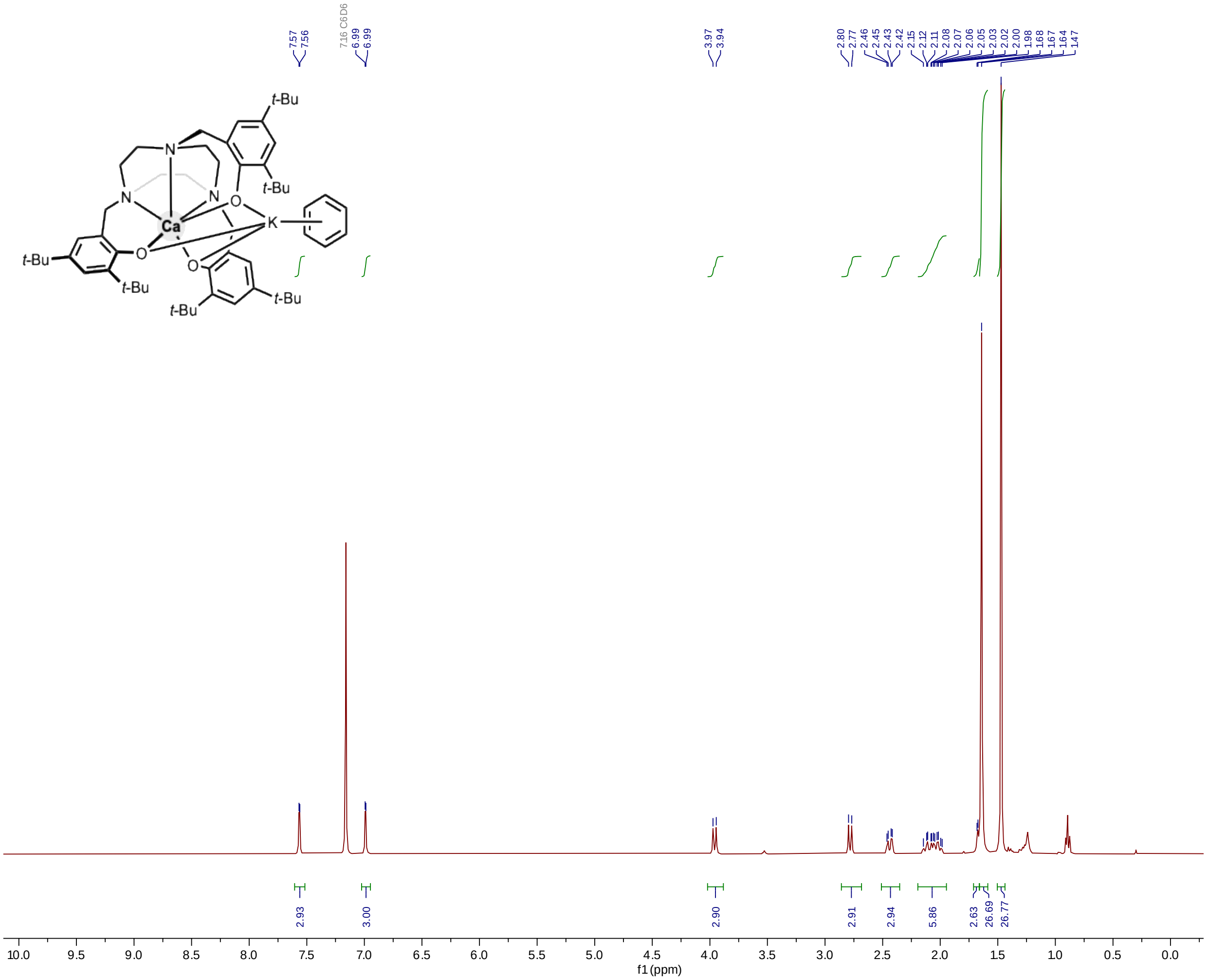

Figure S5. ^{1}H NMR spectrum of compound **2** in C_6D_6.

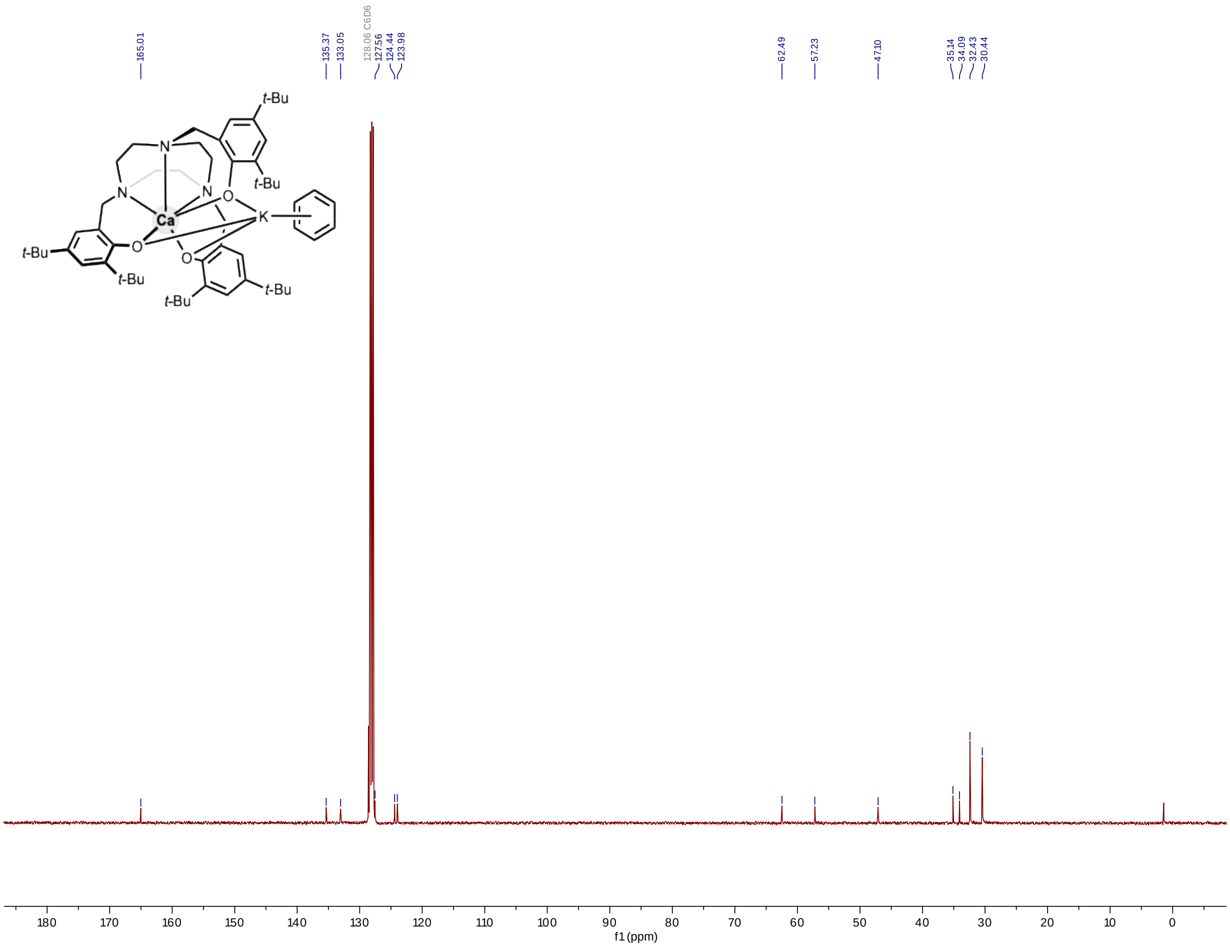

Figure S6. $^{13}C\{^{1}H\}$ NMR spectrum of compound **2** in C_6D_6.

Preparation of (*t*-BuTACN-H)Ca, 3

To a flame dried Schlenk flask equipped with a Teflon-coated magnetic stir bar was added **1** (500 mg, 0.64 mmol) and $Ca(HMDS)_2(THF)_2$ (322 mg, 0.64 mmol) in an nitrogen filled glovebox. 12.5 mL of dry benzene were added via cannula and the reaction stirred overnight in an oil bath at 80 °C. After this time, volatiles were removed under reduced pressure yielding a crude yellow oil. Purification by trituration with hot hexane (10 mL x 3) gave complex **2** as a white solid (176 mg, 34%).

For crystallization: To a J-Young NMR tube was added **1** (20.0 mg, 25.5 µmol), $Ca(HMDS)_2(THF)_2$ (12.9 mg, 25.50 µmol) and 0.5 mL of C_6D_6. The reaction was allowed to proceed until full conversion to **3** was observed by ^{1}H NMR spectroscopy. After this time, volatiles were removed under reduced pressure yielding a crude yellow oil to which was added hexane, and the concentrated solution immediately placed in an oil bath at 80 °C. After 1 d, colorless crystals of **3** were obtained, which were suitable for X-ray crystallography.

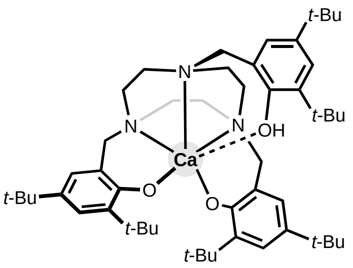

^{1}H NMR (400 MHz, C_6D_6) δ 7.55 (d, *J* = 2.1 Hz, 3H, Ph*H*), 7.39 (br s, 1H, PhO*H*), 6.94 (d, *J* = 2.3 Hz, 3H, Ph*H*), 3.96 (d, *J* = 11.6 Hz, 3H, NCH_2Ph), 2.77 (d, *J* = 11.6 Hz, 3H, NCH_2Ph), 2.30 (d, *J* = 14.2 Hz, 3H, NCH_2CH_2N), 2.14 (d, *J* = 13.3 Hz, 3H, NCH_2CH_2N), 2.06 (d, *J* = 14.5 Hz, 3H, NCH_2CH_2N), 1.72 (d, *J* = 2.0 Hz, 27H, PhC(CH_3)$_3$), 1.55 (d, *J* = 11.0 Hz, 3H, NCH_2CH_2N), 1.40 (d, *J* = 2.0 Hz, 27H, PhC(CH_3)$_3$); **^{13}C{^{1}H} NMR** (100 MHz, C_6D_6) δ 159.6, 136.8, 136.6, 126.5, 124.2, 123.7 (*Ph*), 62.8 (N*C*H$_2$Ph), 57.5, 47.2 (N*C*H$_2$*C*H$_2$N), 35.0, 33.9 (Ph*C*(CH$_3$)$_3$), 31.8, 30.3 (PhC(*C*H$_3$)$_3$. Anal. Calcd. [%] for $C_{51}H_{79}CaN_3O_3$: C, 74.49; H, 9.68; N, 5.11. Found: C, 74.37; H, 9.81; N, 4.94.

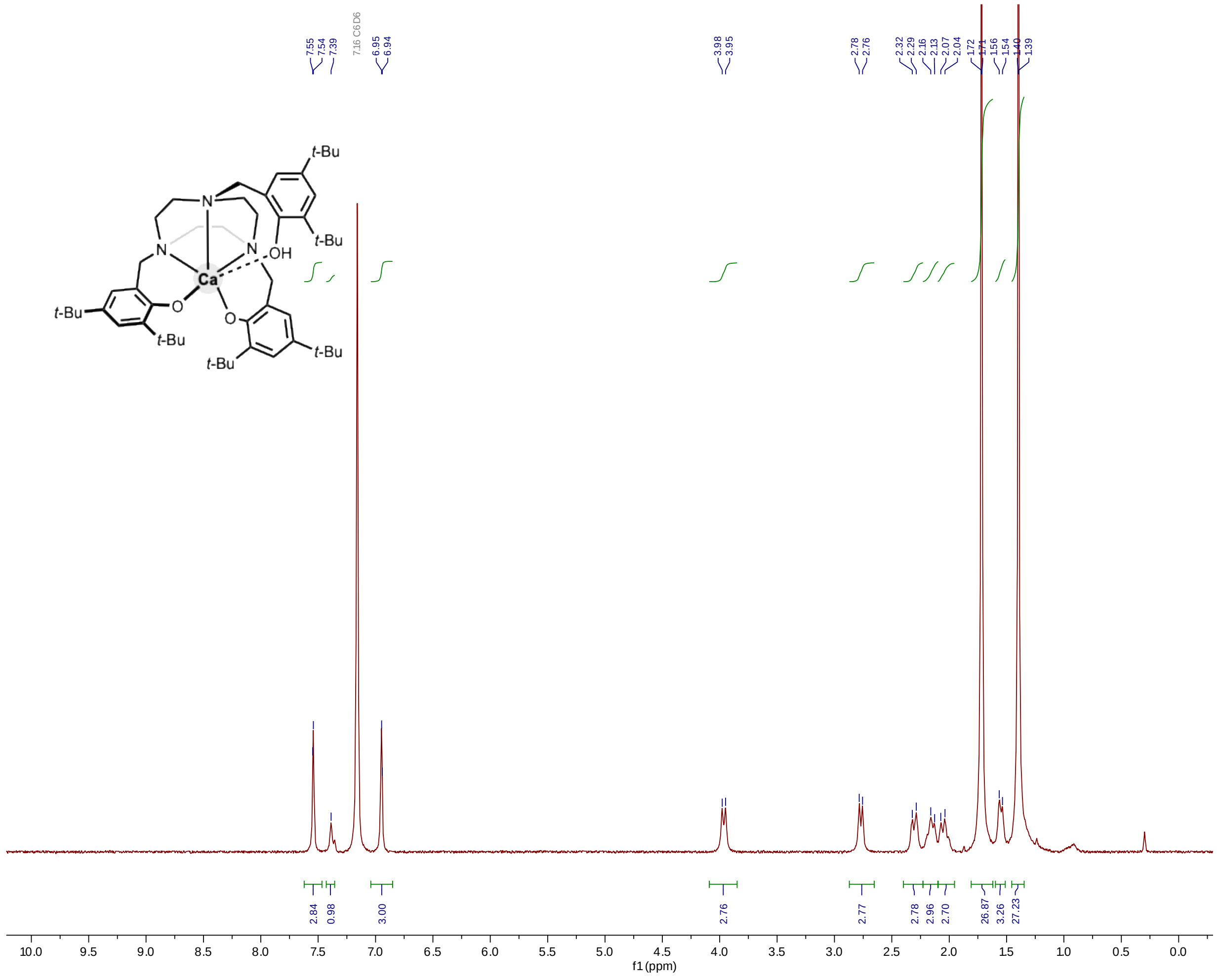

Figure S7. ^{1}H NMR spectrum of compound **3** in C_6D_6.

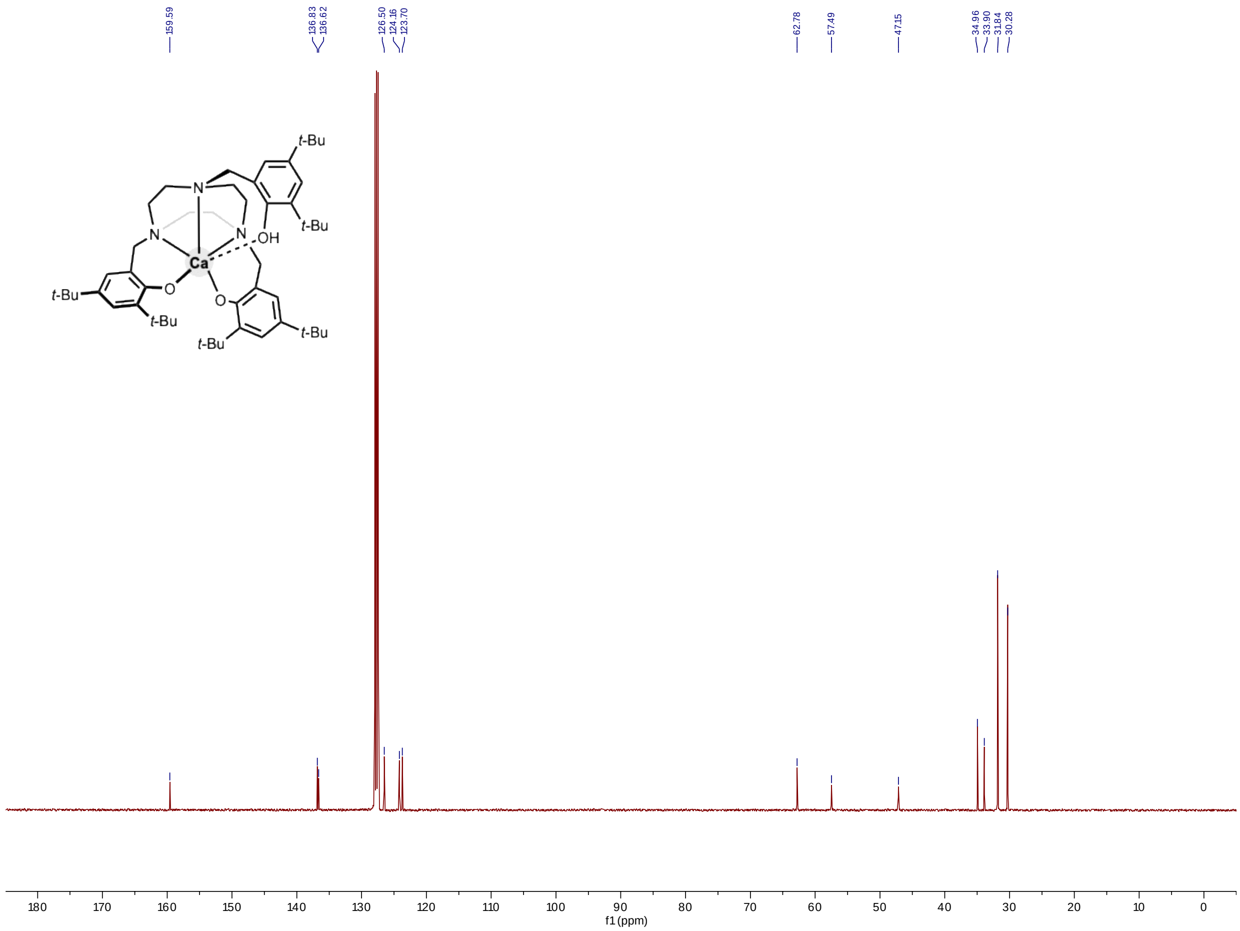

Figure S8. $^{13}C\{^1H\}$ NMR spectrum of compound **3** in C_6D_6.

Preparation of (*t*-BuTACN-Me)Ca 4

To a flame dried Schlenk flask equipped with a Teflon-coated magnetic stir bar was added **2** (2.0 g, mmol) in a nitrogen filled glovebox. 50 mL of dry benzene was added via cannula followed by 15 min of sonication and heating, causing the complex to partially dissolve. To the stirred solution was added dry iodomethane (0.6 mL, 4.5 equiv) via syringe and the reaction mixture stirred at 80 °C overnight. After this time, the reaction mixture was transferred into another Schlenk flask via filter cannula and all volatiles were removed under reduced pressure yielding an off white solid in sufficient purity for subsequent reactions (1.5 g, 84%).

For crystallization: **4** (100 mg, 120 µmol) and 1 mL of toluene were loaded into a lambda tube. Slow evaporation over a period of 5 d led to the formation of colorless crystals of **4**, which were suitable for X-ray crystallography.

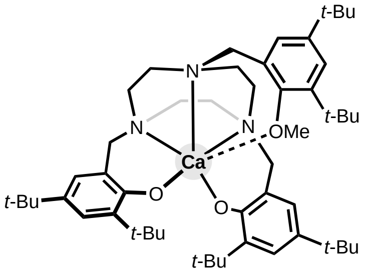

^{1}H NMR (400 MHz, C_6D_6) δ 7.65 (d, *J* = 2.7 Hz, 1H, Ph*H*), 7.60 (d, *J* = 2.7 Hz, 1H, Ph*H*), 7.47 (d, *J* = 2.5 Hz, 1H, Ph*H*), 7.05 (d, *J* = 2.7 Hz, 1H, Ph*H*), 7.03 (d, *J* = 2.7 Hz, 1H, Ph*H*), 6.75 (d, *J* = 2.5 Hz, 1H, Ph*H*), 4.14 (d, *J* = 12.1 Hz, 1H, NCH_2Ph), 4.03 (d, *J* = 11.6 Hz, 1H, NCH_2Ph), 3.96 (s, 3H, $PhOCH_3$), 3.82 (d, *J* = 10.9 Hz, 1H, NCH_2Ph), 2.96 (d, *J* = 12.2 Hz, 1H, NCH_2Ph), 2.80 (d, *J* = 10.9 Hz, 1H, NCH_2Ph), 2.72 (td, *J* = 12.6, 5.0 Hz, 1H, NCH_2CH_2N), 2.64–2.52 (m, 1H, NCH_2Ph; 1H, NCH_2CH_2N), 2.47 (dd, *J* = 14.8, 5.0 Hz, 1H, NCH_2CH_2N), 2.27 (td, *J* = 12.9, 5.1 Hz, 1H, NCH_2CH_2N), 2.13–1.90 (m, 3H, NCH_2CH_2N), 1.84 (d, *J* = 3.7 Hz, 18H, $PhC(CH_3)_3$), 1.79 (d, *J* = 4.8 Hz, 1H, NCH_2CH_2N), 1.60 (dd, *J* = 12.7, 3.7 Hz, 1H, NCH_2CH_2N), 1.48 (d, *J* = 6.0 Hz, 18H, $PhC(CH_3)_3$), 1.42 (s, 9H, $PhC(CH_3)_3$), 1.39 (d, *J* = 3.4 Hz, 1H, NCH_2CH_2N), 1.34 (d, *J* = 6.9 Hz, 1H, NCH_2CH_2N), 1.19 (s, 9H, $PhC(CH_3)_3$), 1.07 (td, *J* = 13.4, 5.2 Hz, 1H, NCH_2CH_2N); **^{13}C{^{1}H} NMR** (100 MHz, C_6D_6) δ 166.0, 165.9, 153.5, 148.5, 143.5, 137.1, 135.9, 132.6, 132.1, 131.0, 128.9, 127.2, 126.0, 124.2, 124.2, 123.9, 123.2 (*Ph*), 67.0 ($PhOCH_3$), 65.2, 64.6, 59.7 (NCH_2Ph), 57.8, 57.5, 57.5, 48.0, 47.6, 45.4 (NCH_2CH_2N), 36.3, 35.8, 35.7, 34.6, 34.1 ($PhC(CH_3)_3$), 32.9, 32.6, 32.5, 31.4, 30.6, 30.3 ($PhC(CH_3)_3$). Anal. Calcd. [%] for $C_{52}H_{81}CaN_3O_3$: C, 74.68; H, 9.76; N, 5.02. Found: C, 74.24; H, 9.90; N, 4.59.

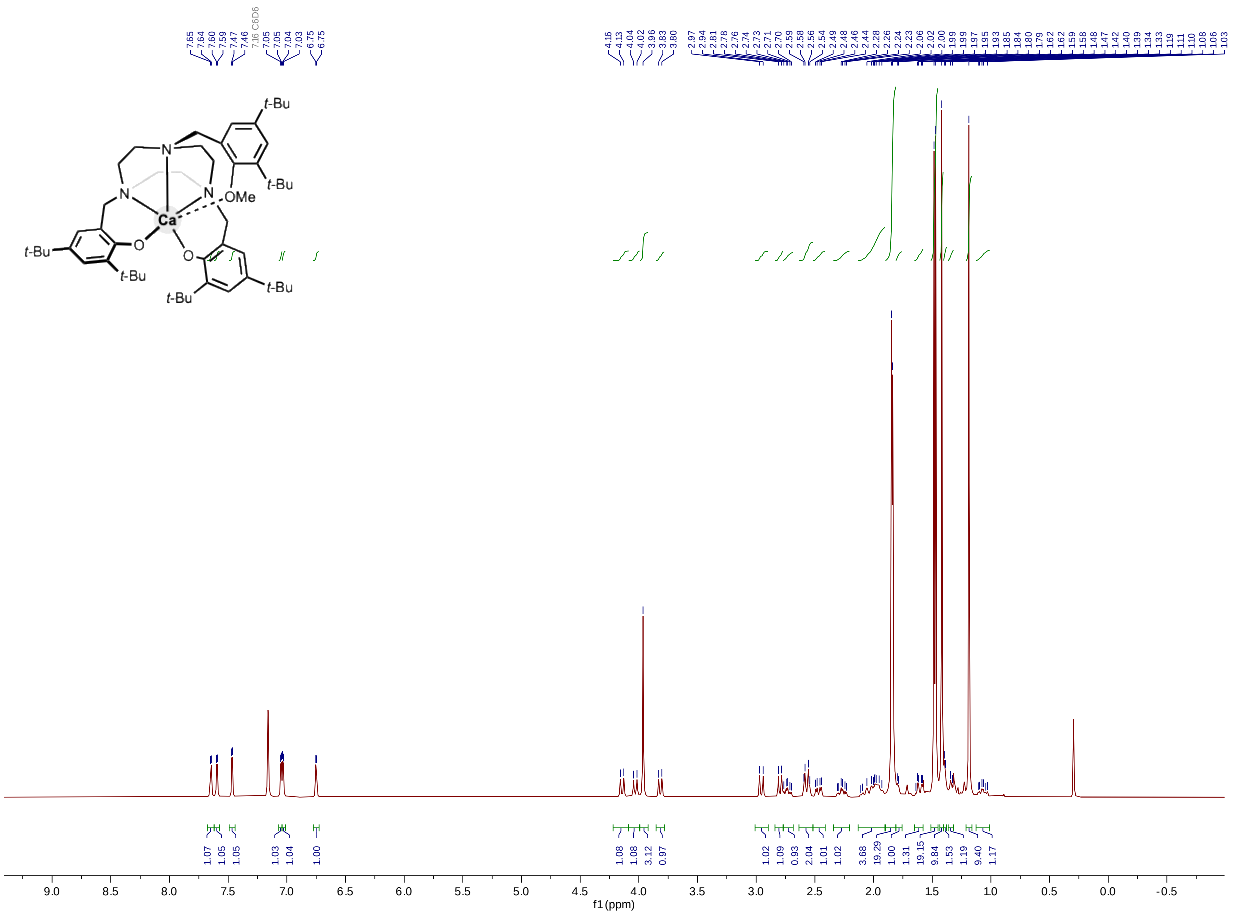

Figure S9. ^{1}H NMR spectrum of compound **4** in C_6D_6.

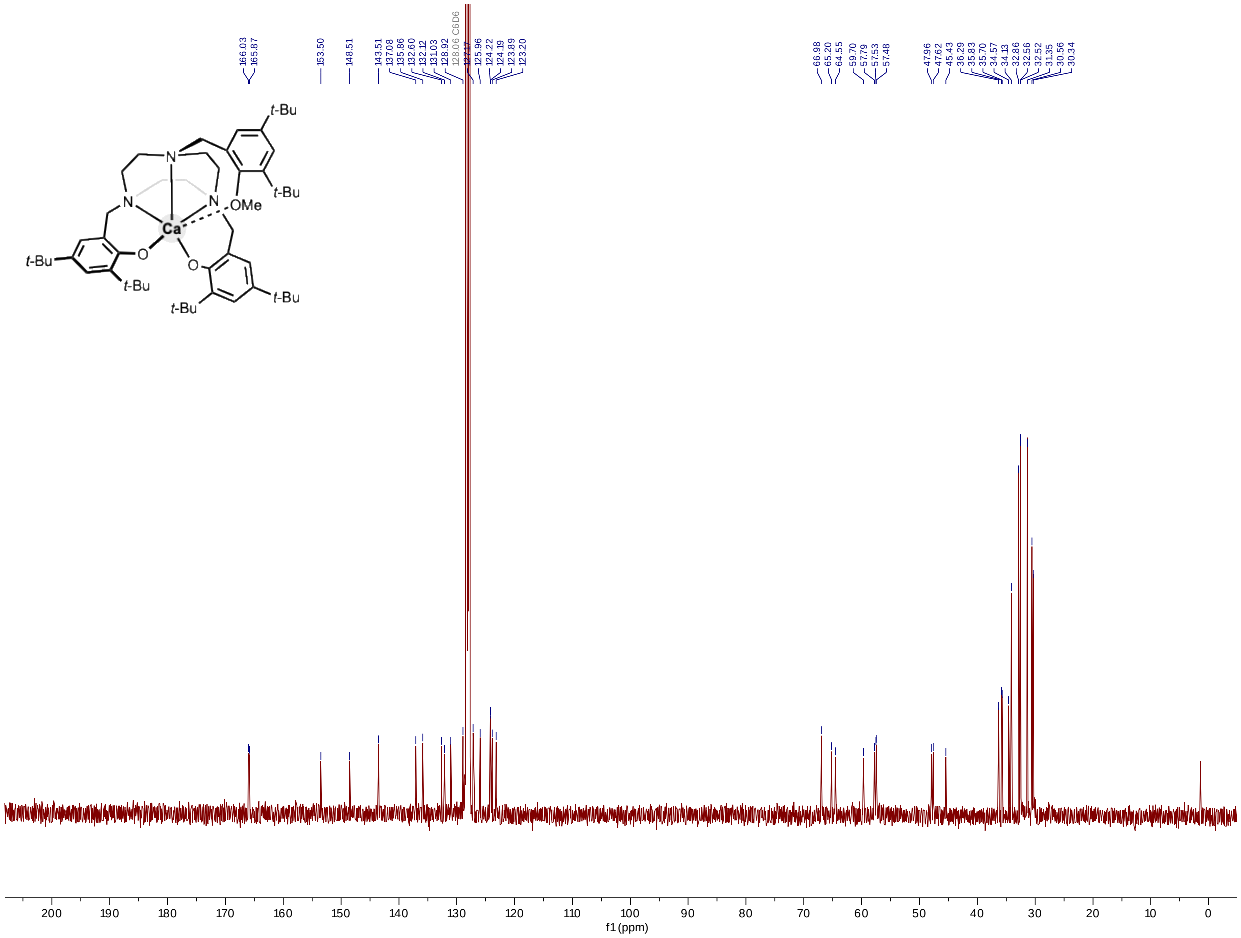

Figure S10. $^{13}C\{^{1}H\}$ NMR spectrum of compound **4** in C_6D_6.

Preparation of [K(2.2.2-crypt)][(t-BuTACN)Ca], 5

To a J-Young NMR tube was added **4** (50 mg, 60 µmol), K[HMDS] (12 mg, 60 µmol), 2.2.2-cryptand (22 mg, 60 µmol), and C_6D_6 in a nitrogen filled glovebox. The NMR tube was placed in an 80 °C oil bath for ca. 12 h. After this time, ^{1}H NMR spectroscopy revealed full consumption of the starting complex and the formation of a new symmetrical species. In a glovebox, the reaction mixture was filtered into a vial with a syringe and was layered with 1 mL of hexane. After 1 d, a white precipitate was observed to form, which after removal of the mother liquor and drying under reduced pressure afforded complex **5** as a white solid (69 mg, 93%).

For crystallization: To a J-Young NMR tube was added **5** (20 mg, 16 µmol) in a nitrogen filled glovebox; 0.5 mL of THF was added via syringe and the NMR tube placed in an oil bath at 80 °C until all of the solid had dissolved. The solvent was then removed under reduced pressure leaving a colorless oily residue. Inside the glovebox, 0.5 mL of toluene was added, and the resulting solution filtered into a vial by syringe. After 1 d, colorless crystals of complex **5** were obtained, which were suitable for X-ray crystallography.

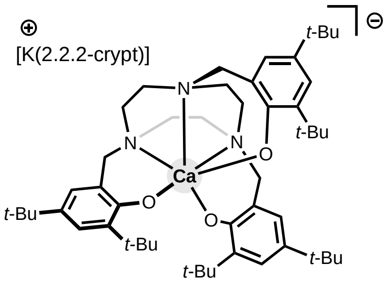

^{1}H NMR (400 MHz, THF-d_8) δ 7.00 (d, *J* = 2.8 Hz, 3H, Ph*H*), 6.68 (d, *J* = 2.7 Hz, 3H, Ph*H*), 4.04 (d, *J* = 10.6 Hz, 3H, NC*H*$_2$Ph), 3.56 (s, 12H, OC*H*$_2$C*H*$_2$O-crypt), 3.54–3.49 (m, 12H, OC*H*$_2$-crypt), 2.71 (d, *J* = 10.7 Hz, 3H, NC*H*$_2$Ph), 2.67–2.56 (m, 3H, NC*H*$_2$C*H*$_2$N-TACN), 2.56–2.46 (m, 16H {(12H, NC*H*$_2$-crypt) + (4H, NC*H*$_2$C*H*$_2$N-TACN)}), 2.31 (td, *J* = 12.5, 5.2 Hz, 3H, NC*H*$_2$C*H*$_2$N-TACN), 1.76 (d, *J* = 3.3 Hz, 2H, NC*H*$_2$C*H*$_2$N-TACN), 1.48 (s, 27H, PhC(C*H*$_3$)$_3$), 1.22 (s, 27H, PhC(C*H*$_3$)$_3$); **^{13}C{^{1}H} NMR** (100 MHz, THF-d_8) δ 168.2, 135.8, 129.2, 126.9, 125.1, 123.0 (*Ph*), 71.5 (O*C*H$_2$*C*H$_2$O-crypt), 68.6 (O*C*H$_2$-crypt), 65.2 (N*C*H$_2$Ph), 59.2 (N*C*H$_2$*C*H$_2$N-TACN), 54.9 (N*C*H$_2$-crypt), 47.9 (N*C*H$_2$*C*H$_2$N-TACN), 36.1, 34.4 (Ph*C*(CH$_3$)$_3$), 32.9, 31.05 (PhC(*C*H$_3$)$_3$). Anal. Calcd. [%] for $C_{72}H_{121}CaKN_5O_9$: C, 67.56; H, 9.53; N, 5.47. Found: C, 67.19; H, 8.94; N, 5.06.

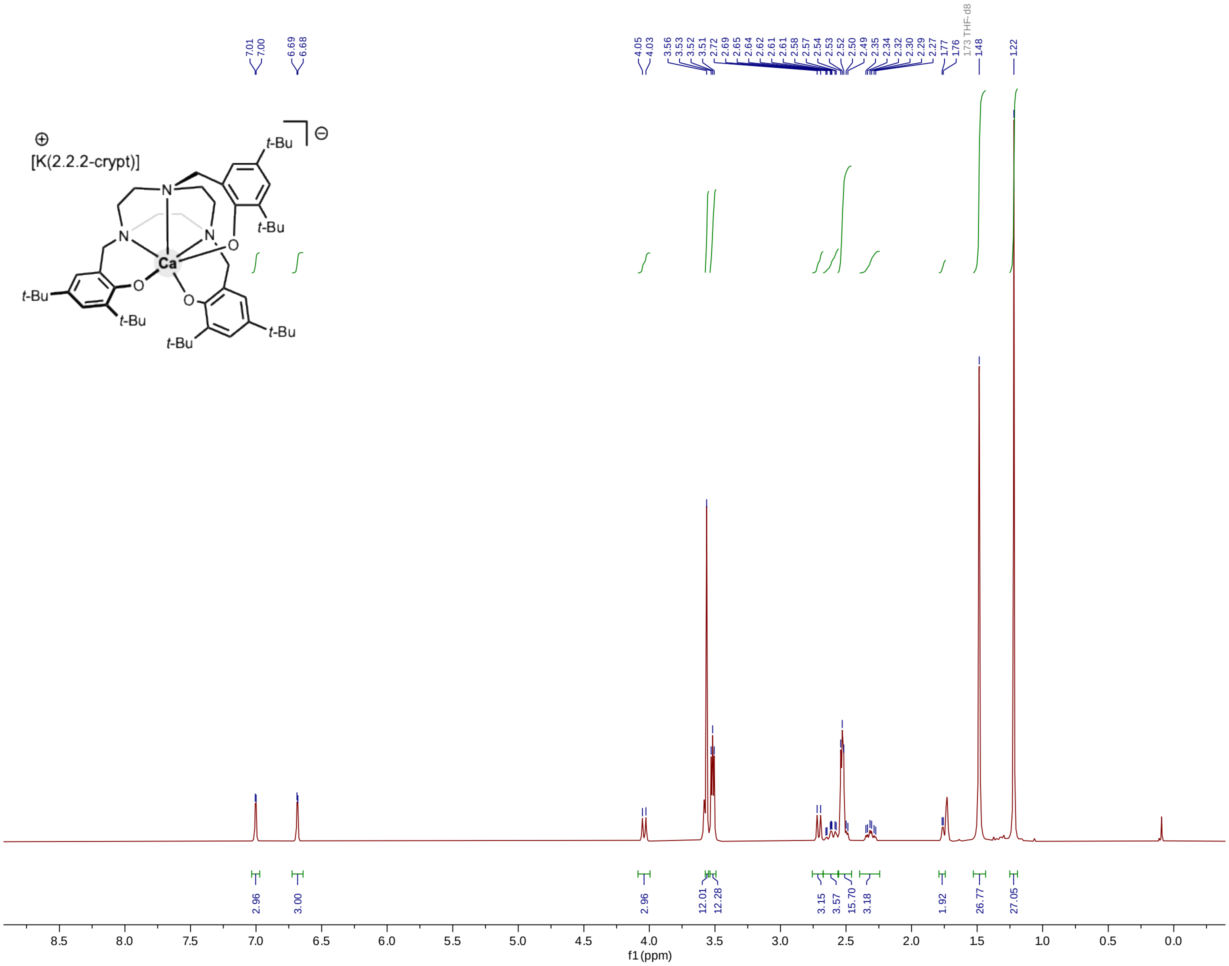

Figure S11. ^{1}H NMR spectrum of compound **5** in THF-d_8.

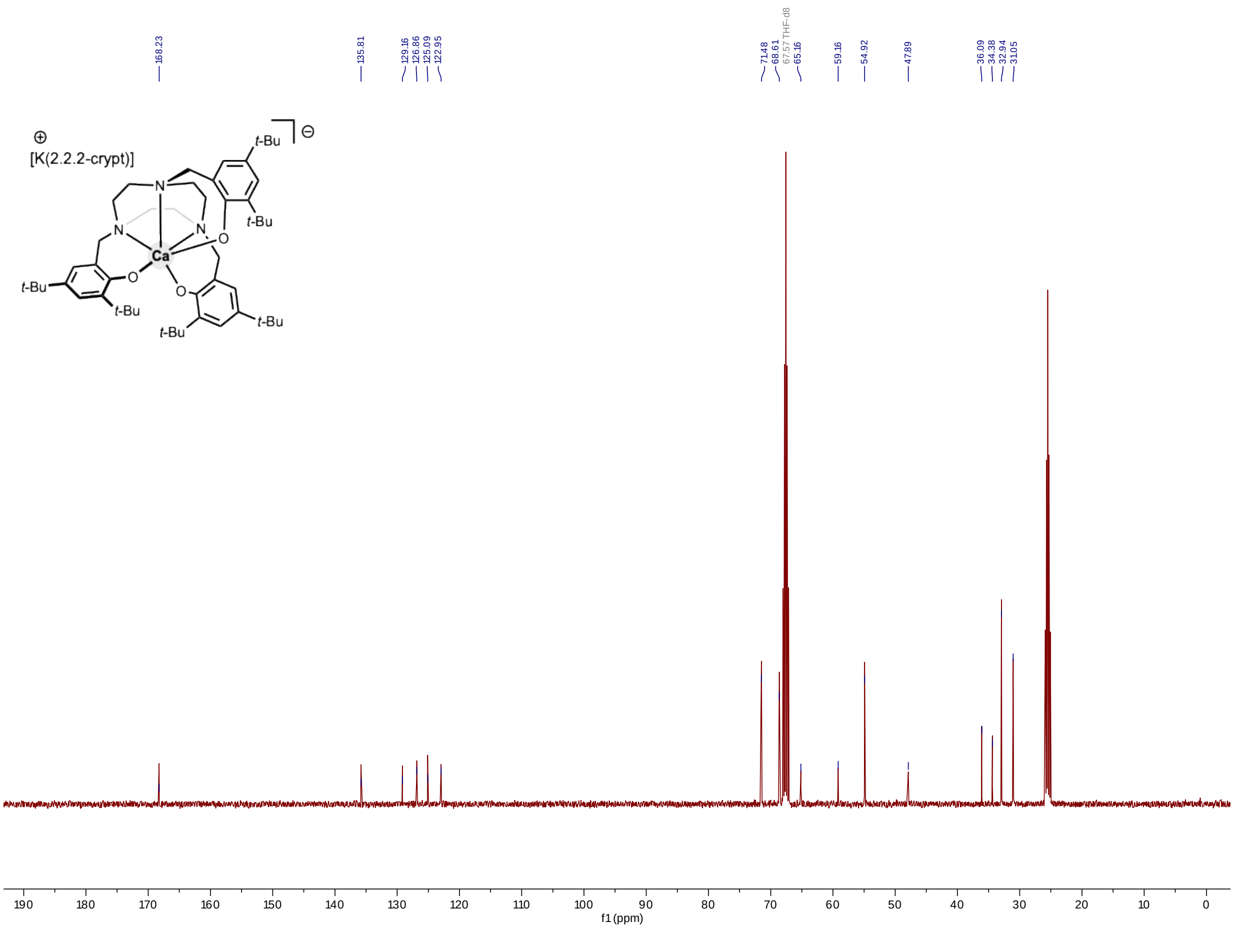

Figure S12. $^{13}C\{^{1}H\}$ NMR spectrum of compound **5** in THF-*d*8.

Preparation of [M][{(*t*-BuTACN)Ca}$_2$(μ-F)], [M]$^+$ = [K(2.2.2-crypt)]$^+$ (6), [K(18-crown-6)]$^+$ (7), [NMe$_4$]$^+$ (8)

General Procedure A: To a J-Young NMR tube was added **4**, the appropriate fluorinating agent, and C_6D_6 or toluene-*d*$_8$. The reaction mixture was sonicated for 30 min then placed in an oil bath at 80 °C until major conversion was observed. After this time, the reaction solution was filtered into a vial and allowed to settle or layered with hexane for crystallization. The mother liquor was decanted off and the resulting crystals washed with the mother solvent to afford pure colorless crystalline material of **6-8**.

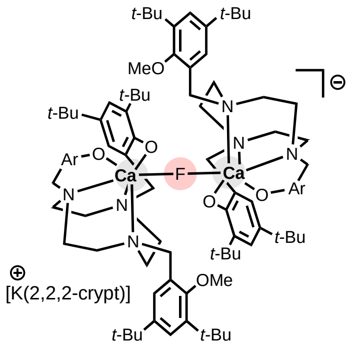

[K(2.2.2-crypt)][{(*t*-BuTACN)Ca}$_2$(μ-F)], 6: 6 was prepared from **4** (100 mg, 120 μmol), KF (17.4 mg, 299 μmol), 2.2.2-crypt (22.5 mg, 59.8 μmol), and C_6D_6 (0.5 mL) according to General Procedure A. Near full conversion was observed after 7 d, and the reaction mixture filtered into a vial and layered with hexane. After 1 d, the mother liquor was removed, and the remaining crystalline material washed with hexane (0.3 mL x 3), to afford after drying, complex **6** as a colorless crystalline solid (39 mg, 31%). Single crystals suitable for X-ray crystallography were obtained via the same crystallizing method. **^{1}H NMR** (500 MHz, THF-*d*$_8$) δ 7.16 (d, *J* = 2.5 Hz, 2H, Ph*H*), 7.04 (d, *J* = 2.7 Hz, 2H, Ph*H*), 7.00 (d, *J* = 2.7 Hz, 2H, Ph*H*), 6.87 (d, *J* = 2.6 Hz, 2H, Ph*H*), 6.64 (d, *J* = 2.8 Hz, 2H, Ph*H*), 6.62 (d, *J* = 2.8 Hz, 2H, Ph*H*), 5.08 (d, *J* = 14.8 Hz, 2H, NC*H*$_2$Ph), 4.45 (d, *J* = 12.0 Hz, 2H, NC*H*$_2$Ph), 4.27 (s, 6H, PhOC*H*$_3$), 4.02 (d, *J* = 10.3 Hz, 2H, NC*H*$_2$Ph), 3.97–3.82 (m, 4H, {(2H, NC*H*$_2$Ph) + (2H, NC*H*$_2$C*H*$_2$N)}), 3.58 (s, 12H, {(OC*H*$_2$C*H*$_2$O-crypt) + (overlap with THF-*d*$_8$ 3.58 peak)}, 3.53 (t, *J* = 4.7 Hz, 14H, {(12H, OC*H*$_2$-crypt) + (2H, NC*H*$_2$C*H*$_2$N)}), 2.71 (d, *J* = 11.9 Hz, 2H, NC*H*$_2$Ph), 2.66 (d, *J* = 10.4 Hz, 2H, NC*H*$_2$Ph), 2.55 (t, *J* = 4.7 Hz, 14H, {(12H, OC*H*$_2$-crypt) + (2H, NC*H*$_2$C*H*$_2$N)}), 2.52–2.43 (m, 4H, NC*H*$_2$C*H*$_2$N), 2.43–2.30 (m, 6H, NC*H*$_2$C*H*$_2$N), 2.05 (d, *J* = 13.0 Hz, 2H, NC*H*$_2$C*H*$_2$N), 1.89 (q, *J* = 10.5 Hz, 2H, NC*H*$_2$C*H*$_2$N), 1.71 (s, 2H, NC*H*$_2$C*H*$_2$N), 1.58 (s, 18H, PhC(C*H*$_3$)$_3$), 1.36 (s, 18H, PhC(C*H*$_3$)$_3$), 1.31 (d, *J* = 10.2 Hz, 2H, NC*H*$_2$C*H*$_2$N), 1.24 (s, 18H, PhC(C*H*$_3$)$_3$), 1.22 (s, 18H, PhC(C*H*$_3$)$_3$), 1.21 (s, 18H, PhC(C*H*$_3$)$_3$), 1.17 (s, 18H, PhC(C*H*$_3$)$_3$). **^{13}C{^{1}H} NMR** (126 MHz, THF) δ 168.2, 167.7, 158.1, 144.5, 142.6, 135.8, 135.6, 132.0, 130.3, 129.7, 129.7, 127.7, 127.2, 125.7, 125.2, 123.3, 123.2, 122.7 (*Ph*), 71.5 (O*C*H$_2$*C*H$_2$O-crypt), 68.6 (O*C*H$_2$-crypt), 65.0 (PhO*C*H$_3$), 64.8, 64.3 (N*C*H$_2$Ph), 58.7, 57.5 (N*C*H$_2$*C*H$_2$N), 57.0 (N*C*H$_2$Ph), 54.9 (N*C*H$_2$-crypt + overlap with N*C*H$_2$*C*H$_2$N-TACN peak), 48.0, 47.3, 47.1 (N*C*H$_2$*C*H$_2$N), 36.4, 36.0, 36.0, 35.1, 34.4, 34.3 (Ph*C*(CH$_3$)$_3$), 32.9, 32.8, 32.3, 31.9, 31.6, 31.2 (PhC(*C*H$_3$)$_3$). **^{19}F{^{1}H} NMR** (377 MHz, THF) δ -89.4.

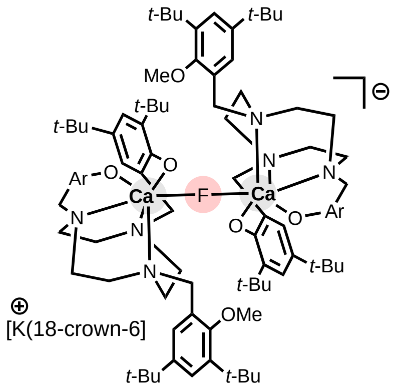

[K(18-crown-6)][{(*t*-BuTACN)Ca}$_2$(μ-F)], 7: 7 prepared from **4** (100 mg, 120 μmol), KF (17.4 mg, 299 μmol), 18-crown-6 (15.8 mg, 59.8 μmol), and toluene-*d*$_8$ (0.5 mL) according to General Procedure A. Near full conversion was observed after 21 d, and the reaction mixture was filtered into a vial and allowed to stand. After 1 d, the mother liquor was removed, and the remaining crystalline material was washed with toluene (0.3 mL x 3), to afford, after drying, complex **7** as a colorless crystalline solid (27 mg, 23%). Single crystals suitable for X-ray crystallography were obtained via the same crystallizing method. **^{1}H NMR** (500 MHz, THF) δ 7.16 (d, *J* = 2.5 Hz, 2H, Ph*H*), 7.04 (d, *J* = 2.8 Hz, 2H, Ph*H*), 7.00 (d, *J* = 2.8 Hz, 2H, Ph*H*), 6.88 (d, *J* = 2.6 Hz, 2H, Ph*H*), 6.65 (d, *J* = 2.8 Hz, 2H, Ph*H*), 6.62 (d, *J* = 2.7 Hz, 2H, Ph*H*), 5.08 (d, *J* = 14.9 Hz, 2H, NC*H*$_2$Ph), 4.45 (d, *J* = 11.9 Hz, 2H, NC*H*$_2$Ph), 4.27 (s, 6H, PhOC*H*$_3$), 4.02 (d, *J* = 10.2 Hz, 2H, NC*H*$_2$Ph), 3.98–3.83 (m, 4H, {(2H, NC*H*$_2$Ph) + (2H, NC*H*$_2$C*H*$_2$N)}), 3.59 (s, 24H, {(OC*H*$_2$C*H*$_2$O-18-crown-6) + (overlap with THF-*d*$_8$ 3.58 peak)}), 3.55 (dd, *J* = 15.4, 4.4 Hz, 2H, NC*H*$_2$C*H*$_2$N), 2.71 (d, *J* = 11.8 Hz, 2H, NC*H*$_2$Ph), 2.67 (d, *J* = 10.3 Hz, 2H, NC*H*$_2$Ph), 2.63–2.53 (m, 2H, NC*H*$_2$C*H*$_2$N), 2.50 (dd, *J* = 14.8, 4.6 Hz, 4H, NC*H*$_2$C*H*$_2$N), 2.44–2.33 (m, 6H, NC*H*$_2$C*H*$_2$N), 2.05 (dd, *J* = 13.1, 3.2 Hz, 2H, NC*H*$_2$C*H*$_2$N), 1.89 (td, *J* = 11.6, 7.5 Hz, 2H, NC*H*$_2$C*H*$_2$N), 1.70 (d, *J* = 3.5 Hz, 2H, NC*H*$_2$C*H*$_2$N), 1.58 (s, 18H, PhC(C*H*$_3$)$_3$), 1.36 (s, 18H, PhC(C*H*$_3$)$_3$), 1.34 (d, *J* = 2.7 Hz, 2H, NC*H*$_2$C*H*$_2$N), 1.24 (s, 18H, PhC(C*H*$_3$)$_3$), 1.22 (s, 18H, PhC(C*H*$_3$)$_3$), 1.215 (s, 18H, PhC(C*H*$_3$)$_3$), 1.17 (s, 18H, PhC(C*H*$_3$)$_3$). **^{13}C{^{1}H} NMR** (126 MHz, THF) δ 168.2, 167.7, 158.1, 144.6, 142.6, 135.8, 135.6, 132.1, 130.2, 129.8, 129.7, 127.6, 127.2, 125.7, 125.2, 123.3, 123.2, 122.7 (*Ph*), 71.3 (O*C*H$_2$*C*H$_2$O-18-crown-6), 65.0 (PhO*C*H$_3$), 64.8 (N*C*H$_2$Ph), 64.3 (N*C*H$_2$Ph), 58.7 (N*C*H$_2$*C*H$_2$N), 57.5 (N*C*H$_2$*C*H$_2$N), 57.0 (N*C*H$_2$Ph), 55.0 (N*C*H$_2$*C*H$_2$N), 48.0 (N*C*H$_2$*C*H$_2$N), 47.3 (N*C*H$_2$*C*H$_2$N), 47.1 (N*C*H$_2$*C*H$_2$N), 36.4, 36.0, 36.0, 35.1, 34.4, 34.3 (Ph*C*(CH$_3$)$_3$), 32.9, 32.8, 32.3, 31.9, 31.6, 31.2 (PhC(*C*H$_3$)$_3$). **^{19}F{^{1}H} NMR** (377 MHz, THF) δ -89.3. Anal. Calcd. [%] for $C_{116}H_{186}Ca_2FKN_6O_{12}$: C, 69.84; H, 9.40; N, 4.21. Found: C, 70.08; H, 9.10; N, 3.96.

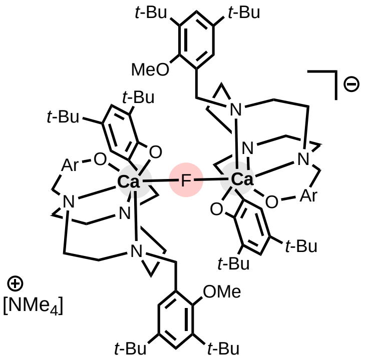

[NMe$_4$][{(*t*-BuTACN)Ca}$_2$(μ-F)] 8: 8 was prepared from **4** (50 mg, 60 µmol), TMAF (6.0 mg, 64 µmol) and C_6D_6/THF 4:1 solvent mixture (0.5 mL) according to General Procedure A. Near full conversion was observed after 12 h, and the reaction mixture was filtered into a vial and allowed to stand. After 1 d, the mother liquor was removed, the remaining crystalline material was washed with hexane (0.3 mL x 3), and after drying afforded complex **8** as a colorless crystalline solid (33 mg, 63%). Single crystals suitable for X-ray crystallography were obtained by slow cooling a reaction mixture of the same scale in a J-Young NMR tube, but with toluene-d_8/THF 4:1 as the crystallizing solvent system. **^{1}H NMR** (500 MHz, THF) δ 7.17 (d, J = 2.5 Hz, 2H, *Ph*), 7.04 (d, J = 2.7 Hz, 2H, *Ph*), 7.02 (d, J = 2.7 Hz, 2H, *Ph*), 6.87 (d, J = 2.5 Hz, 2H, *Ph*), 6.65 (d, J = 2.7 Hz, 4H, *Ph*), 5.07 (d, J = 14.8 Hz, 2H, NCH_2Ph), 4.44 (d, J = 12.0 Hz, 2H, NCH_2Ph), 4.25 (s, 6H, PhOCH_3), 4.00 (d, J = 10.3 Hz, 2H, NCH_2Ph), 3.95–3.83 (m, 4H, {(2H, NCH_2Ph) + (2H, NCH_2CH_2N)}), 3.57–3.50 (m, 2H, NCH_2CH_2N), 3.00 (s, 12H, N(CH_3)$_4^+$, 2.71 (d, J = 11.9 Hz, 2H, NCH_2Ph), 2.68 (d, J = 10.3 Hz, 2H, NCH_2Ph), 2.58 (dd, J = 15.5, 11.9 Hz, 2H, NCH_2CH_2N), 2.54–2.44 (m, 6H, NCH_2CH_2N), 2.40 (d, J = 8.2 Hz, 4H, NCH_2CH_2N), 2.38–2.30 (m, 2H, NCH_2CH_2N), 2.09–2.00 (m, 2H, NCH_2CH_2N), 1.86 (q, J = 11.6 Hz, 2H, NCH_2CH_2N), 1.58 (s, 18H, PhC(CH_3)$_3$), 1.36 (s, 20H, {18H, (PhC(CH_3)$_3$) + 2H, NCH_2C*H*}) , 1.24 (s, 18H, PhC(CH_3)$_3$), 1.22 (s, 18H, PhC(CH_3)$_3$), 1.21 (s, 18H, PhC(CH_3)$_3$), 1.17 (s, 18H, PhC(CH_3)$_3$). **^{13}C{^{1}H} NMR** (126 MHz, THF) δ 168.2, 167.7, 158.0, 144.6, 142.7, 135.9, 135.5, 132.0, 130.2, 130.0, 129.8, 127.7, 127.3, 125.7, 125.2, 123.4, 122.7 (*Ph*), 64.9 (PhO*C*H$_3$), 64.8 (N*C*H$_2$Ph), 64.2 (N*C*H$_2$Ph), 58.7 (N*C*H$_2$*C*H$_2$N), 57.5 (N*C*H$_2$*C*H$_2$N), 57.0 (N*C*H$_2$Ph), 55.9 (N(*C*H$_3$)$_4$), 55.0, 48.0, 47.3, 47.0 (N*C*H$_2$*C*H$_2$N), 36.4, 36.1, 36.0, 35.1, 34.4, 34.3 (Ph*C*(CH$_3$)$_3$), 32.9, 32.8, 32.3, 31.9, 31.6, 31.2 (PhC(*C*H$_3$)$_3$). **^{19}F{^{1}H} NMR** (377 MHz, THF) δ -89.3. Anal. Calcd. [%] for $C_{108}H_{174}Ca_2FN_7O_6$: C, 73.46; H, 9.93; N, 5.55. Found: C, 73.97; H, 9.74; N, 4.68.

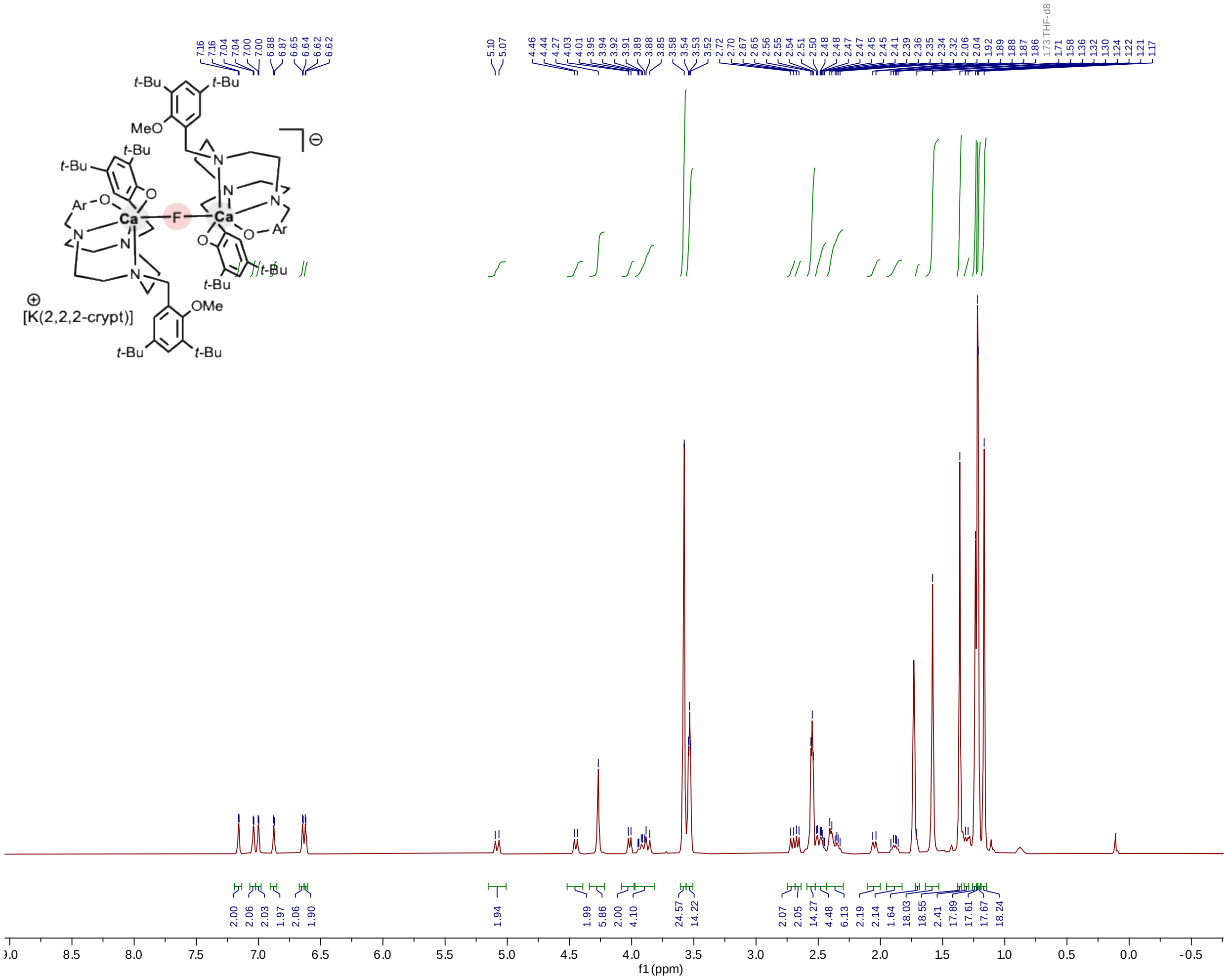

Figure S13. ^{1}H NMR spectrum of compound **6** in THF-d_8.

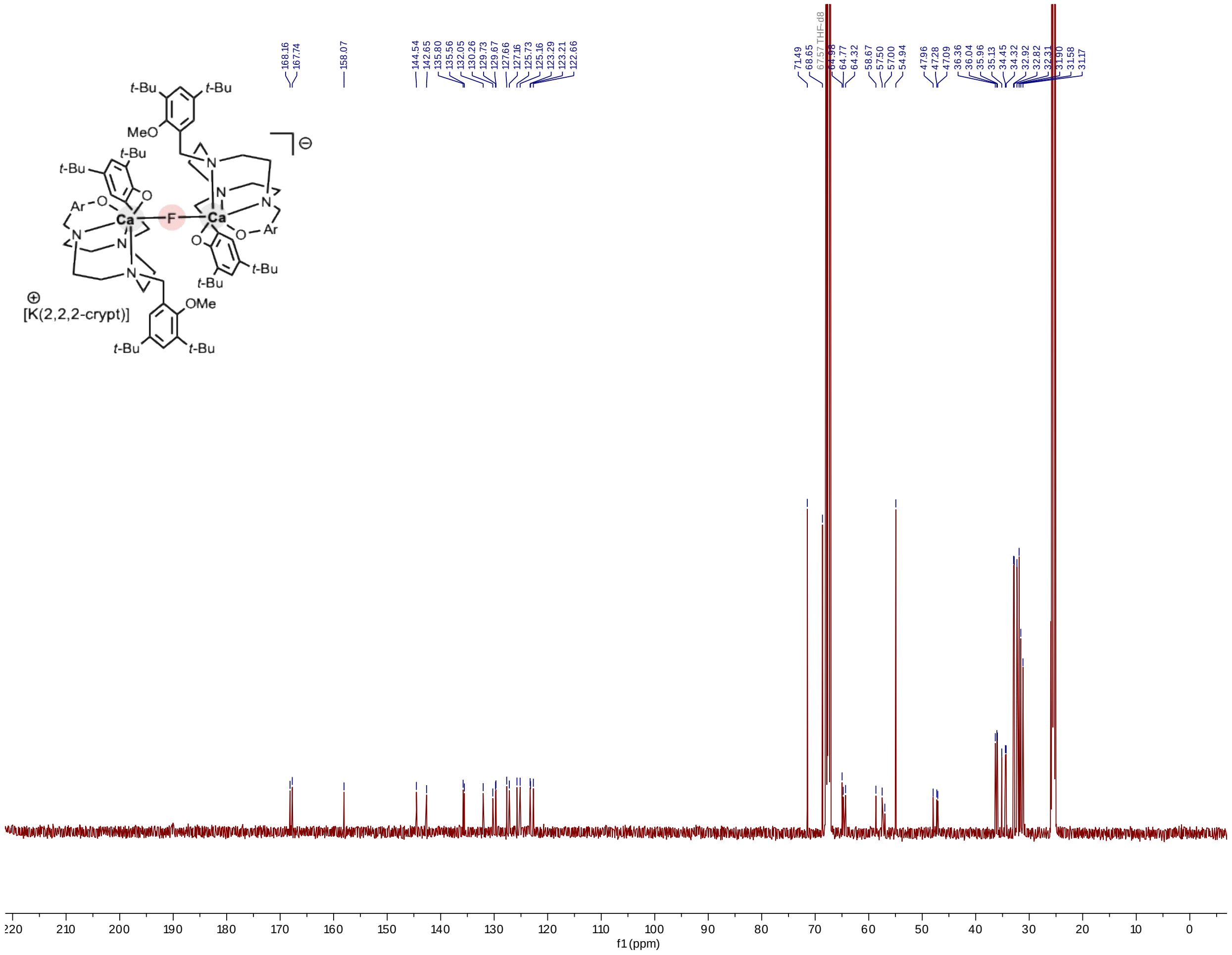

Figure S14. $^{13}C\{^{1}H\}$ NMR spectrum of compound **6** in THF-d_8.

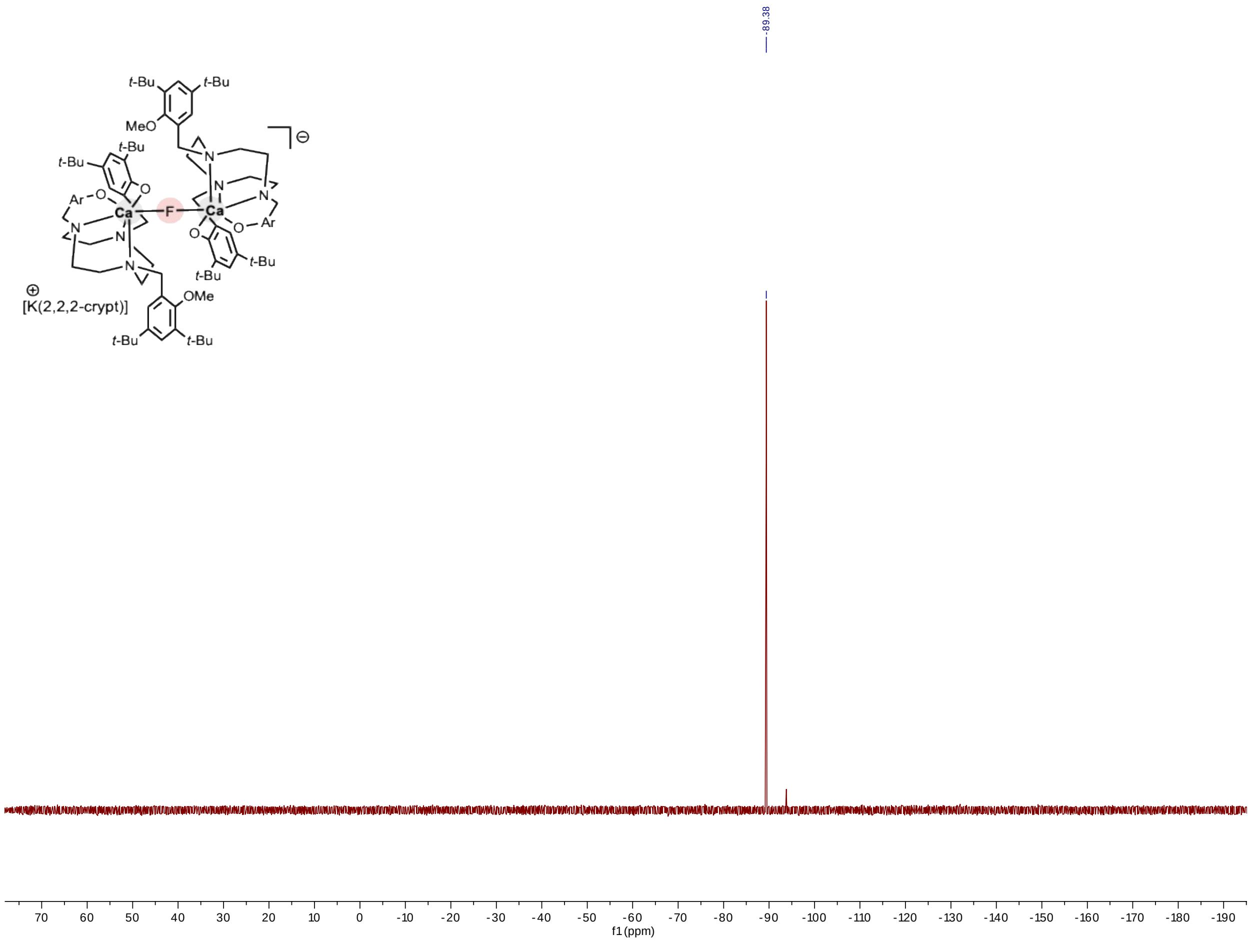

Figure S15. $^{19}F\{^{1}H\}$ NMR spectrum of compound **6** in THF-d_8.

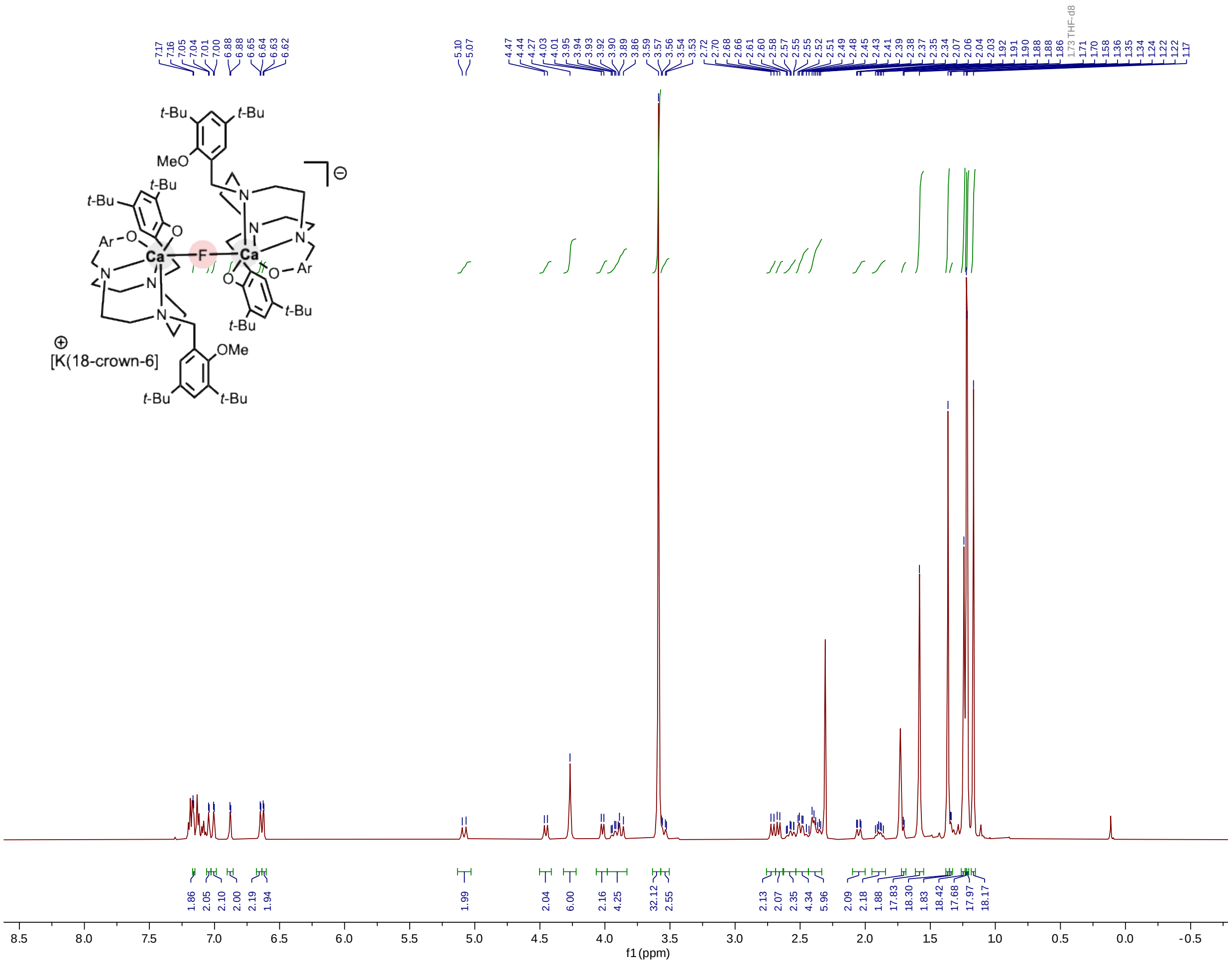

Figure S16. ^{1}H NMR spectrum of compound **7** in THF-d_8.

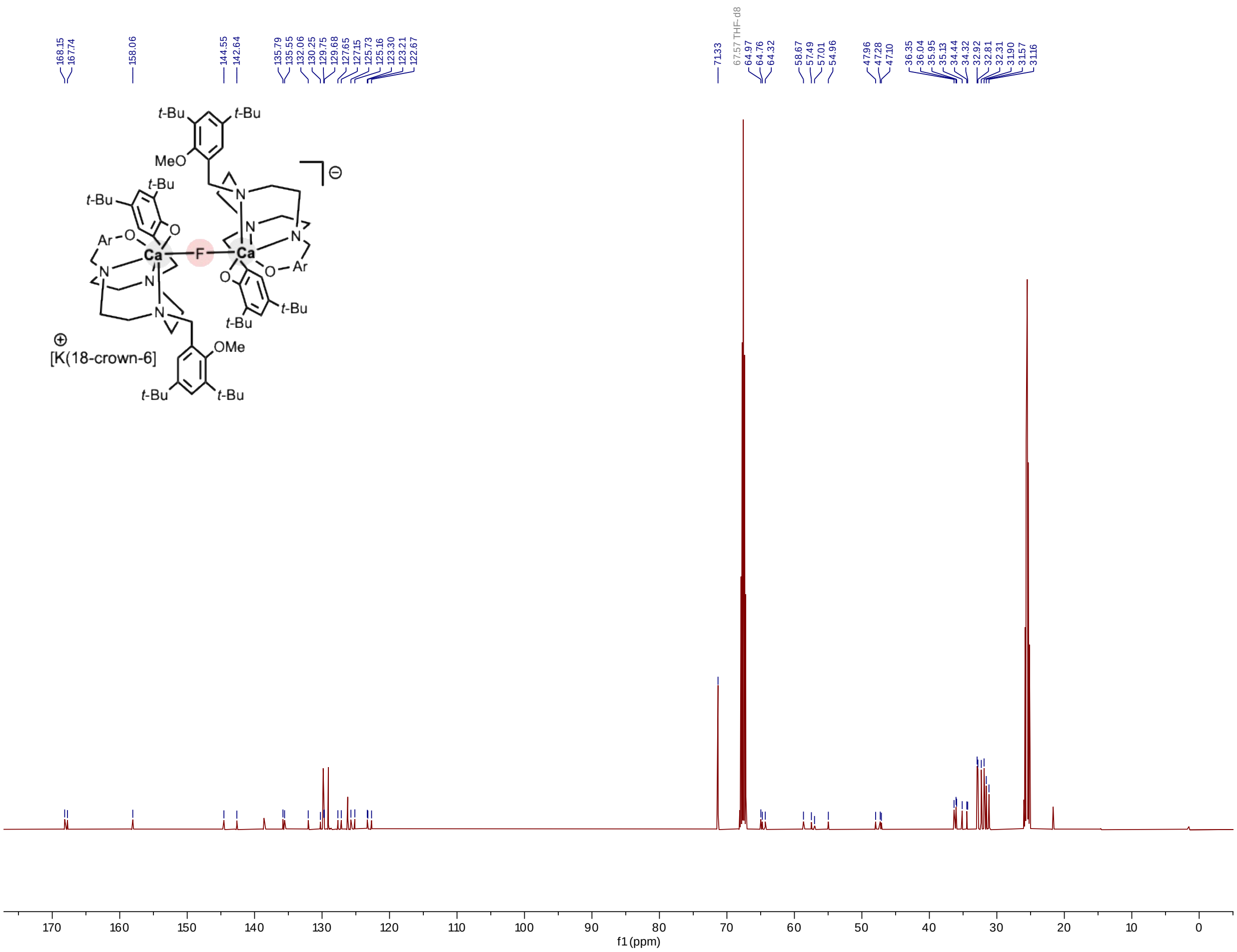

Figure S17. $^{13}C\{^{1}H\}$ NMR spectrum of compound **7** in THF-*d*8.

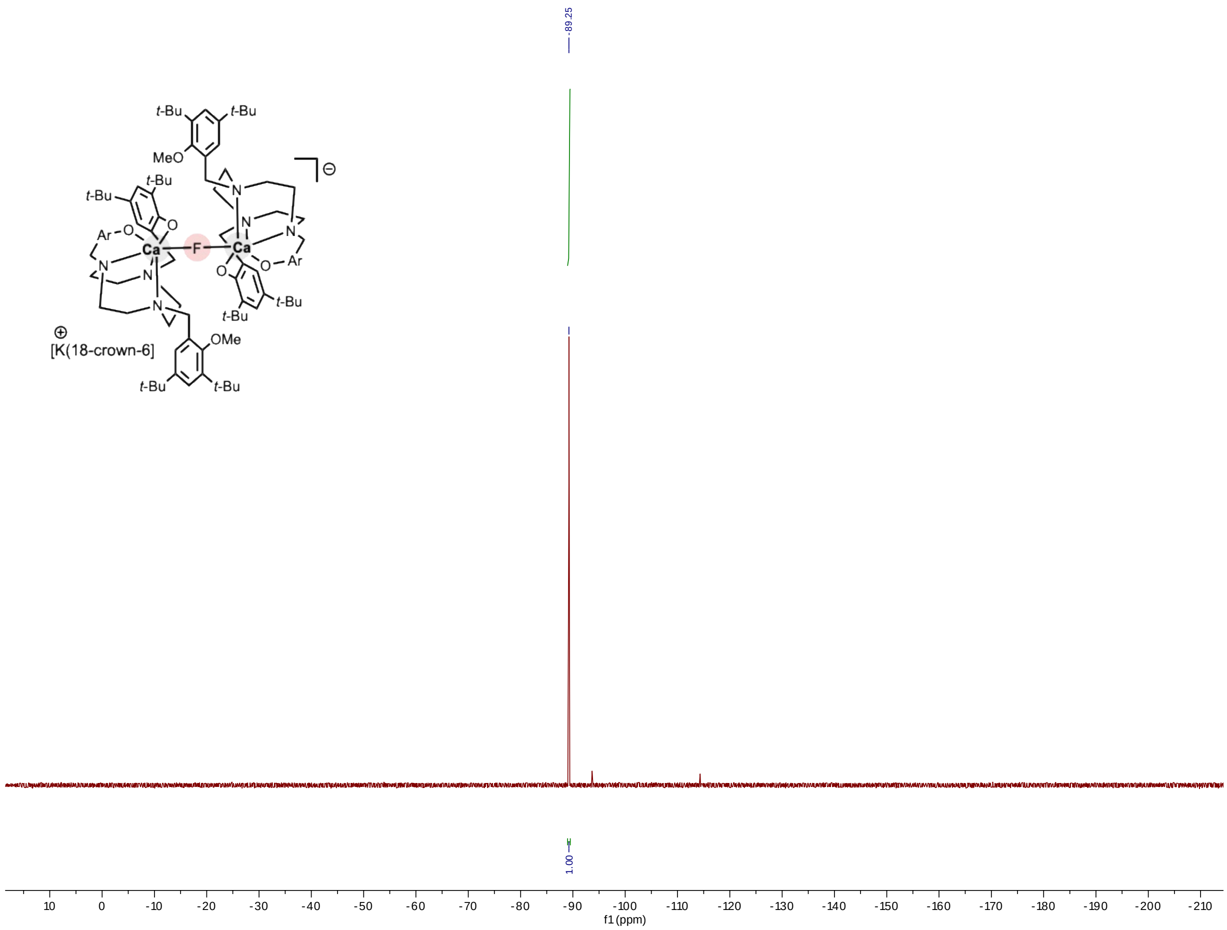

Figure S18. $^{19}F\{^{1}H\}$ NMR spectrum of compound **7** in THF-d_8.

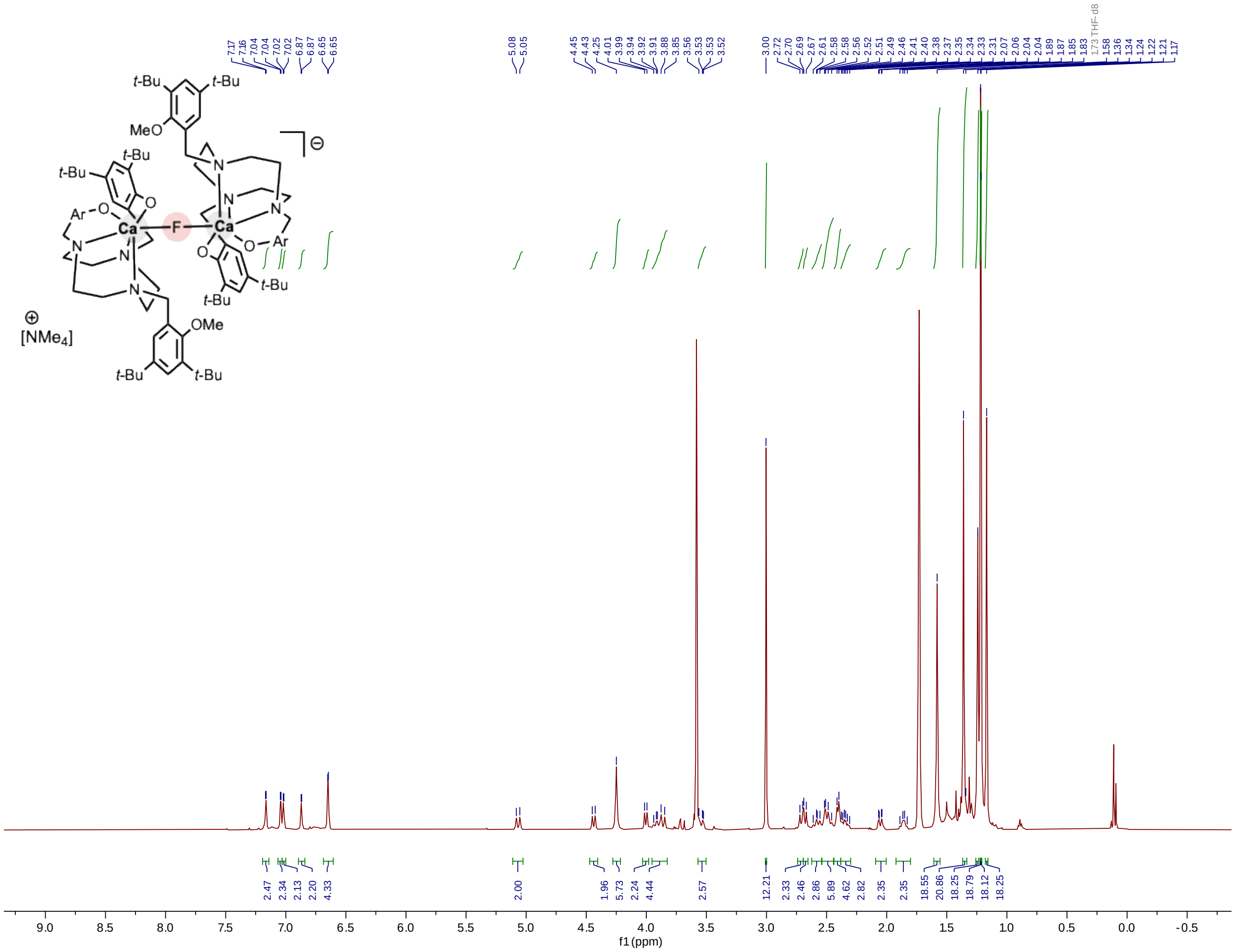

Figure S19. ${}^{1}H$ NMR spectrum of compound **8** in THF-d_8.

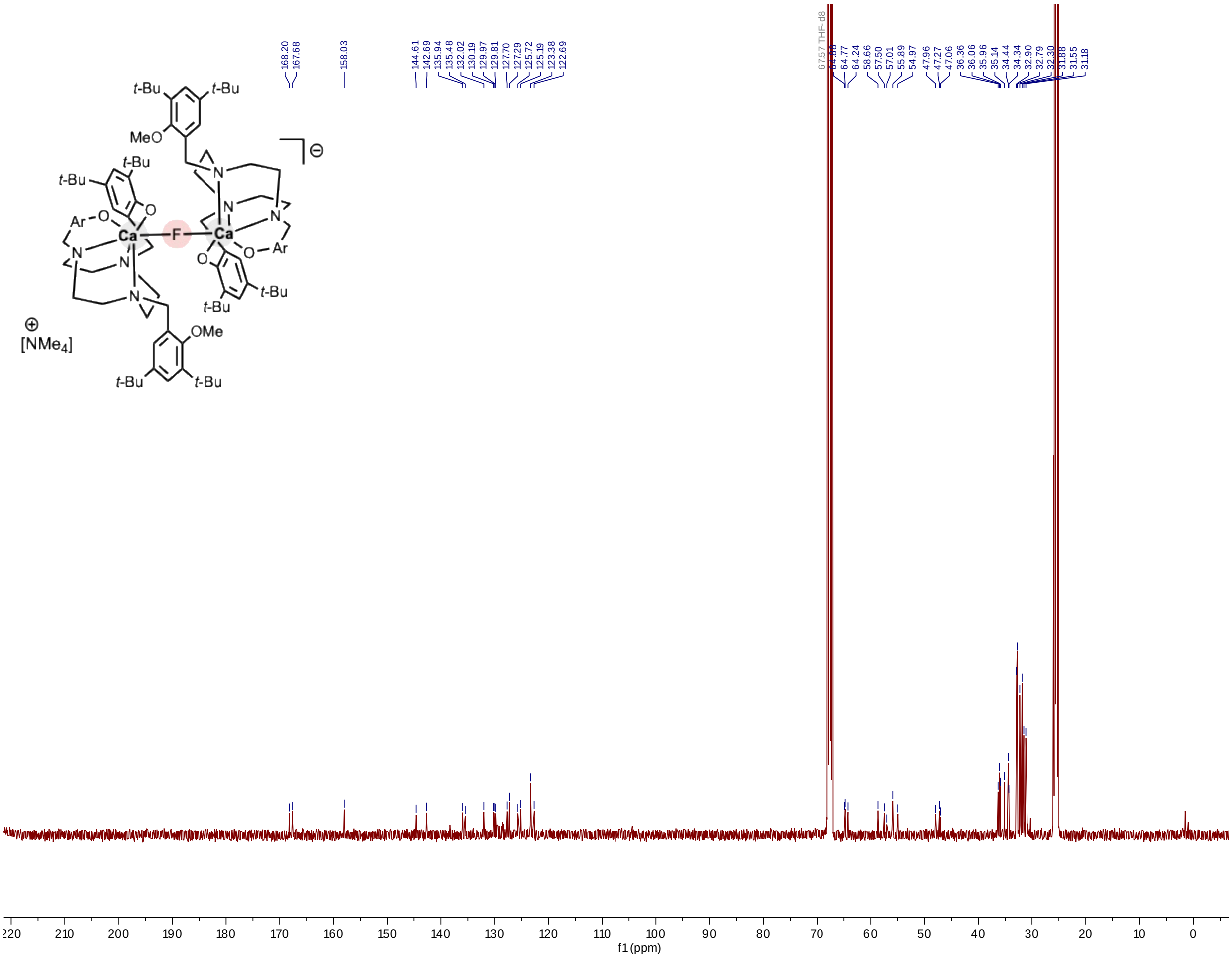

Figure S20. $^{13}C\{^{1}H\}$ NMR spectrum of compound **8** in THF-d_8.

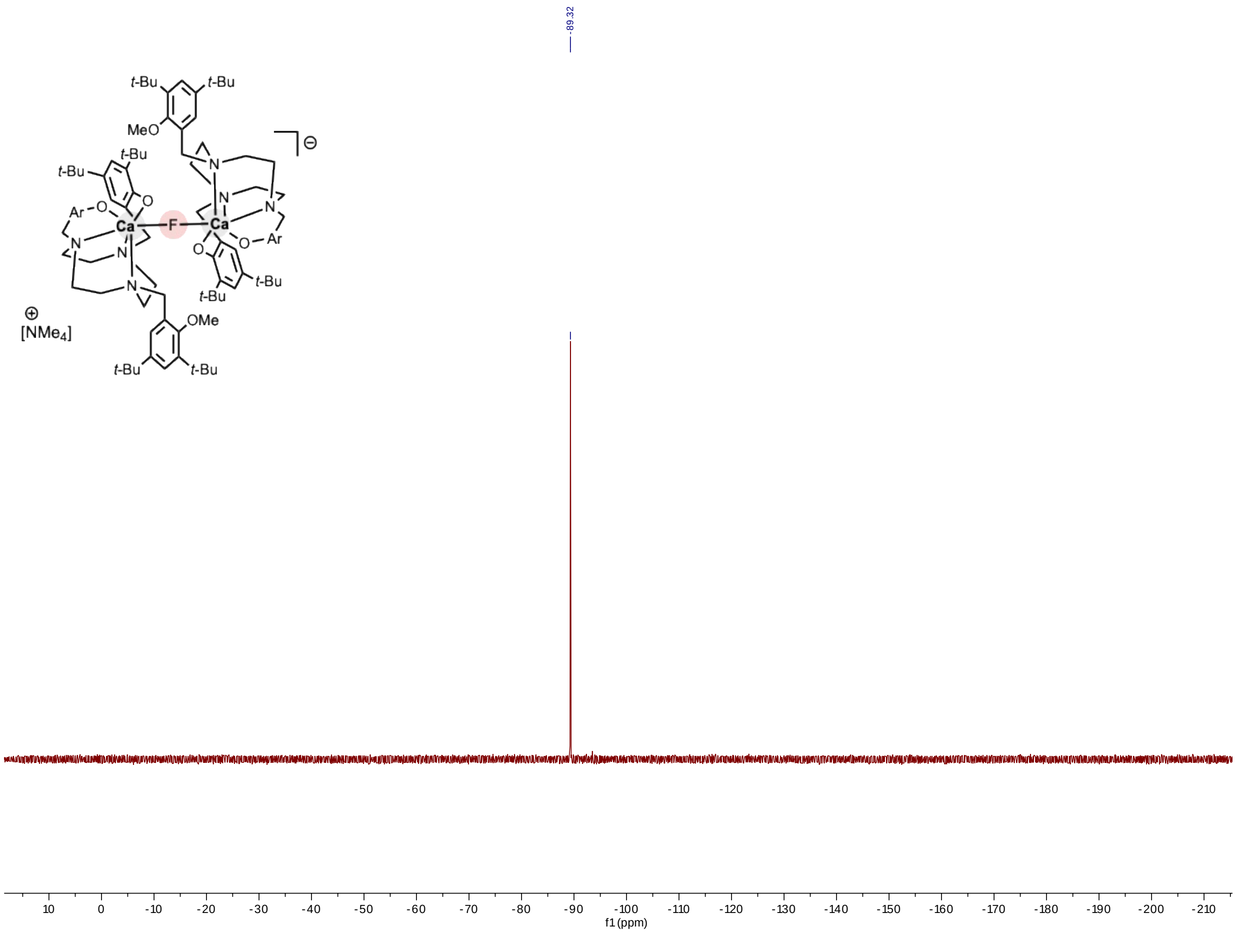

Figure S20. $^{19}F\{^{1}H\}$ NMR spectrum of compound **8** in THF-d_8

Preparation of $[NMe_4]_2[\{(t\text{-BuTACN})Ca\}(\mu\text{-}F)_2]$, 9

TMAF (22.3 mg, 239 µmol) was loaded into a flame dried Schlenk flask equipped with a Teflon-coated magnetic stir bar in a nitrogen filled glovebox. 1.5 mL of dry THF was added via cannula, and the mixture sonicated at 40 °C for 1 h then stirred for a further 24 h. In a separate flame dried Schlenk flask **3** (100 mg, 120 µmol) was dissolved in 3.5 mL of THF and added dropwise to the TMAF solution at room temperature. After addition, the reaction mixture was stirred for an additional 30 min and a new fluoride-containing species was observed as the major product by ^1H and ^{19}F NMR measurements. The stirring of the reaction mixture was stopped and, when the precipitate had settled, was filtered by cannula into another Schlenk flask. Volatiles were removed *in vacuo*, and the resulting solid washed with hexane (0.3 mL x 3), to afford, after drying, complex **9** as a white solid (58 mg, 52%).

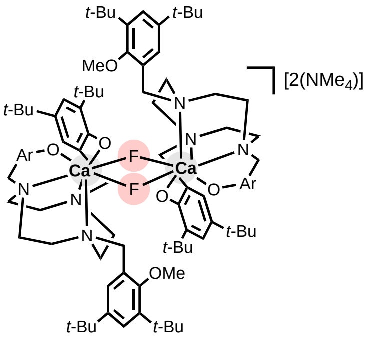

^1H NMR (400 MHz, C_6D_6) δ 7.62 (t, J = 2.3 Hz, 4H, *Ph*), 7.42 (d, J = 2.5 Hz, 2H, *Ph*), 7.15 (s, 4H, *Ph* + overlap with C_6D_6 7.16 ppm peak), 7.06 (d, J = 2.7 Hz, 2H, *Ph*), 5.20 (d, J = 14.9 Hz, 2H, NCH_2Ph), 4.61 (d, J = 10.7 Hz, 2H, NCH_2Ph), 4.52 (d, J = 10.7 Hz, 2H, NCH_2Ph), 4.02 (d, J = 14.9 Hz, 2H, NCH_2Ph), 3.70–3.64 (m, 2H, NCH_2CH_2N), 3.61 (s, 6H, $PhOCH_3$), 3.26 (d, J = 10.8 Hz, 2H, NCH_2Ph), 3.16 (dd, J = 14.3, 3.1 Hz, 2H, NCH_2CH_2N), 2.99 (d, J = 10.7 Hz, 2H, NCH_2Ph), 2.65 (d, J = 11.7 Hz, 4H, NCH_2CH_2N), 2.54 (s, 24H, $N(CH_3)_4^+$), 2.51–2.47 (m, 2H, NCH_2CH_2N), 2.30–2.12 (m, 8H, NCH_2CH_2N), 2.07 (m, 2H, NCH_2CH_2N), 1.94 (s, 18H, $PhC(CH_3)_3$), 1.89 (s, 18H, $PhC(CH_3)_3$), 1.79 (s, 2H, NCH_2CH_2N), 1.68–1.63 (m, 2H, NCH_2CH_2N), 1.54 (s, 18H, $PhC(CH_3)_3$), 1.50 (s, 18H, $PhC(CH_3)_3$), 1.47 (s, 18H, $PhC(CH_3)_3$), 1.29 (s, 18H, $PhC(CH_3)_3$). **$^{13}C\{^1H\}$ NMR** (100 MHz, C_6D_6) δ 167.4, 167.3, 157.4, 145.3, 142.4, 136.1, 134.5, 130.9, 130.8, 129.9, 129.3, 129.1, 127.5, 125.1, 124.5, 123.8, 123.5, 123.2 (*Ph*), 64.2, 63.4 (NCH_2Ph), 62.7 ($PhOCH_3$), 57.6, 56.2 (NCH_2CH_2N), 55.2 ($N(CH_3)_4^+$ + NCH_2CH_2N + NCH_2Ph), 47.8, 45.9 (NCH_2CH_2N), 36.0, 35.9, 35.4, 34.5, 34.1 ($PhC(CH_3)_3$), 32.7, 31.7, 31.5, 31.0 ($PhC(CH_3)_3$). **$^{19}F\{^1H\}$ NMR** (377 MHz, C_6D_6) δ -82.1.

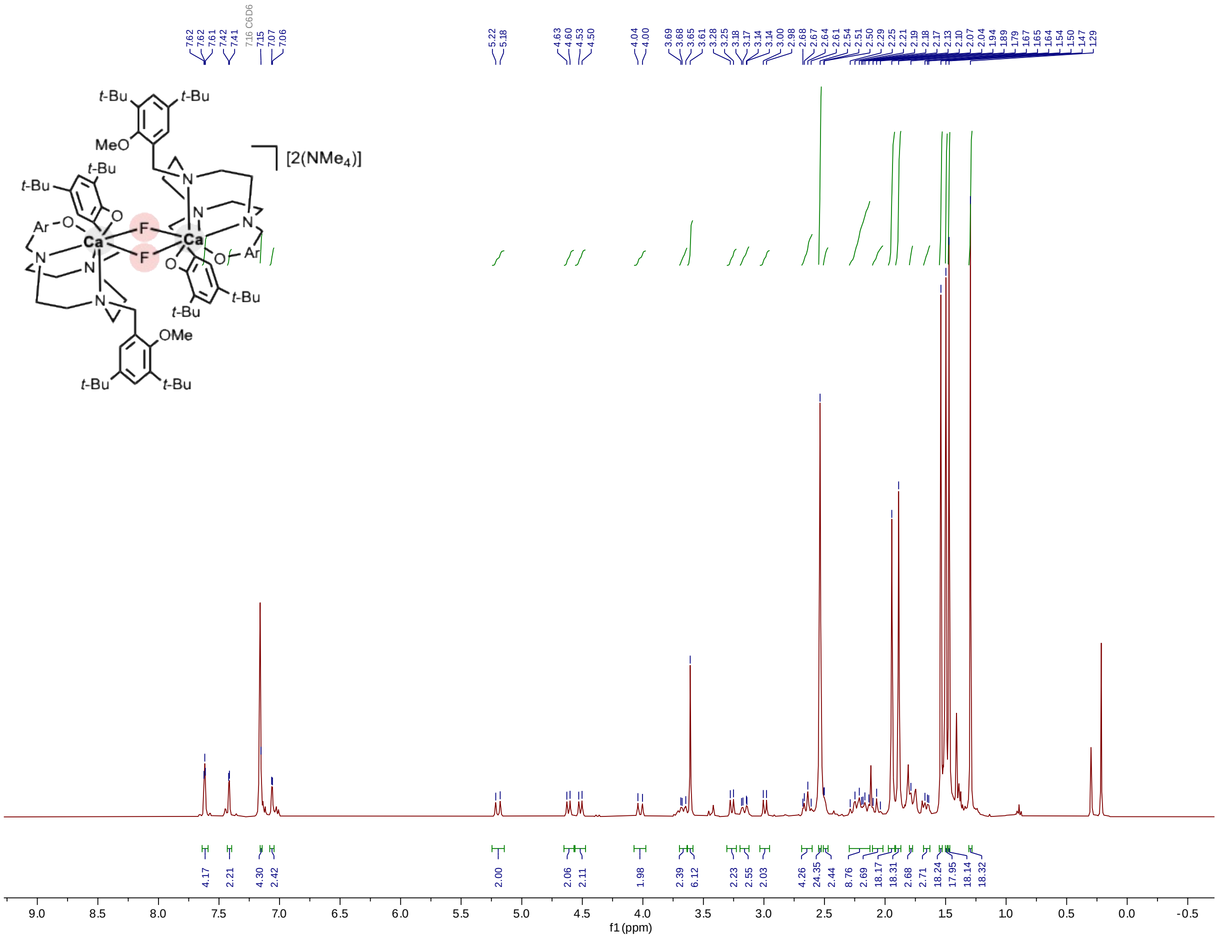

Figure S21. ^{1}H NMR spectrum of compound **9** in C_6D_6.

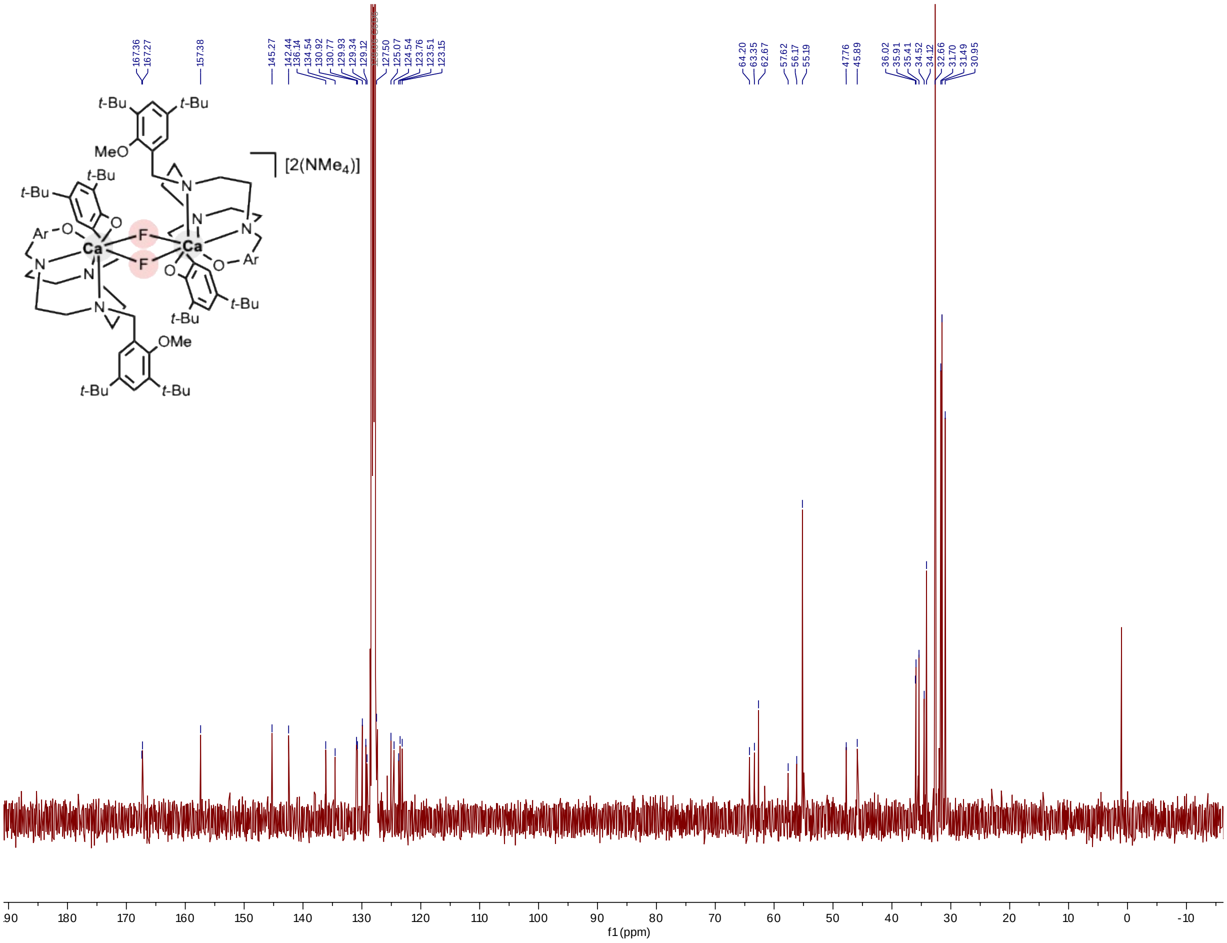

Figure S22. $^{13}C\{^1H\}$ NMR spectrum of compound **9** in C_6D_6.

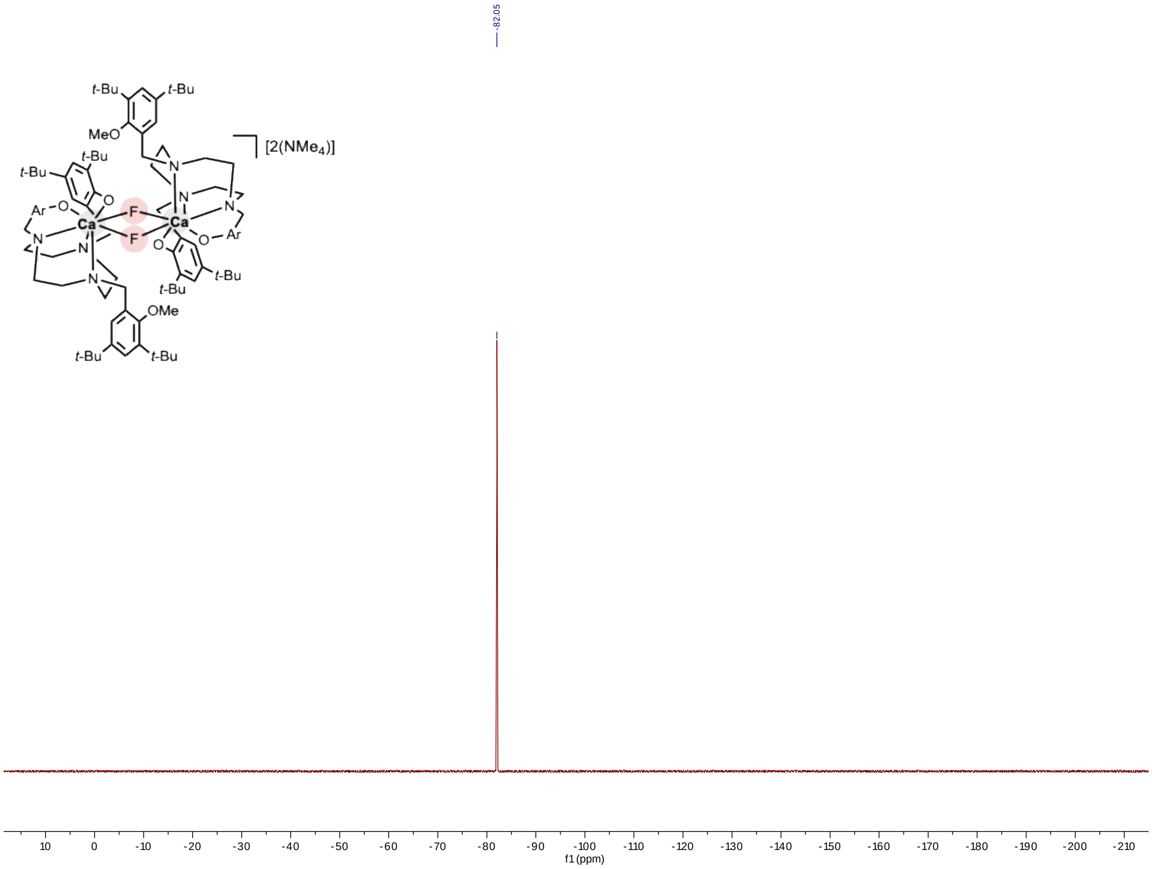

Figure S23. $^{19}F\{^{1}H\}$ NMR spectrum of compound **9** in C_6D_6.

^{19}F DOSY NMR Spectra of Compounds 8 and 9

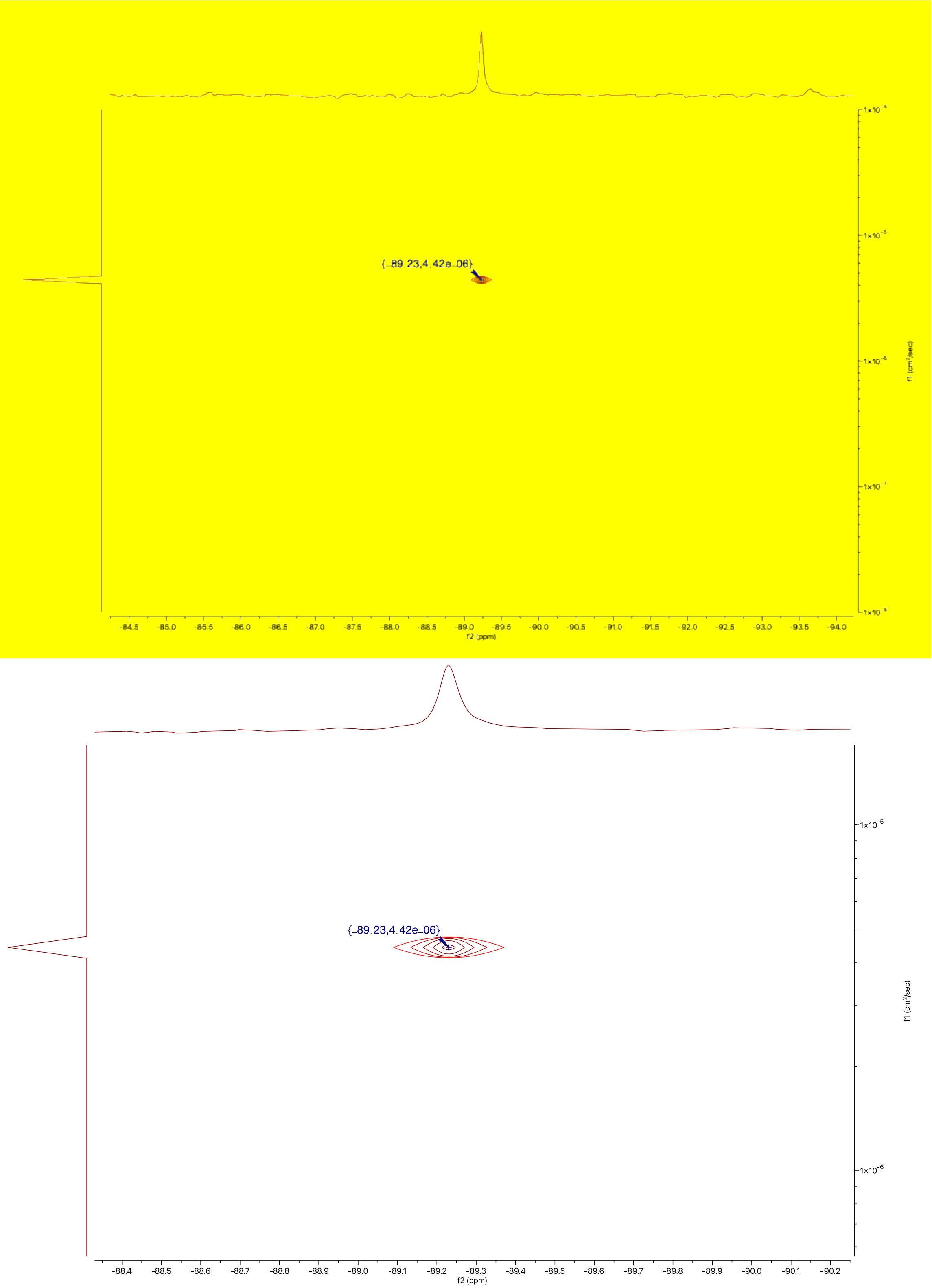

Figure S24. ^{19}F DOSY NMR spectrum (full and zoomed in) of compound **8** in THF-d_8.

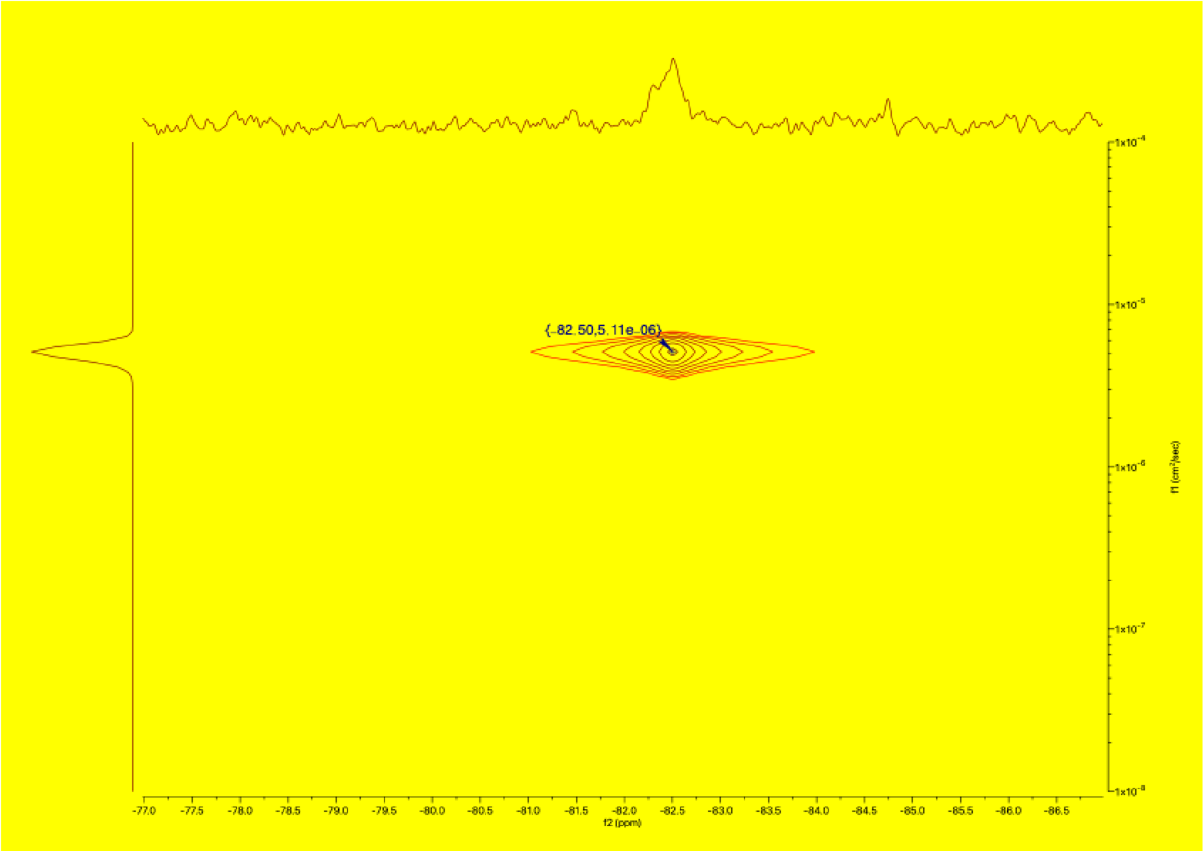

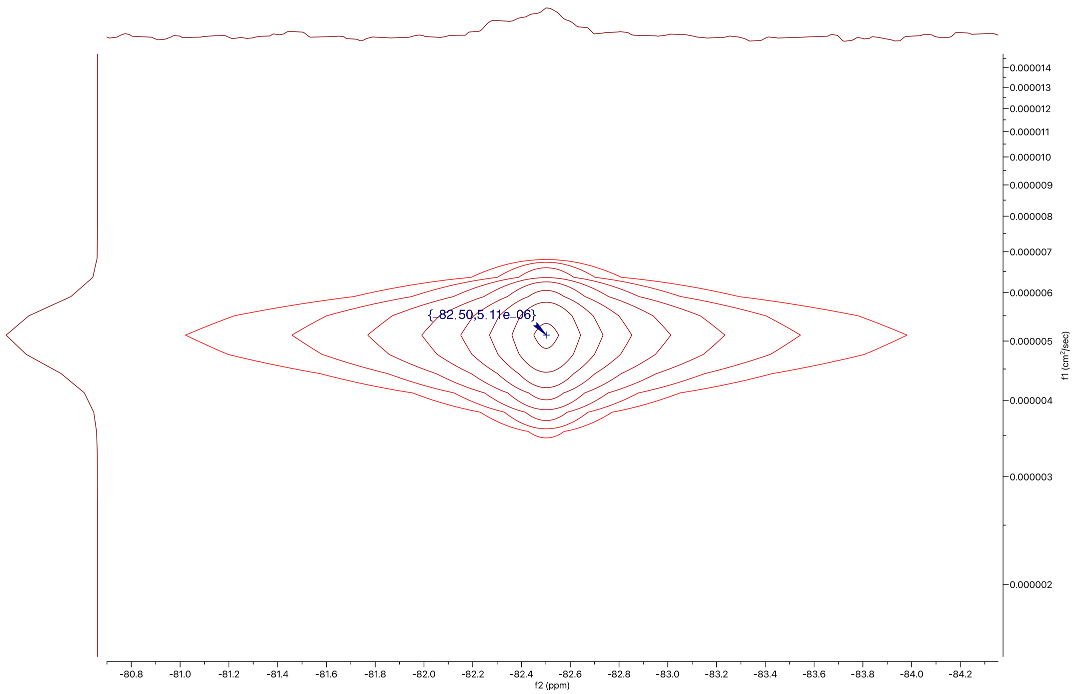

Figure S25. ^{19}F DOSY NMR spectrum (full and zoomed in) of compound **9** in THF-d_8.

ECC-MW-Calculation

Using Stalke's ECC-MW-Determination Calculator[4] we were able to calculate the estimated molecular weights of compounds 8 and 9. The calculated MW of compound **8** was determined to be 1858 g/mol (ΔMW = 7%) whereas the theoretical MW is 1995 g/mol. In addition, the calculated MW of compound **9** was determined to be 1449 g/mol (ΔMW = 22%) whereas the theoretical MW is 1859 g/mol. Even though the accuracy is not optimal in our case, these calculations do not contradict our proposed dimeric structure for compound 9 based on our hydrodynamic radius calculations.

Reactivity of [K(2.2.2-cryptand)][{(*t*-BuTACN-Me)Ca}$_2$F] 6 with electrophiles

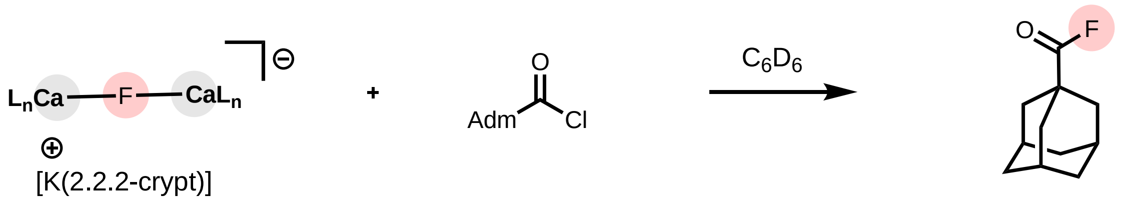

6

10, 58%
(23.2 ppm)

To a J-Young NMR tube were added **6** (10 mg, 4.9 µmol), 1-adamantanecarbonyl chloride (0.95 mg, 4.9 µmol), and C_6D_6 (0.5 mL) in a nitrogen filled glovebox. The NMR tube was inverted, and the reaction was monitored over several days by ^{19}F NMR. After 48 h, a fluorobenzene internal standard (0.9 µL) was added and the NMR yield of compound **10** was determined to be 58% yield via ^{19}F NMR.

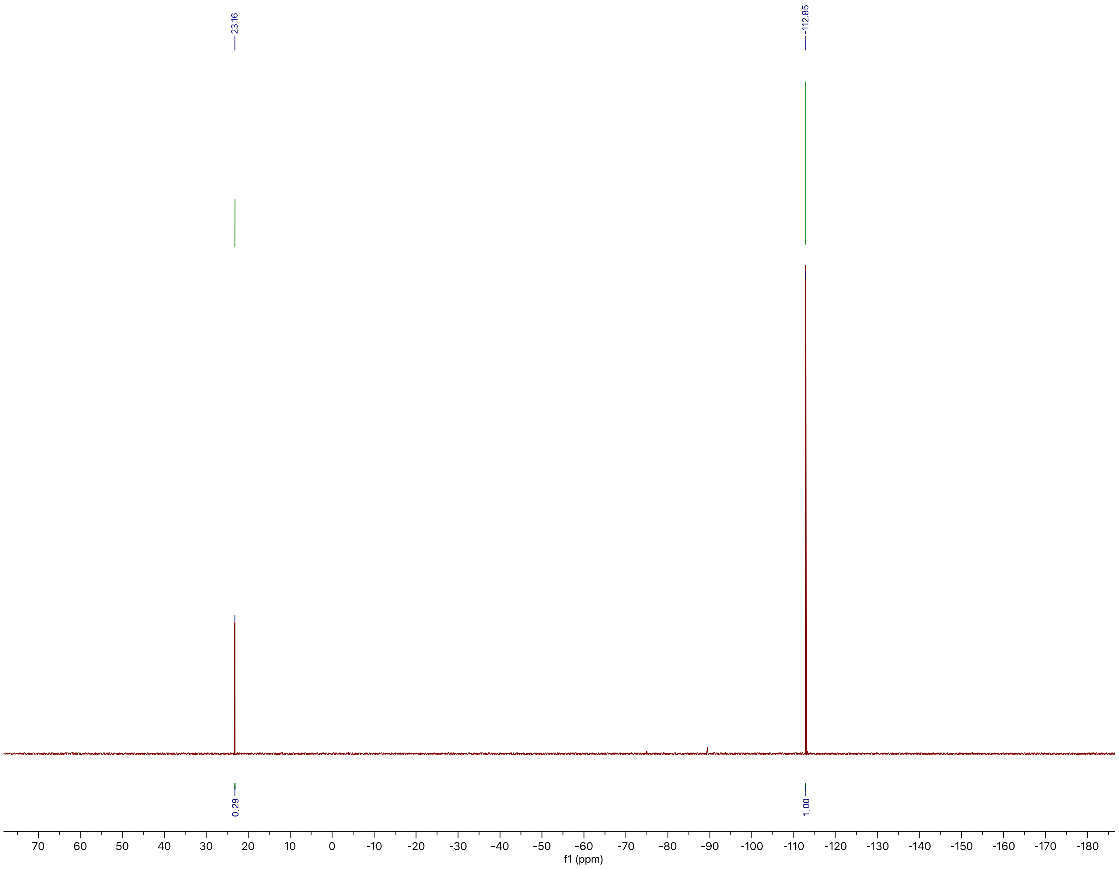

Figure S26. Crude $^{19}F\{^1H\}$ NMR spectrum of compound **10** in C_6D_6 after 48 h.

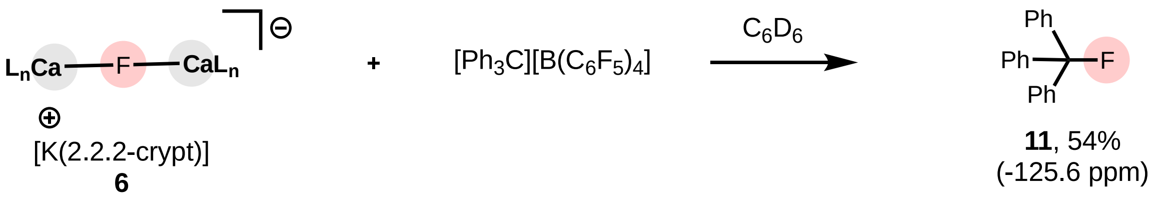

To a J-Young NMR tube were added **6** (10 mg, 4.9 µmol), triphenylcarbenium tetrakis(pentafluorophenyl)borate (4.4 mg, 4.8 µmol), and C_6D_6 (0.5 mL) in a nitrogen filled glovebox. The NMR tube was inverted, and the reaction was monitored over 24 h by ^{19}F NMR. After 24 h, a fluorobenzene internal standard (0.9 µL) was added and the NMR yield of compound **11** was determined to be 54% yield via ^{19}F NMR.

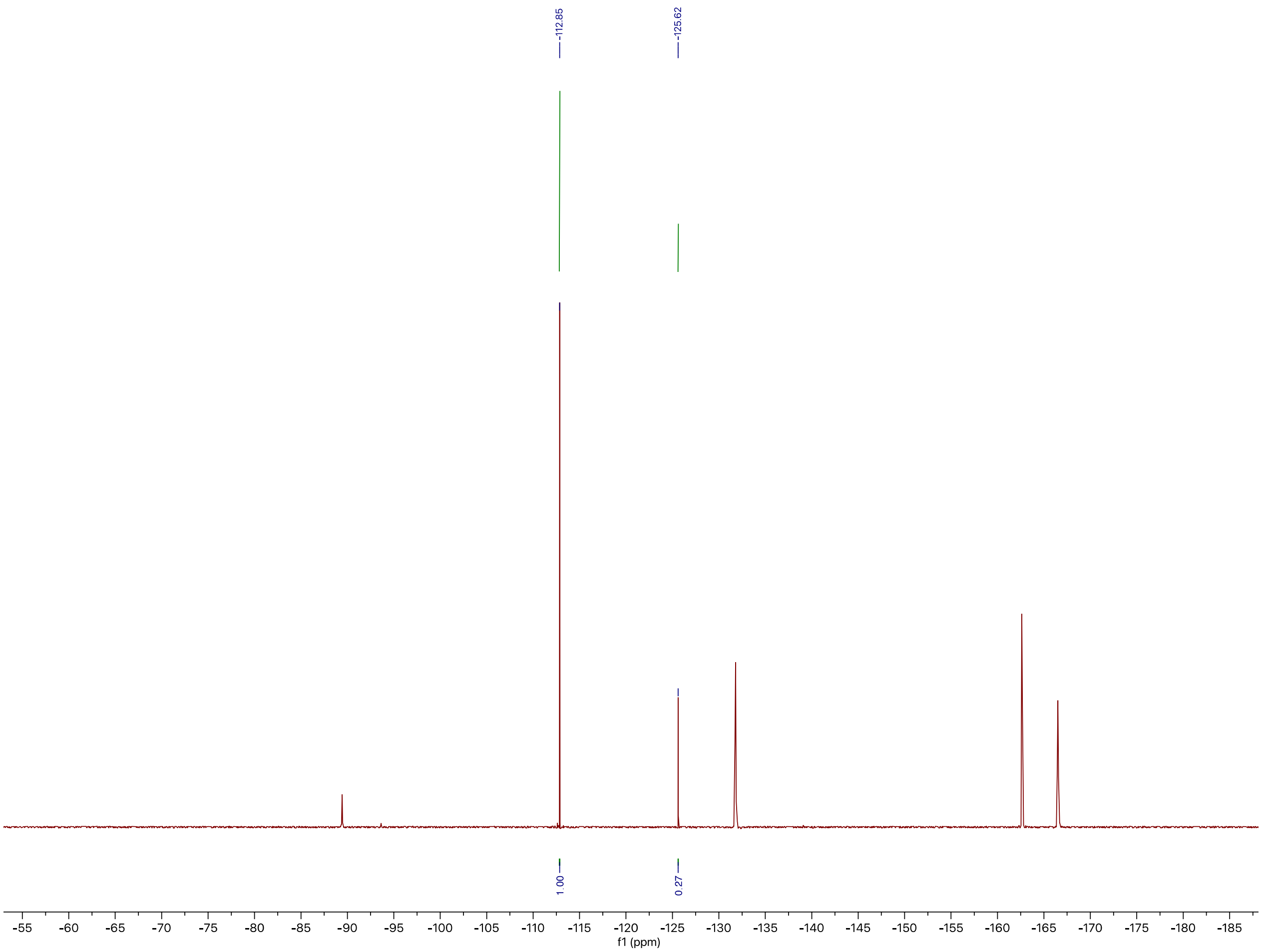

Figure S27. Crude $^{19}F\{^1H\}$ NMR spectrum of compound **11** in C_6D_6 after 24 h.

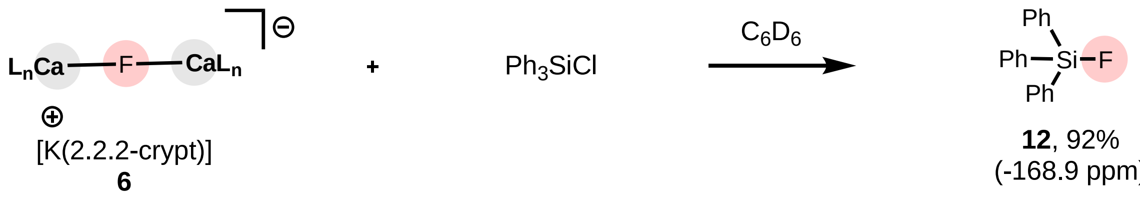

To a J-Young NMR tube were added **6** (5.0 mg, 2.4 µmol), chlorotriphenylsilane (0.70 mg, 2.4 µmol), and C_6D_6 (0.5 mL) in a nitrogen filled glovebox. The NMR tube was inverted, and the reaction was monitored over 72 h by ^{19}F NMR. After 72 h, a fluorobenzene internal standard (0.9 µL) was added and the NMR yield of compound **11** was determined to be 92% yield via ^{19}F NMR.

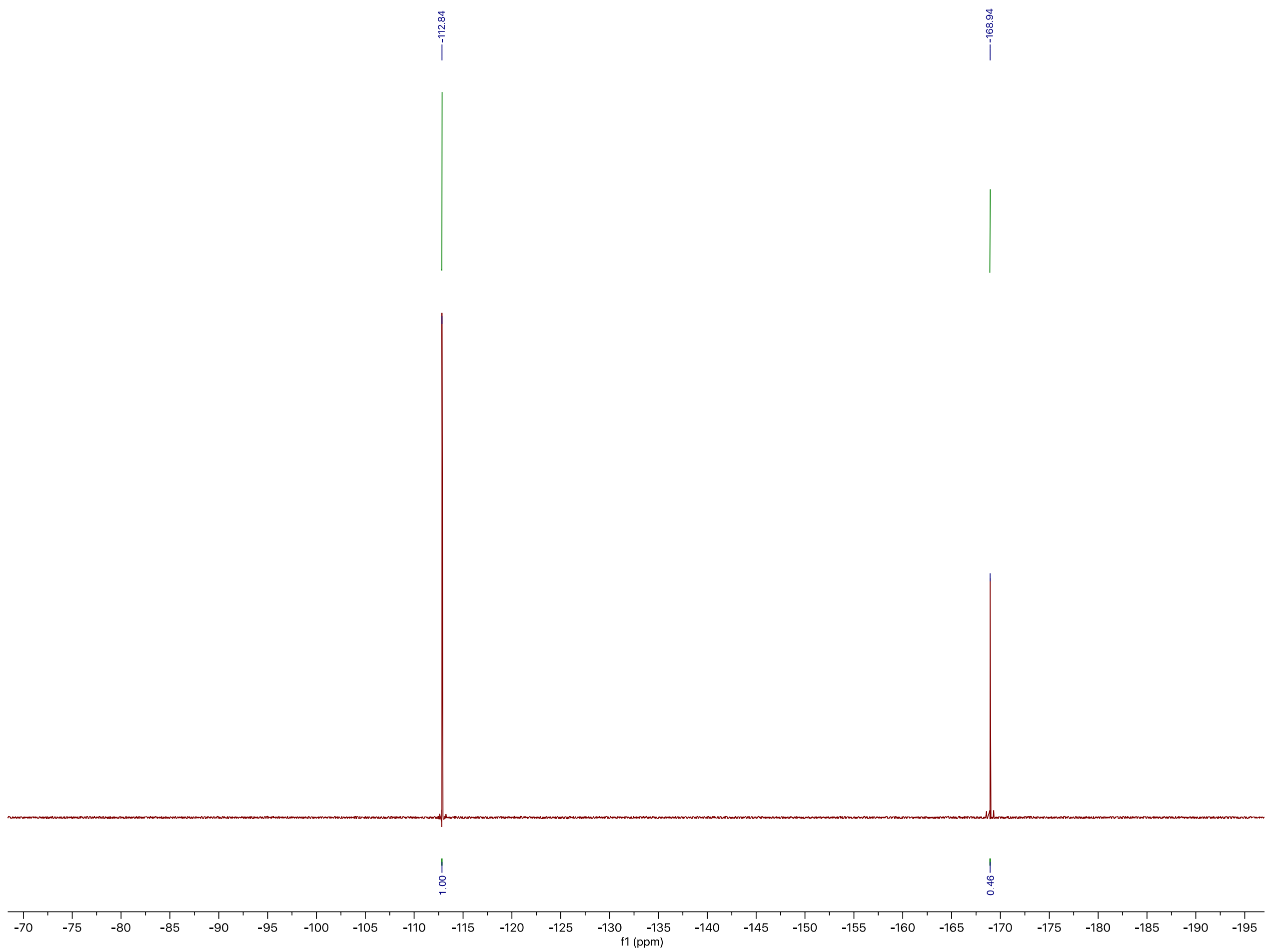

Figure S28. Crude $^{19}F\{^1H\}$ NMR spectrum of compound **12** in C_6D_6 after 72 h.

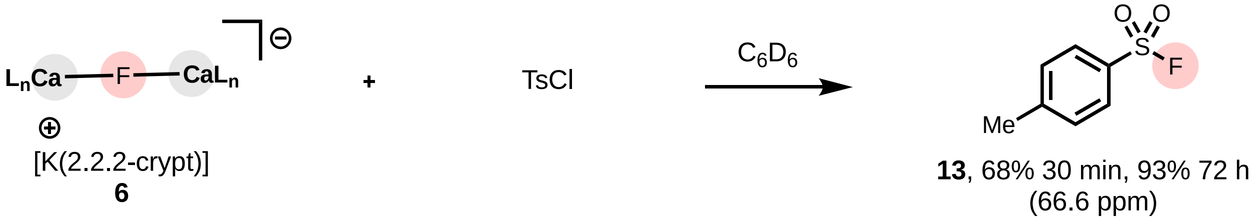

To a J-Young NMR tube were added **6** (15 mg, 7.2 µmol), tosyl chloride (1.4 mg, 7.2 µmol µmol), a fluorobenzene internal standard (0.9 µL), and C_6D_6 (0.5 mL) in a nitrogen filled glovebox. The NMR tube was inverted, and the reaction was monitored over 72 h by ^{19}F NMR. After 30 min, the NMR yield of compound **13** was determined to be 68% yield via ^{19}F NMR. After 72 h, the NMR yield of compound **13** was determined to be 93% yield via ^{19}F NMR.

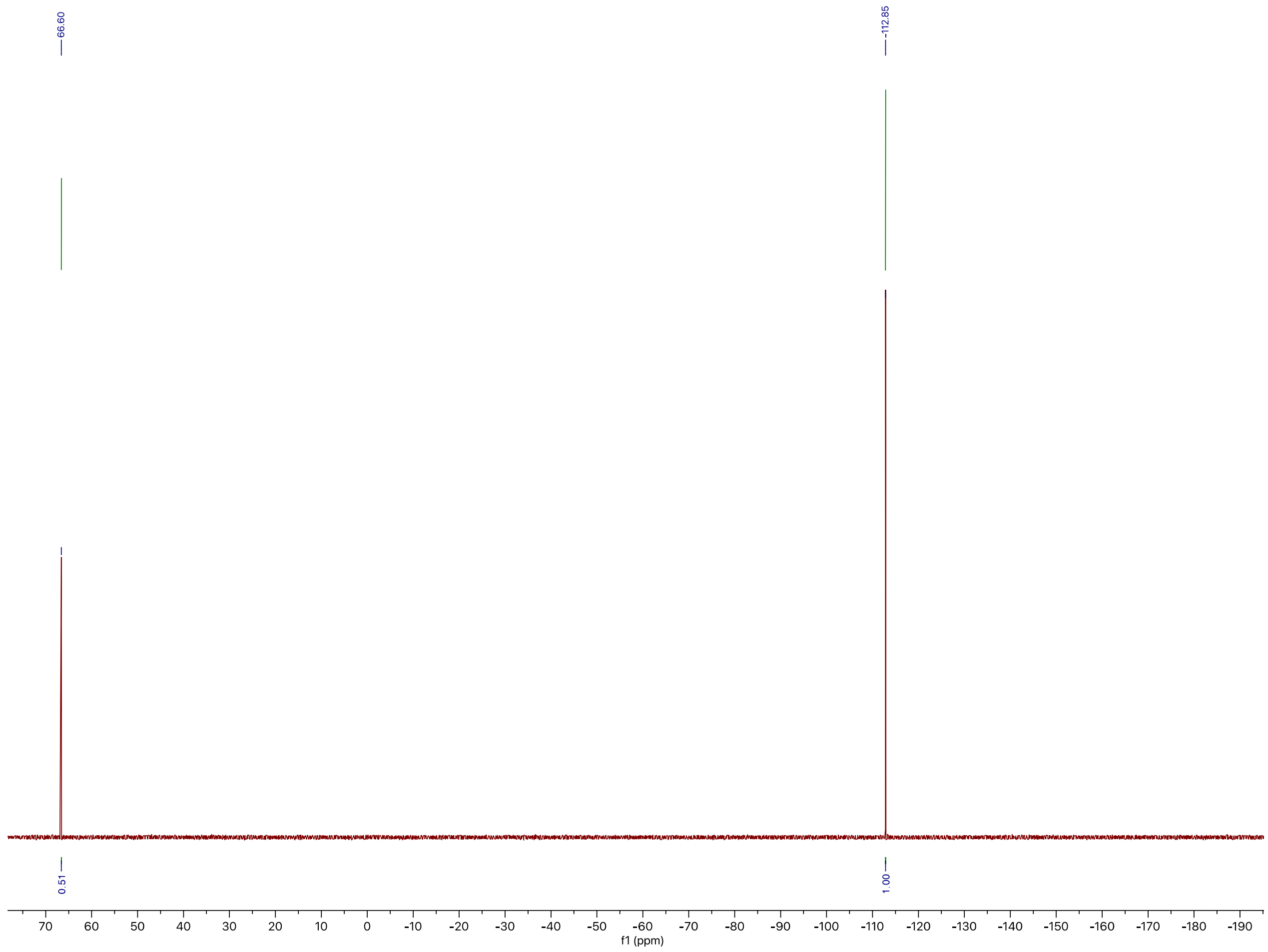

Figure S29. Crude $^{19}F\{^1H\}$ NMR spectrum of compound **13** in C_6D_6 after 30 min.

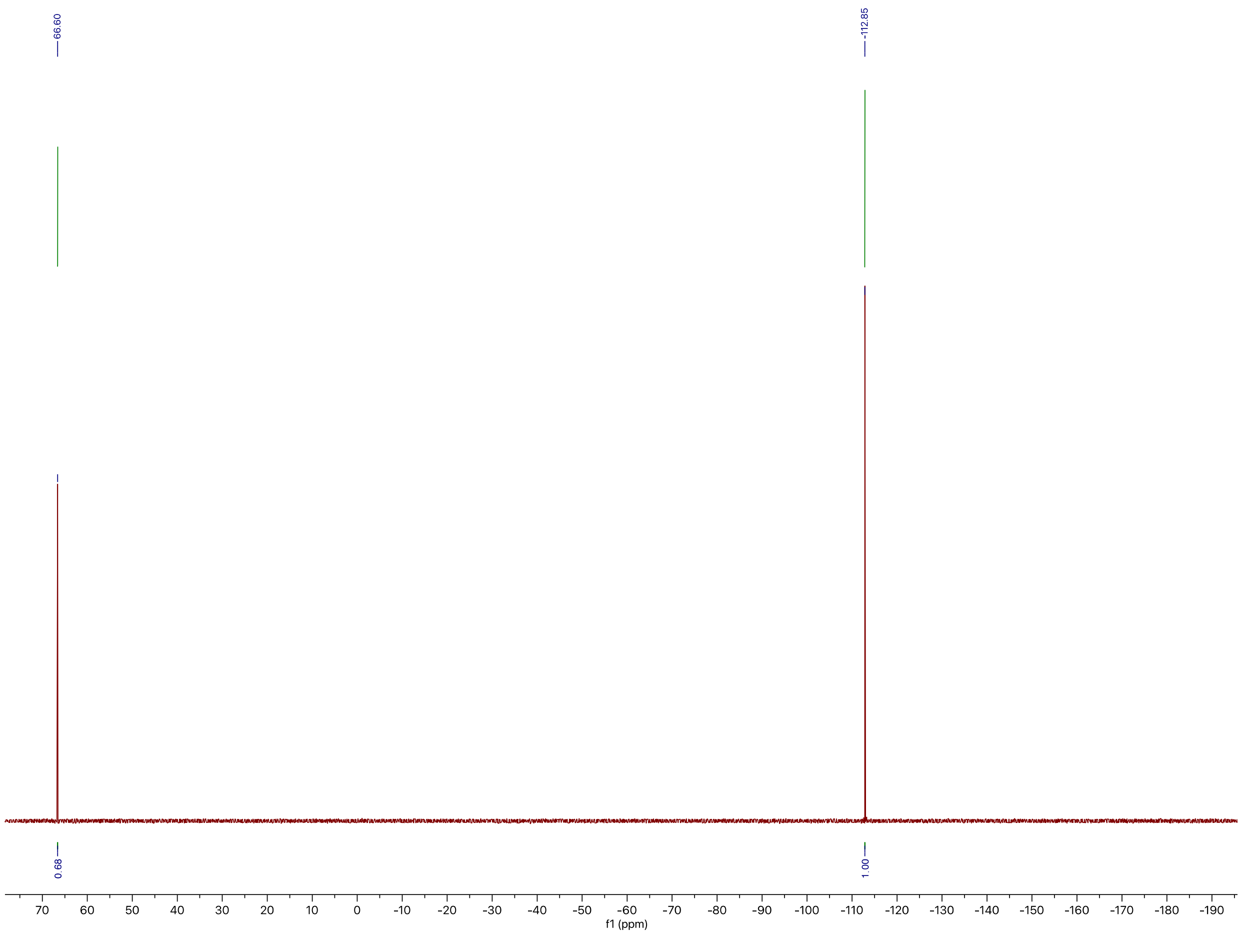

Figure S30. Crude $^{19}F\{^1H\}$ NMR spectrum of compound **13** in C_6D_6 after 72 h.

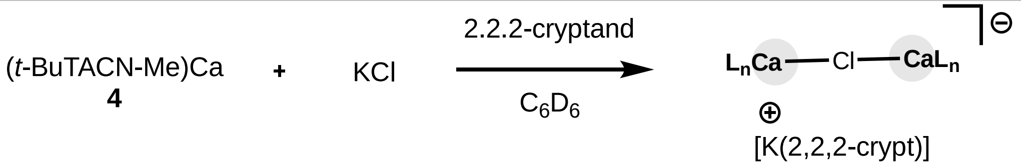

To a J-Young NMR tube were added **4** (20 mg, 24 μmol), potassium chloride (8.9 mg, 120 μmol), 2.2.2-cryptand (9.0 mg, 24 μmol), and C_6D_6 (0.5 mL) in a nitrogen filled glovebox. The NMR tube was placed in an 80 °C oil bath and after two weeks, near complete formation of the [K(2.2.2-crypt)][{(*t*-BuTACN-Me)Ca}$_2$(μ-Cl)] was observed. The stacked ^{1}H NMR spectra of three of the reactivity entries match the spectrum of the independently synthesized complex as determined by the diagnostic benzylic and methoxy proton resonances (from the TACN backbone) shown below.

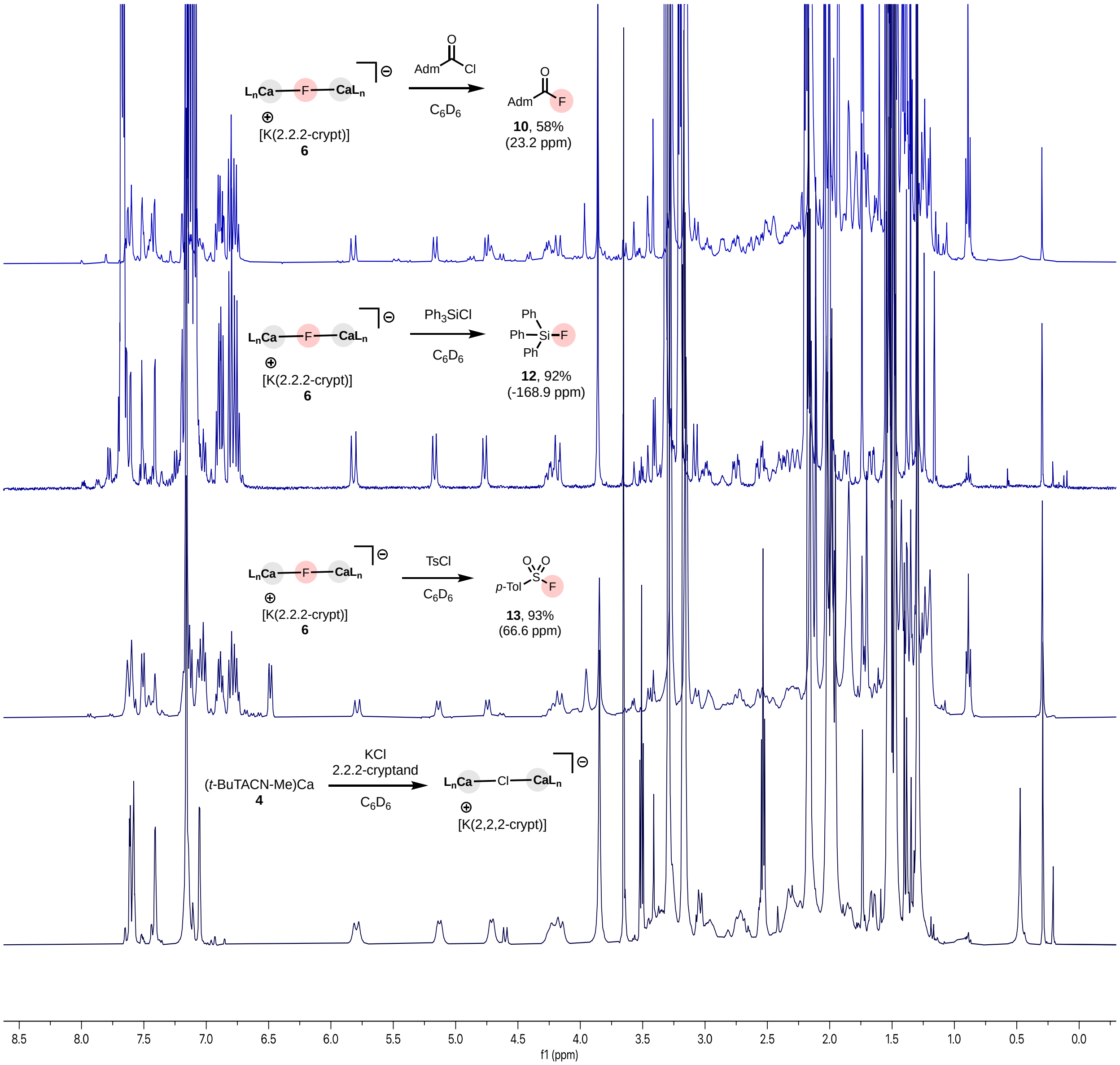

Figure S31. Stacked crude ^{1}H NMR spectra of reactions with compound **6** and independent synthesis of [K(2.2.2-crypt)][{(*t*-BuTACN-Me)Ca}$_2$(μ-Cl)].

Fluorination Outcomes of Complexes 2 and 3

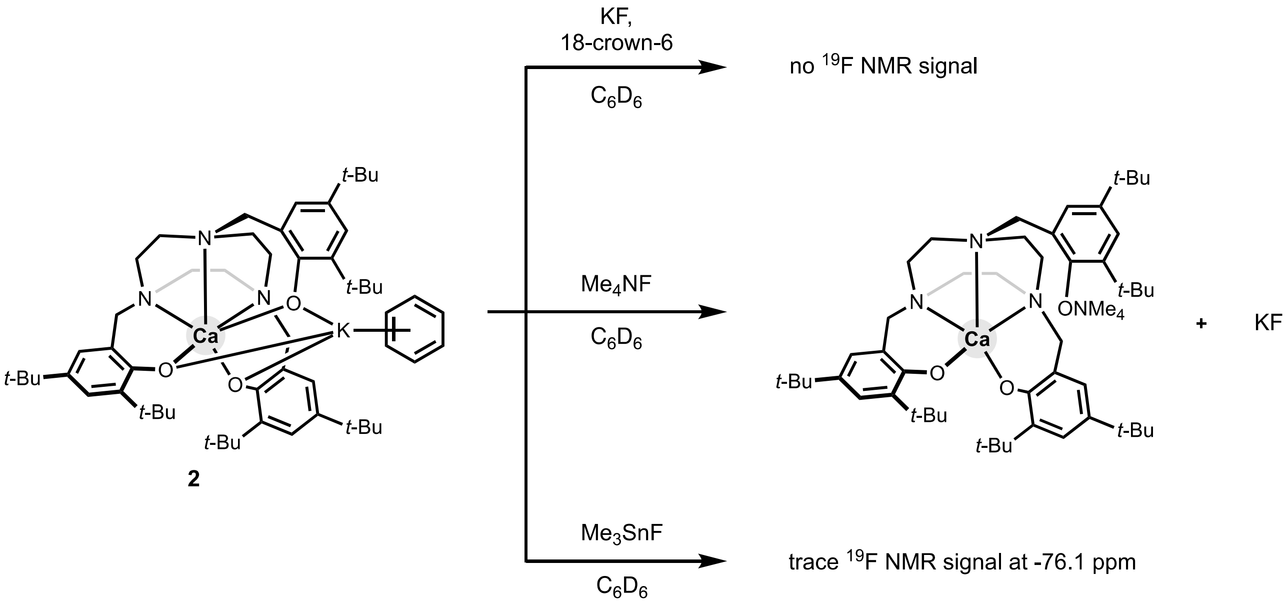

Figure S32. Unsuccessful fluorination attempts of compound **2**.

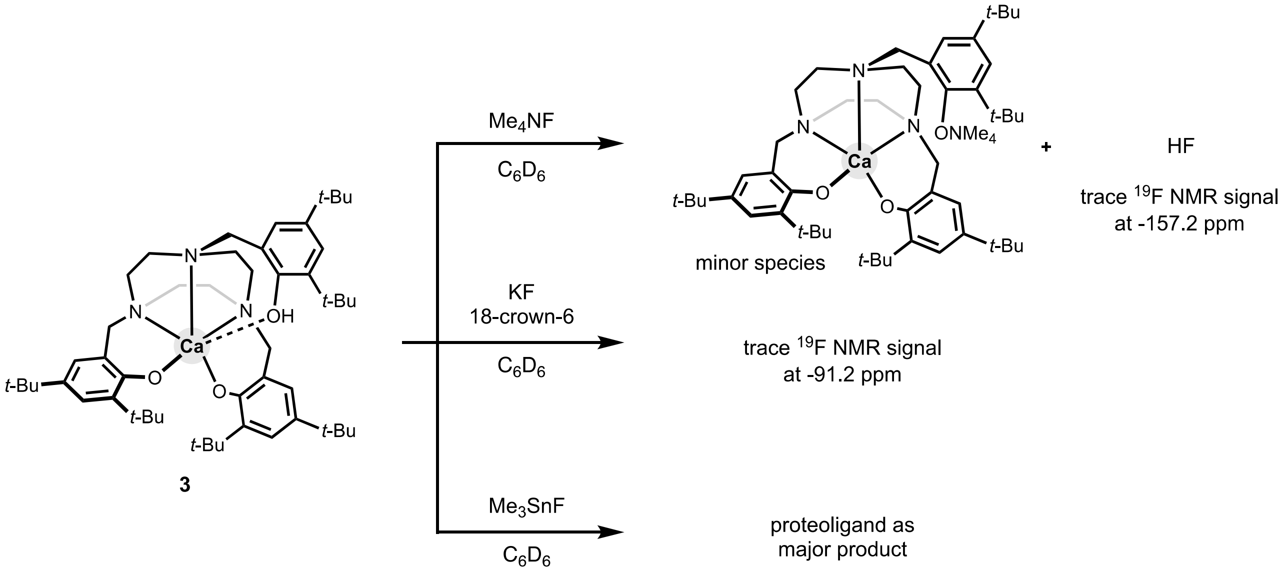

Figure S33. Unsuccessful fluorination attempts of compound **3**.

Unsuccessful Functionalizations of Complex 2

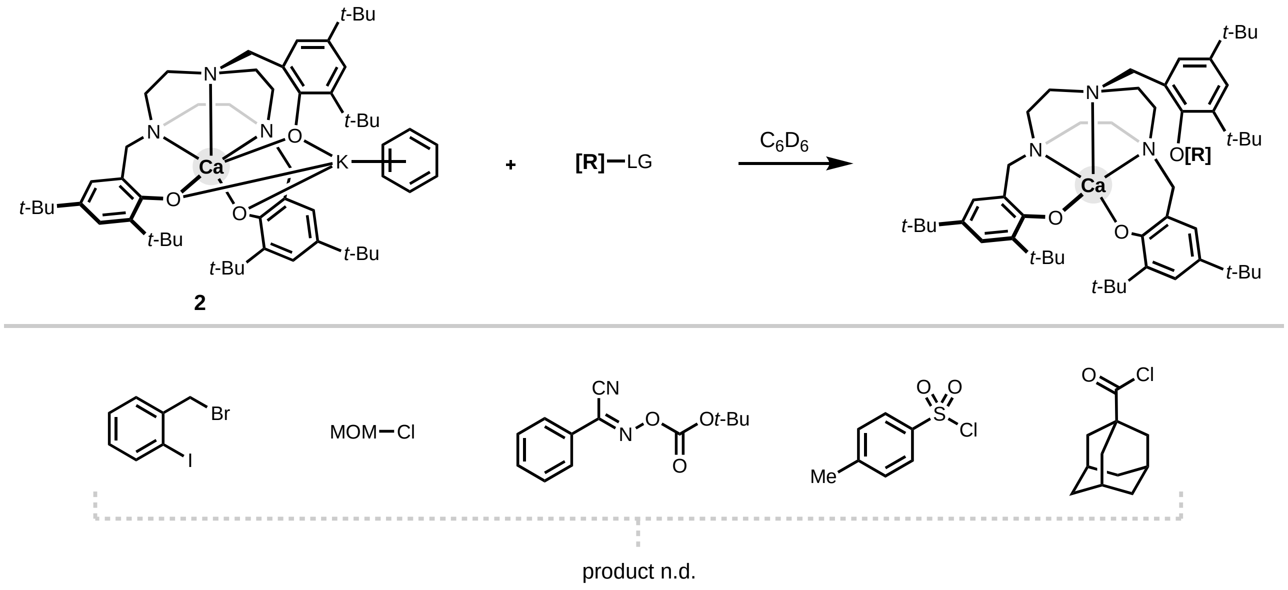

Figure S34. Unsuccessful functionalization attempts of compound **2**.

Single Crystal X-ray Structure Determination

All crystallographic data were collected on an Oxford Diffraction/Agilent SuperNova diffractometer equipped with a 135 mm Atlas CCD area detector or a Rigaku XtaLAB Synergy-DW VHF instrument equipped with a PhotonJet-R dual wavelength rotating anode and HyPix-Arc 150° detector. Crystals were prepared with Paratone-N or perfluorinated oil, mounted on MiTeGen Micromount loops, and quench-cooled with an Oxford Cryosystems open flow N_2 cooling device.[5],[6] Specific data collection details are given in *Table S1-7*. Data processing involved the software CrysAlisPro: unit cell refinement, SCALE3 ABSPACK inter-frame scaling, merging equivalent reflections, and diffraction pattern processing. Structures were solved using SHELXT and refined with SHELXL in OLEX2.[7-9] Crystallographic data is included in the supplementary CIF files (2356741, 2356742, 2356743, 2356736, 2356737, 2356738 and 2356739) available free via the Cambridge Crystallographic Data Centre: http://www.ccdc.cam.ac.uk/data_request/cif.

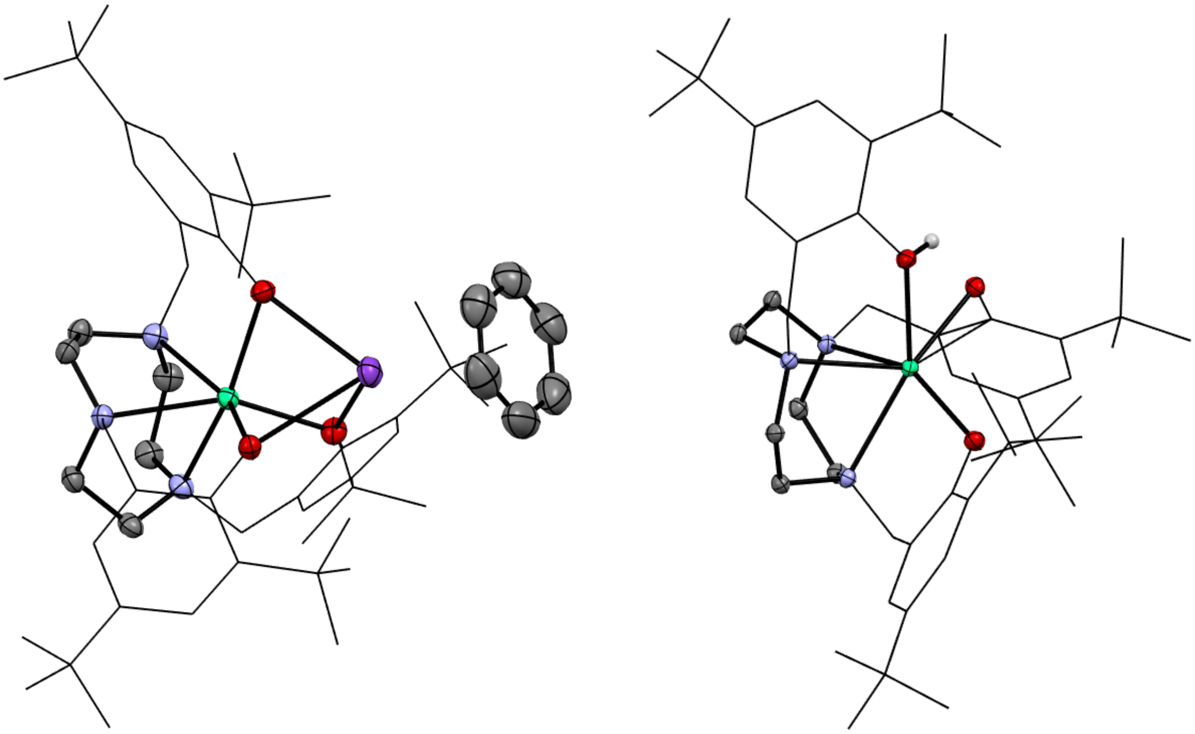

Figure S35. Molecular structures of **2** (left) and **3** (right) in the solid state as determined by X-ray crystallography (ellipsoids set at 50% probability level; H atoms, solvent molecules and counterions).

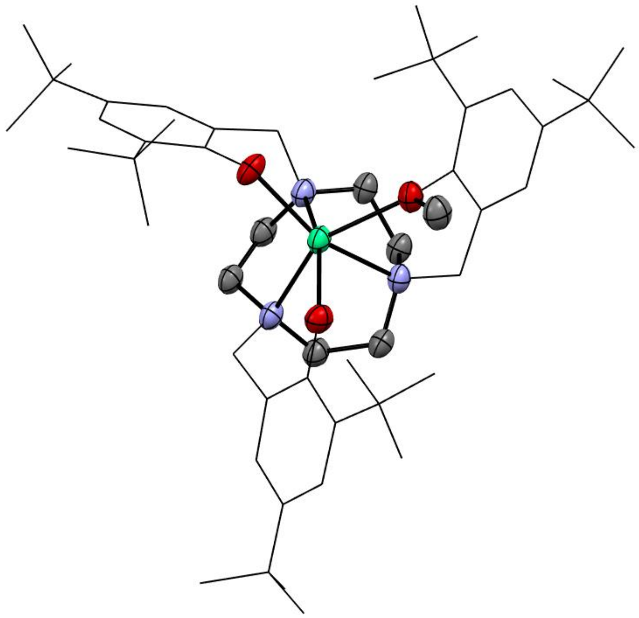

Figure S36. Molecular structure of **4** in the solid state as determined by X-ray crystallography (ellipsoids set at 50% probability level; H atoms, solvent molecules and counterions).

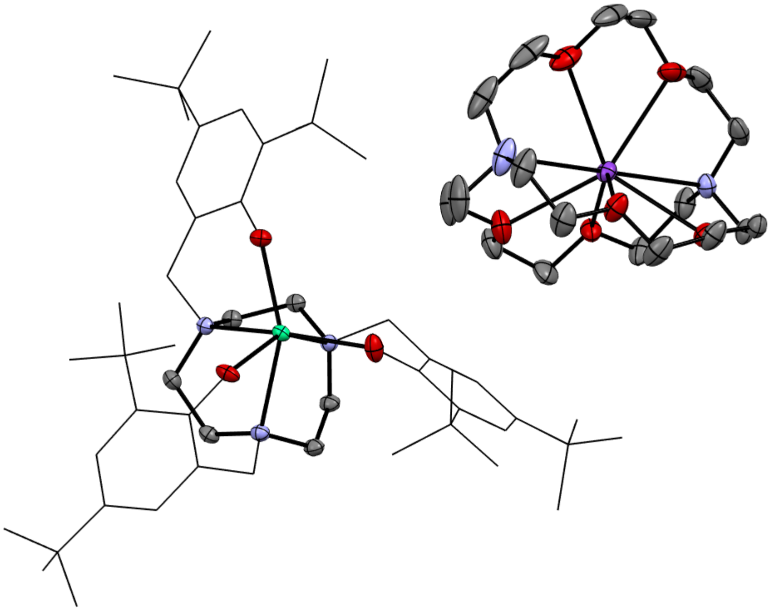

Figure S37. Molecular structure of **5** in the solid state as determined by X-ray crystallography (ellipsoids set at 50% probability level; H atoms, solvent molecules and counterions).

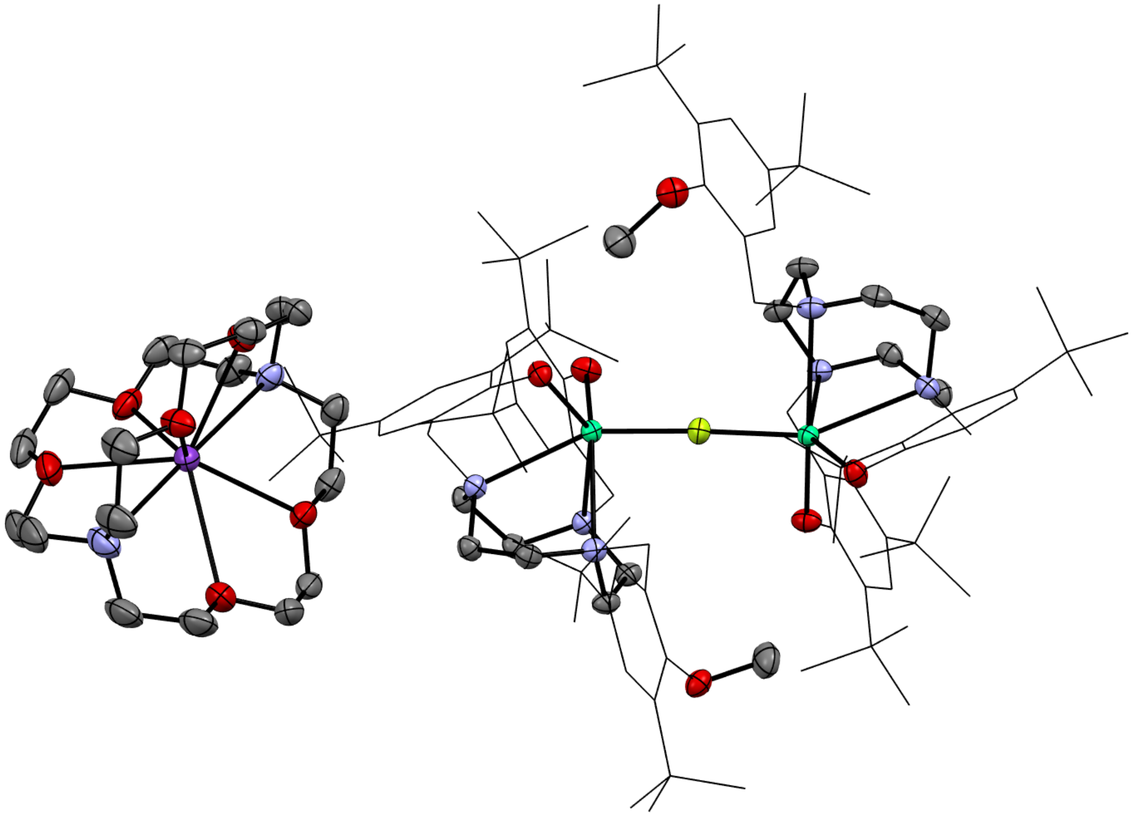

Figure S38. Molecular structure of **6** in the solid state as determined by X-ray crystallography (ellipsoids set at 50% probability level; H atoms, solvent molecules and counterions).

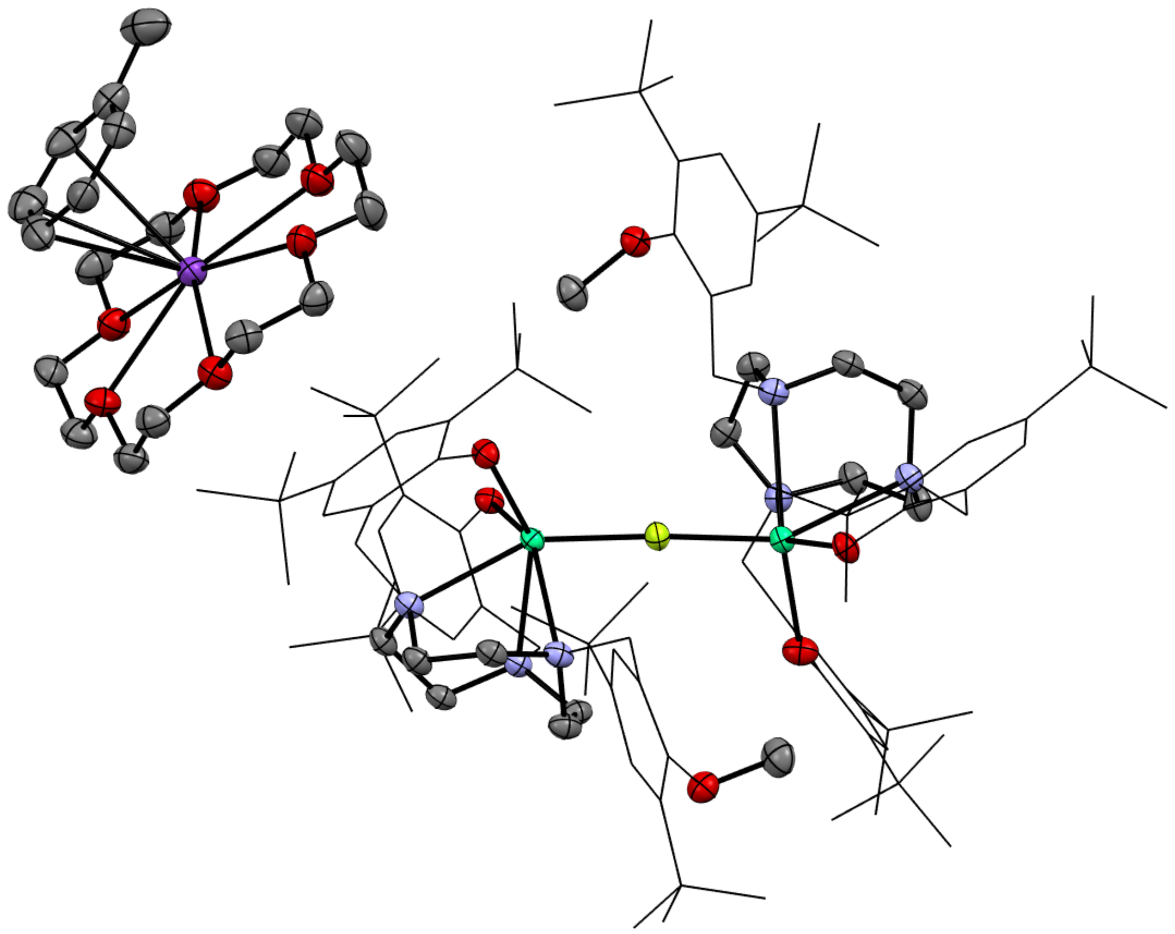

Figure S39. Molecular structure of **7** in the solid state as determined by X-ray crystallography (ellipsoids set at 50% probability level; H atoms, solvent molecules and counterions).

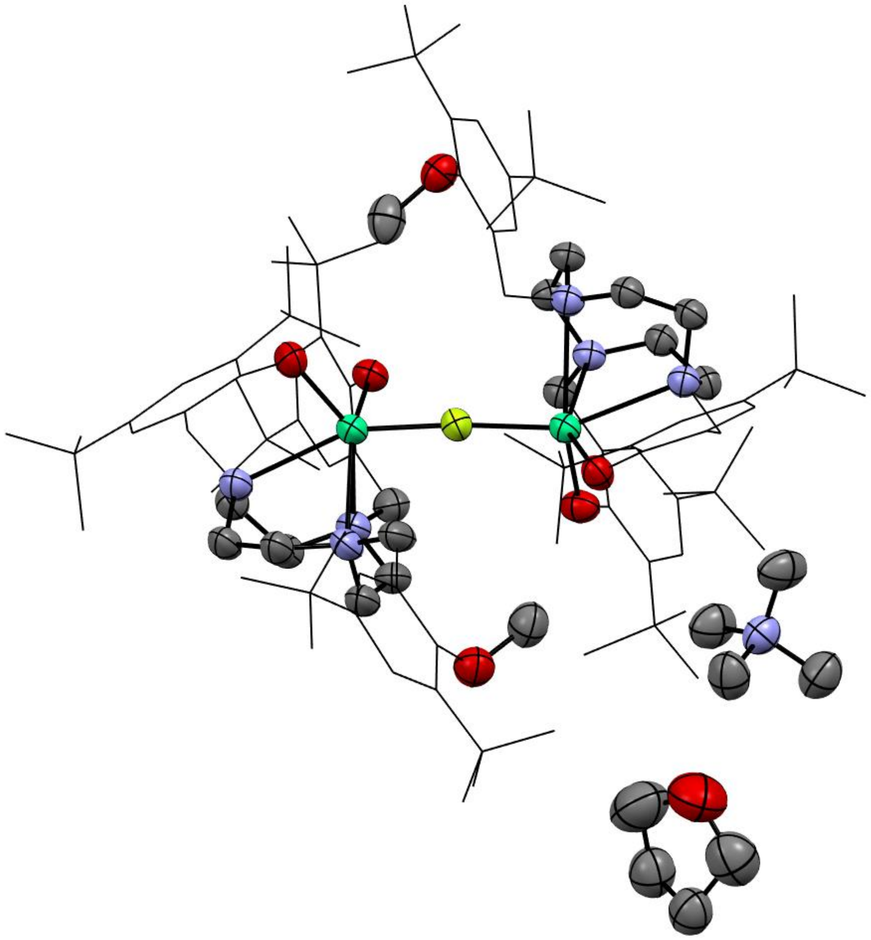

Figure S40. Molecular structure of **8** in the solid state as determined by X-ray crystallography (ellipsoids set at 50% probability level; H atoms, solvent molecules and counterions).

Table S1: **Crystallographic and refinement parameters for 2**

CCDC Number	2356741
Empirical formula	$C_{63}H_{90}CaKN_3O_3$
Formula weight	1016.56
Temperature/K	150 (10)
Crystal system	monoclinic
Space group	$P2_1/n$
a/Å	19.8547(3)
b/Å	15.1276(2)
c/Å	19.8908(3)
α/°	90
β/°	92.033(2)
γ/°	90
Volume/Å^3	5970.52(15)
Z	4
ρ_{calc}g/cm^3	1.131
μ/mm^{-1}	1.866
F(000)	2208.0
Crystal size/mm^3	0.25 × 0.25 × 0.1
Radiation	Cu Kα (λ = 1.54184)
2Θ range for data collection/°	7.344 to 153.1
Index ranges	-23 ≤ h ≤ 25, -15 ≤ k ≤ 19, -24 ≤ l ≤ 24
Reflections collected	66897
Independent reflections	12405 [R_{int} = 0.0371, R_{sigma} = 0.0248]
Data/restraints/parameters	12405/185/704
Goodness-of-fit on F^2	1.017
Final R indexes [I>=2σ (I)]	R_1 = 0.0323, wR_2 = 0.0804
Final R indexes [all data]	R_1 = 0.0407, wR_2 = 0.0866
Largest diff. peak/hole / e Å^{-3}	0.305/-0.289

Table S2: **Crystallographic and refinement parameters for 3**

CCDC Number	2356742
Empirical formula	$C_{51}H_{79}CaN_3O_3$
Formula weight	822.25
Temperature/K	100 (10)
Crystal system	monoclinic
Space group	*P*2$_1$/n
a/Å	16.89147 (14)
b/Å	15.37922 (12)
c/Å	19.54836 (17)
α/°	90
β/°	101.8707 (9)
γ/°	90
Volume/Å^3	4969.63 (7)
Z	4
ρ_{calc}g/cm^3	1.099
μ/mm^{-1}	0.168
F(000)	1800.0
Crystal size/mm^3	0.25 × 0.24 × 0.15
Radiation	Mo Kα (λ = 0.71073)
2Θ range for data collection/°	3.932 to 54.968
Index ranges	-21 ≤ h ≤ 21, -19 ≤ k ≤ 19, -25 ≤ l ≤ 25
Reflections collected	159008
Independent reflections	11378 [R_{int} = 0.0342, R_{sigma} = 0.0136]
Data/restraints/parameters	11378/360/545
Goodness-of-fit on F^2	1.033
Final R indexes [I>=2σ (I)]	R_1 = 0.0318, wR_2 = 0.0814
Final R indexes [all data]	R_1 = 0.0372, wR_2 = 0.0853
Largest diff. peak/hole / e Å^{-3}	0.335/-0.235

Table S3: **Crystallographic and refinement parameters for 4**

CCDC Number	2356743
Empirical formula	$C_{59}H_{89}CaN_3O_3$
Formula weight	928.41
Temperature/K	100(10)
Crystal system	orthorhombic
Space group	*P*na2$_1$
a/Å	39.4007(6)
b/Å	14.5413(2)
c/Å	9.82760(10)
α/°	90
β/°	90
γ/°	90
Volume/Å^3	5630.60(13)
Z	4
ρ_{calc}g/cm^3	1.095
μ/mm^{-1}	1.285
F(000)	2032.0
Crystal size/mm^3	0.1 × 0.02 × 0.02
Radiation	Cu Kα (λ = 1.54184)
2Θ range for data collection/°	4.486 to 177.588
Index ranges	-48 ≤ h ≤ 49, -18 ≤ k ≤ 18, -12 ≤ l ≤ 12
Reflections collected	163824
Independent reflections	11101 [R_{int} = 0.0935, R_{sigma} = 0.0296]
Data/restraints/parameters	11101/483/605
Goodness-of-fit on F^2	1.042
Final R indexes [I>=2σ (I)]	R_1 = 0.0772, wR_2 = 0.2075
Final R indexes [all data]	R_1 = 0.0846, wR_2 = 0.2146
Largest diff. peak/hole / e Å^{-3}	0.768/-0.394

Table S4: **Crystallographic and refinement parameters for 5**

CCDC Number	2356736
Empirical formula	$C_{76}H_{122}CaKN_5O_9$
Formula weight	1236.83
Temperature/K	100(10)
Crystal system	monoclinic
Space group	C2/c
a/Å	23.32270(5)
b/Å	16.20159(4)
c/Å	42.30145(10)
α/°	90
β/°	96.2766(2)
γ/°	90
Volume/Å^3	15888.41(6)
Z	8
ρ_{calc}g/cm^3	1.034
μ/mm^{-1}	1.540
F(000)	5392.0
Crystal size/mm^3	0.2 × 0.06 × 0.05
Radiation	Cu Kα (λ = 1.54184)
2Θ range for data collection/°	6.656 to 151.798
Index ranges	-29 ≤ h ≤ 29, -20 ≤ k ≤ 20, -52 ≤ l ≤ 46
Reflections collected	344516
Independent reflections	16336 [R_{int} = 0.0258, R_{sigma} = 0.0076]
Data/restraints/parameters	16336/21/815
Goodness-of-fit on F^2	1.021
Final R indexes [I>=2σ (I)]	R_1 = 0.0347, wR_2 = 0.0869
Final R indexes [all data]	R_1 = 0.0352, wR_2 = 0.0873
Largest diff. peak/hole / e Å^{-3}	0.303/-0.262

Table S5: **Crystallographic and refinement parameters for 6**

CCDC Number	2356737
Empirical formula	$C_{161}H_{237}Ca_2FKN_8O_{12}$
Formula weight	2614.83
Temperature/K	150.01(10)
Crystal system	triclinic
Space group	*P* $\bar{1}$
a/Å	17.6508(2)
b/Å	20.0851(3)
c/Å	24.0778(3)
α/°	96.9760(10)
β/°	91.4070(10)
γ/°	113.6790(10)
Volume/Å^3	7734.20(18)
Z	2
ρ_{calc}g/cm^3	1.123
μ/mm^{-1}	1.349
F(000)	2846.0
Crystal size/mm^3	0.1 × 0.08 × 0.05
Radiation	Cu Kα (λ = 1.54184)
2Θ range for data collection/°	7.422 to 153.632
Index ranges	-22 ≤ h ≤ 22, -25 ≤ k ≤ 24, -30 ≤ l ≤ 30
Reflections collected	166830
Independent reflections	32009 [R_{int} = 0.0433, R_{sigma} = 0.0264]
Data/restraints/parameters	32009/1684/1905
Goodness-of-fit on F^2	1.012
Final R indexes [I>=2σ (I)]	R_1 = 0.0411, wR_2 = 0.1063
Final R indexes [all data]	R_1 = 0.0535, wR_2 = 0.1158
Largest diff. peak/hole / e Å^{-3}	0.346/-0.495

Table S6: **Crystallographic and refinement parameters for 7**

CCDC Number	2356738
Empirical formula	$C_{158}H_{234}Ca_2FKN_6O_{12}$
Formula weight	2547.76
Temperature/K	100.00(10)
Crystal system	orthorhombic
Space group	$P2_12_12_1$
a/Å	15.49960(10)
b/Å	28.53030(10)
c/Å	33.74380(10)
α/°	90
β/°	90
γ/°	90
Volume/Å^3	14921.79(12)
Z	4
ρ_{calc}g/cm^3	1.134
μ/mm^{-1}	1.382
F(000)	5552.0
Crystal size/mm^3	0.06 × 0.05 × 0.04
Radiation	Cu Kα (λ = 1.54184)
2Θ range for data collection/°	4.056 to 159.896
Index ranges	-19 ≤ h ≤ 19, -28 ≤ k ≤ 35, -41 ≤ l ≤ 42
Reflections collected	133632
Independent reflections	30003 [R_{int} = 0.0272, R_{sigma} = 0.0224]
Data/restraints/parameters	30003/648/1823
Goodness-of-fit on F^2	1.043
Final R indexes [I>=2σ (I)]	R_1 = 0.0345, wR_2 = 0.0959
Final R indexes [all data]	R_1 = 0.0366, wR_2 = 0.0971
Largest diff. peak/hole / e Å^{-3}	0.341/-0.348

Table S7: **Crystallographic and refinement parameters for 8**

CCDC Number	2356739
Empirical formula	$C_{150.5}H_{226}Ca_2FKN_7O_7$
Formula weight	2344.53
Temperature/K	100.00(10)
Crystal system	monoclinic
Space group	$P2_1/n$
a/Å	15.5878(2)
b/Å	29.2064(3)
c/Å	31.0854(5)
α/°	90
β/°	98.9410(10)
γ/°	90
Volume/Å^3	13980.1(3)
Z	4
ρ_{calc}g/cm^3	0.873
μ/mm^{-1}	1.048
F(000)	4032.0
Crystal size/mm^3	0.29 × 0.04 × 0.02
Radiation	Cu Kα (λ = 1.54184)
2Θ range for data collection/°	4.176 to 152.19
Index ranges	-19 ≤ h ≤ 19, -30 ≤ k ≤ 36, -38 ≤ l ≤ 38
Reflections collected	233112
Independent reflections	28661 [R_{int} = 0.0922, R_{sigma} = 0.0424]
Data/restraints/parameters	28661/282/1288
Goodness-of-fit on F^2	1.068
Final R indexes [I>=2σ (I)]	R_1 = 0.0990, wR_2 = 0.3004
Final R indexes [all data]	R_1 = 0.1167, wR_2 = 0.3161
Largest diff. peak/hole / e Å^{-3}	1.110/-0.557

References

[1] H. W. Roesky, K. Keller, *J. Fluor. Chem.* **1998**, *89*, 3–4.
[2] T. K. Panda, C. G. Hrib, P. G. Jones, J. Jenter, P. W. Roesky, M. Tamm, *Eur. J. Inorg. Chem.* **2008**, *2008*, 4270–4279.
[3] B. Adam, E. Bill, E. Bothe, B. Goerdt, G. Haselhorst, K. Hildenbrand, A. Sokolowski, S. Steenken, T. Weyhermüller, K. Wieghardt, *Chem.–Eur. J.* **1997**, *3*, 308–319.
[4] R. Neufeld, D. Stalke, *Chem. Sci.*, **2015**, *6*, 3354–3364.
[5] J. Cosier, A. M. Glazer, *J. Appl. Cryst.* **1986**, *19*, 105.
[6] CrysAlisPro, Agilent Technologies, Version 1.171.39.46.
[7] G. M. Sheldrick, *Acta Crystallogr., Sect. A: Found. Adv.* **2015**, *71*, 3.
[8] G. M. Sheldrick, *Acta Crystallogr., Sect. C: Struct. Chem*. **2015**, *71*, 3.
[9] O. V. Dolomanov, L. J. Bourhis, R. J. Gildea, J. A. K. Howard, H. Puschmann, *J. Appl. Cryst.* **2009**, *42*, 339.

.
